# The protein kinase DYRK1B is a p53 target gene and functions as a negative feedback regulator of the transcription factor RFX7

**DOI:** 10.1038/s41419-026-08660-x

**Published:** 2026-03-26

**Authors:** Gerrit Wilms, Katharina Schwandt, Stefan Düsterhöft, Philip Helmich, Justyna Wozniak, Florian Kraft, Sebastian Kallabis, Felix Meissner, Walter Becker

**Affiliations:** 1 Institute of Pharmacology and Toxicology, RWTH Aachen University, Aachen, Germany; 2 Institute of Molecular Pharmacology, RWTH Aachen University, Aachen, Germany; 3 Institute of Clinical Pharmacology, University Hospital of RWTH Aachen, Aachen, Germany; 4 Medical Faculty, Center for Human Genetics and Genomic Medicine, RWTH University Aachen, Aachen, Germany; 5 Department of Systems Immunology & Proteomics, Institute of Innate Immunity, Medical Faculty, University of Bonn, Bonn, Germany

**Keywords:** DNA damage and repair, Lung cancer, Target identification

## Abstract

The tumor suppressor protein p53 orchestrates cellular responses to stress by regulating the transcription of target genes involved in processes such as cell cycle control, DNA damage repair and apoptosis. The protein kinase DYRK1B, known to promote cancer cell survival and contribute to DNA damage repair, is overexpressed in various tumor types. Here, we demonstrate that expression of DYRK1B - but not its closely related paralog DYRK1A - is upregulated by cytostatic drugs (Actinomycin D, Doxorubicin) in multiple cancer cell lines. This induction required functional p53 and was mediated by p53-dependent activation of the transcription factor RFX7. Furthermore, we show that DYRK1B physically interacts with RFX7 and counteracts its activation by p53, thereby establishing a negative feedback loop that attenuates RFX7-dependent gene expression. This inhibitory effect of DYRK1B was strictly dependent on its catalytic activity and could be blocked by using small-molecule DYRK1 inhibitors. In conclusion, our study identifies DYRK1B as an indirect p53 target that suppresses p53-mediated activation of RFX7. These findings suggest that pharmacological inhibition of DYRK1B may represent a therapeutic strategy to enhance RFX7 tumor suppressor function.

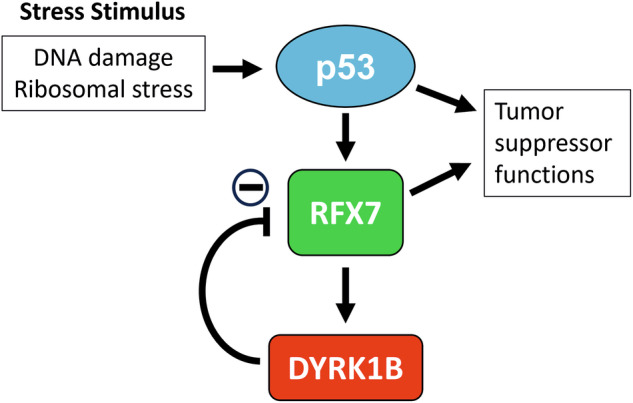

## Introduction

The transcription factor p53 (encoded by *TP53*) is a master regulator for maintaining genomic integrity in response to various stress stimuli, including DNA damage and ribosome biogenesis stress [1–4]. Under basal conditions, p53 protein levels are kept low by the E3 ubiquitin ligase MDM2 (murine double minute 2), which drives its ubiquitination and subsequent proteasomal degradation [1, 5, 6]. In response to genotoxic stress, p53 is stabilized and coordinates an extensive gene regulatory network [7–9]. The resulting changes in gene expression regulate and coordinate a range of cellular responses, including cell cycle arrest, DNA damage repair, the induction of apoptosis, or cellular senescence [3, 10]. The fact that the vast majority of cancer-associated *TP53* mutations disrupt the DNA-binding domain highlights the critical role of p53’s transcriptional activity in tumor suppression [11].

Beyond directly activating target genes through binding to specific promoter elements, p53 also modulates gene expression indirectly *via* downstream transcription factors, such as the DREAM (DP, RB-like, E2F, and MuvB) complex, or RFX7 (regulatory factor X7) [12, 13]. RFX7 is a winged helix transcription factor that binds to X-box promoter motifs and is recurrently altered or functionally inactivated in cancer. RFX7 has recently been identified as a key downstream effector of p53 that regulates a tumor-suppressive transcriptional program [13].

The protein kinase DYRK1B (Dual-specificity tyrosine phosphorylation-regulated kinase 1B) functions as a pro-survival factor in cellular stress conditions, promotes cell cycle exit and maintains cancer cell quiescence, and coordinates DNA damage repair [14–17]. In many cancer cell lines, pharmacological inhibition of DYRK1B or genetic depletion by RNA interference has been shown to reduce cancer cell survival and to increase the sensitivity to cytotoxic drugs [18–23] or irradiation [24]. Beyond these cell-autonomous mechanisms, DYRK1B also influences the tumor microenvironment and facilitates immune evasion of pancreatic cancer cells [25, 26]. Notably, the combination of a DYRK1B inhibitor with cytostatic drugs significantly prolonged overall survival in a highly aggressive mouse model of pancreatic cancer [25].

Importantly, DYRK1B has been found to be overexpressed in various solid tumors, including pancreatic cancer, ovarian cancer, lung cancer and breast cancer [14, 15, 18, 20, 27]. Notably, the expression level of DYRK1B translates directly to its cellular activity, as DYRK kinases maintain constitutive catalytic activity following cotranslational auto-activation [28, 29]. Genomic amplification of the *DYRK1B* gene has been observed in several tumor types, further suggesting that elevated DYRK1B levels confer a selection advantage to cancer cells [30, 31].

These findings collectively underscore the pro-oncogenic functions of DYRK1B and support its classification as a potential cancer drug target. Nevertheless, it remains categorized as a ‘dark kinase’, due to the limited functional annotations available on its regulation and function in cancer [32].

In this study, we characterize DYRK1B as a novel downstream target of the p53 stress pathway. Furthermore, we demonstrate that p53 induces DYRK1B upregulation indirectly through the activation of RFX7 and investigate the role of DYRK1B as a regulator of RFX7 function.

## Results

### DYRK1B expression is elevated in human cancers

Given that both DYRK1B and its closely related paralog, DYRK1A, have been implicated in cancer cell survival and DNA damage response [14, 15, 31, 33–38], we used the pan-cancer analysis tool of the TNMplot database [39] to compare the relative mRNA levels of *DYRK1A* and *DYRK1B* in normal and tumor tissues. This analysis revealed that expression of *DYRK1B*, but not *DYRK1A*, was significantly increased in tumor *vs*. normal samples in 17 of 22 types of cancer (Fig. S1).

### DYRK1B expression is increased in response to Doxorubicin and Actinomycin D

**Fig. 1:**
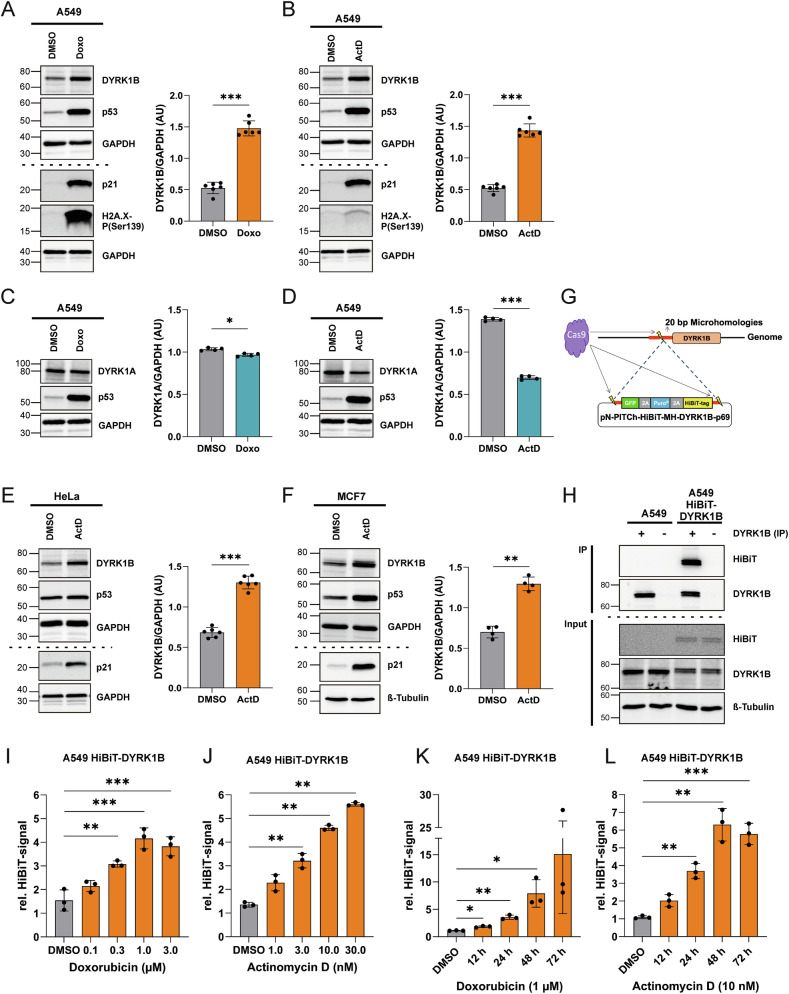
Upregulation of DYRK1B by cytostatic drugs. A549, HeLa, or MCF7 cells were treated for 24 h with 1 µM Doxorubicin (Doxo) (**A**, **C**) or 10 nM Actinomycin D (ActD) (**B**, **D**–**F**). DMSO served as the vehicle control. Total cell lysates were separated by SDS-PAGE on two parallel gels (10%, upper part, and 16%, lower part) and analyzed by western blotting using the indicated antibodies. GAPDH and β-tubulin served as loading controls. The decrease of DYRK1A in (**D**) is likely due to the inhibitory effect of ActD on RNA polymerase II-dependent transcription. Column diagrams show the densitometric evaluation of 4-6 independent experiments (mean ± SD). AU, arbitrary units. **G** The CRISPR/Cas9-based PITCh technique (Precise Integration into Target Chromosome) was used to integrate a knock-in cassette comprising GFP, a puromycin resistance gene and the HiBiT-tag in the DYRK1B locus. GFP, puromycin acetyl-transferase and HiBiT are separated by 2A self-cleaving peptides. **H** Functional tagging was confirmed by immunoprecipitating DYRK1B from the parental A549 cells and A549 HiBiT-DYRK1B cells with a DYRK1B-reactive serum (+). Non-reactive serum (–) served as a negative control. Aliquots of the whole cell lysates (input) and immunoprecipitated proteins (IP) were analyzed by western blotting using the indicated antibodies or the HiBiT-blotting detection system. A549 HiBiT-DYRK1B cells were treated with increasing concentrations of Doxorubicin (**I**) or Actinomycin D (**J**) for 24 h or were treated for variable times with 1 µM Doxorubicin (**K**) or 10 nM Actinomycin D (**L**). Apparent cytotoxicity precluded the use of higher concentrations. HiBiT-DYRK1B protein levels were measured by HiBiT-Lytic assay in total cell lysates and normalized to total protein (*n* = 3, means ± SD). Statistically significant differences to the control samples are indicated by asterisks (* *p* < 0.05, ** *p* < 0.01, *** *p* < 0.001).

We used the A549 lung adenocarcinoma cell line as our primary model system to explore the regulation of DYRK1B in cancer cells. DYRK1B levels are low under basal conditions in A549 cells but are elevated in response to environmental stimuli such as serum deprivation and cell density [40]. Treatment of A549 cells with Doxorubicin or Actinomycin D substantially increased expression of DYRK1B but not DYRK1A (Fig. 1A–D). Both drugs activated p53 and upregulated its paradigmatic downstream target, CDK inhibitor p21 (a.k.a WAF1 or Cip1). Phosphorylation of histone H2AX at serine 139 (a.k.a. γH2AX), an established marker for DNA damage [41], was markedly elevated in Doxorubicin-treated cells. In contrast, Actinomycin D only modestly enhanced the γH2AX signal, as it primarily triggers ribosome biogenesis stress at low concentrations rather than directly causing DNA damage [4]. Actinomycin D also induced DYRK1B expression in two other cancer cell lines, HeLa cervix carcinoma and MCF7 breast cancer cells (Fig. 1E, F).

To further investigate DYRK1B expression, we generated a genetically modified A549 cell line expressing endogenous DYRK1B as a fusion protein with an N-terminal HiBiT tag (Fig. 1G, H). Proteins tagged with the 11-amino acid HiBiT peptide can be quantified by fragment-complementation luciferase assays [42]. Both Doxorubicin and Actinomycin D induced a concentration-dependent increase of DYRK1B levels, with maximal stimulation reaching approximately threefold (Fig. 1I, J). Prolonged exposure to either drug for two more days further enhanced DYRK1B levels (Fig. 1K, L).

### DYRK1B expression is upregulated in response to p53 activation

**Fig. 2:**
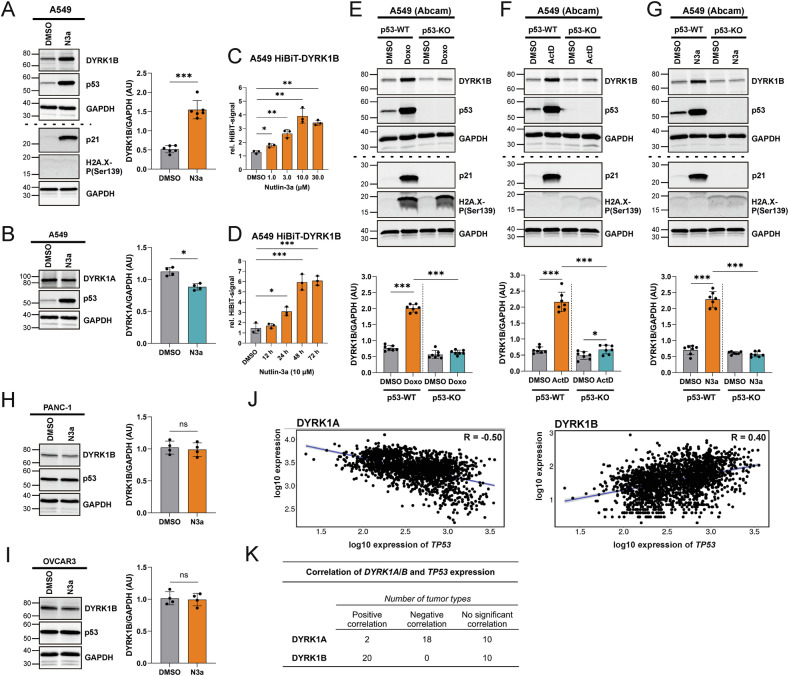
DYRK1B is a p53 target gene. **A**, **B** A549 cells were treated with 10 µM Nutlin-3a (N3a) for 24 h. Total cell lysates were separated on two parallel gels (10 and 16%) and analyzed by western blotting as indicated (*n* = 4–6, means ± SD). A549 HiBiT-DYRK1B cells were treated with variable concentrations of Nutlin-3a for 24 h (**C**) or with 10 µM Nutlin-3a for variable times (**D**). DYRK1B levels were quantified by HiBiT-Lytic assay and normalized to total protein contents of the samples (*n* = 3, means ± SD). Parental A549 cells (WT) and p53-KO cells were treated with 1 µM Doxorubicin (Doxo) (**E**), 10 nM Actinomycin D (ActD) (**F**) or 10 µM Nutlin-3a (N3a) (**G**) for 24 h and analysed by western blotting as above (*n* = 7, means ± SD). PANC-1 cells (**H**) and OVCAR3 cells (**I**) were treated with 10 µM Nutlin-3a (N3a) for 24 h. DYRK1B levels were analysed by western blotting (*n* = 4, means ± SD). **J** The scatter plots illustrate the correlation between *TP53* mRNA levels and *DYRK1A* (left) or *DYRK1B* expression (right) in human lung tumor samples. The correlation analysis (*n* = 1865) was performed using TNMplot.com [39]. *p*-values and correlation coefficients (R) were determined using Spearman correlation. **K** The table summarizes the results of TNMplot correlation analyses for 30 tumor types. Individual *p*-values and correlation coefficients (R) are listed in Table S1. Correlations with *p* < 0.05 were considered significant. Asterisks indicate statistically significant differences in the column diagrams (* *p* < 0.05, ** *p* < 0.01, *** *p* < 0.001, ns *p* > 0.05). AU, arbitrary units.

The parallel increase of DYRK1B and p53 levels in response to treatment with Doxorubicin and Actinomycin D led us to hypothesize that DYRK1B is a p53 target gene. To specifically stimulate p53 signaling without activating other cell stress pathways, we used Nutlin-3a, a small molecule drug that disrupts the MDM2-p53 interaction, which results in p53 accumulation [43]. As expected, Nutlin-3a treatment of A549 cells substantially increased p53 and p21 protein levels without affecting the γH2AX signal (Fig. 2A, B). Furthermore, Nutlin-3a induced the expression of DYRK1B, but not DYRK1A, in a time- and concentration-dependent manner (Fig. 2A–D).

To scrutinize the role of p53 as a regulator of DYRK1B expression, we used A549 *TP53* knockout (p53-KO) cells. The absence of p21 induction following treatment with Doxorubicin, Actinomycin D or Nutlin-3a confirmed the functional inactivation of the *TP53* gene (Fig. 2E–G). The upregulation of DYRK1B by all three p53 activators was abolished in A549 p53-KO cells, confirming that DYRK1B is a downstream target of p53. Furthermore, Nutlin-3a did not affect DYRK1B levels in two different cancer cell lines (OVCAR3 and PANC-1) with inactivating mutations of the *TP53* gene (Fig. 2H, I).

To explore the relationship between DYRK1B expression and p53 in clinical tumor samples, we analyzed publicly available gene chip data using Spearman correlation analysis (Fig. 2J). In lung tumors, *TP53* mRNA levels showed a positive correlation with *DYRK1B* expression but a negative correlation with *DYRK1A*. Beyond lung tumors, a positive correlation of *DYRK1B* and *TP53* mRNA levels was observed in 20 of 30 tumor types (Fig. 2K and Table S1). Notably, this analysis most likely underestimates the association between *DYRK1B* expression and functional p53, as *TP53* mutation status was not considered.

### RFX7 mediates DYRK1B upregulation

**Fig. 3:**
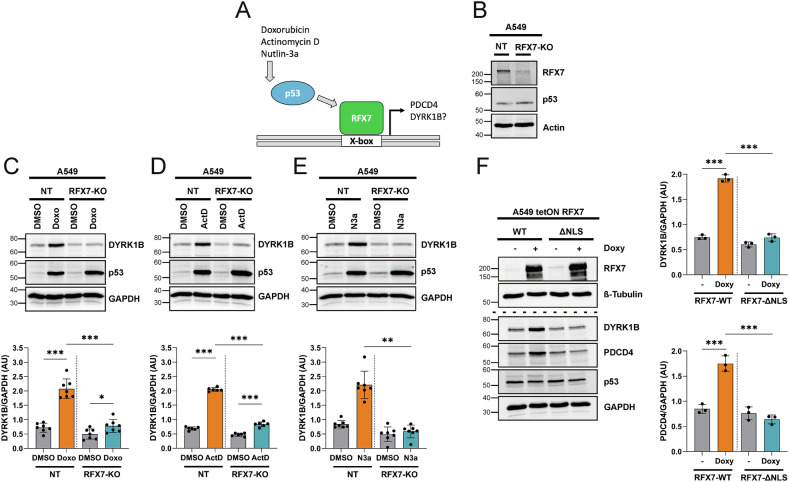
DYRK1B is an RFX7 target gene. **A** Schematic illustration of the p53-RFX7 signaling axis. *PDCD4* is transcriptionally upregulated by the binding of RFX7 to an X-box element in its promoter [13]. **B** A549 RFX7-KO cells were created using a lentiviral CRISPR/Cas9 knockout system. A control cell line was generated by using a non-targeting (NT) sgRNA. RFX7-dependent upregulation of DYRK1B. A549 RFX7-KO or -NT cells were treated with Doxorubicin (Doxo) (**C**), Actinomycin D (ActD) (**D**) or Nutlin-3a (N3a) (**E**) for 24 h. Cell lysates were analyzed by western blotting (*n* = 6–7, means ± SD). **F** RFX7 overexpression. A549 cells with stable, tetracycline-inducible overexpression of murine RFX7 were created by lentiviral infection. RFX7 overexpression was induced by treatment with doxycycline for 24 h (*n* = 3, means ± SD). AU, arbitrary units. Statistically significant differences are indicated by asterisks (* *p* < 0.05, ** *p* < 0.01, *** *p* < 0.001).

RFX7 is a major downstream mediator of the p53 gene regulatory program (Fig. 3A) [13]. To investigate whether p53 upregulates DYRK1B *via* RFX7 activation, we created A549 RFX7-KO cells. Residual RFX7 immunoreactivity indicates incomplete RFX7 knockout in the cell pool (Fig. 3B). The upregulation of DYRK1B in response to Doxorubicin, Actinomycin D, or Nutlin-3a was strongly reduced in A549 RFX7-KO cells, while p53 accumulation remained unaffected by RFX7 inactivation (Fig. 3C–E).

Next, we asked whether elevated levels of RFX7 are sufficient to increase the DYRK1B expression in the absence of p53-activating stimuli. To this end, we generated A549 cells with stable, tetracycline-inducible overexpression of wild-type RFX7 (RFX7^WT^). As a negative control, we utilized a mutant version of RFX7 with a disrupted nuclear localization signal (RFX7^ΔNLS^), which lacks transactivating activity [44]. Overexpression of RFX7^WT^, but not RFX7^ΔNLS^, significantly increased DYRK1B levels. The effect was comparable to the upregulation of tumor suppressor protein PDCD4, a well-established direct target of RFX7 (Fig. 3F) [45, 46]. Taken together, these results classify *DYRK1B* as an indirect p53-responsive gene whose expression is controlled by RFX7.

### Expression of *DYRK1B* is upregulated on mRNA level

**Fig. 4:**
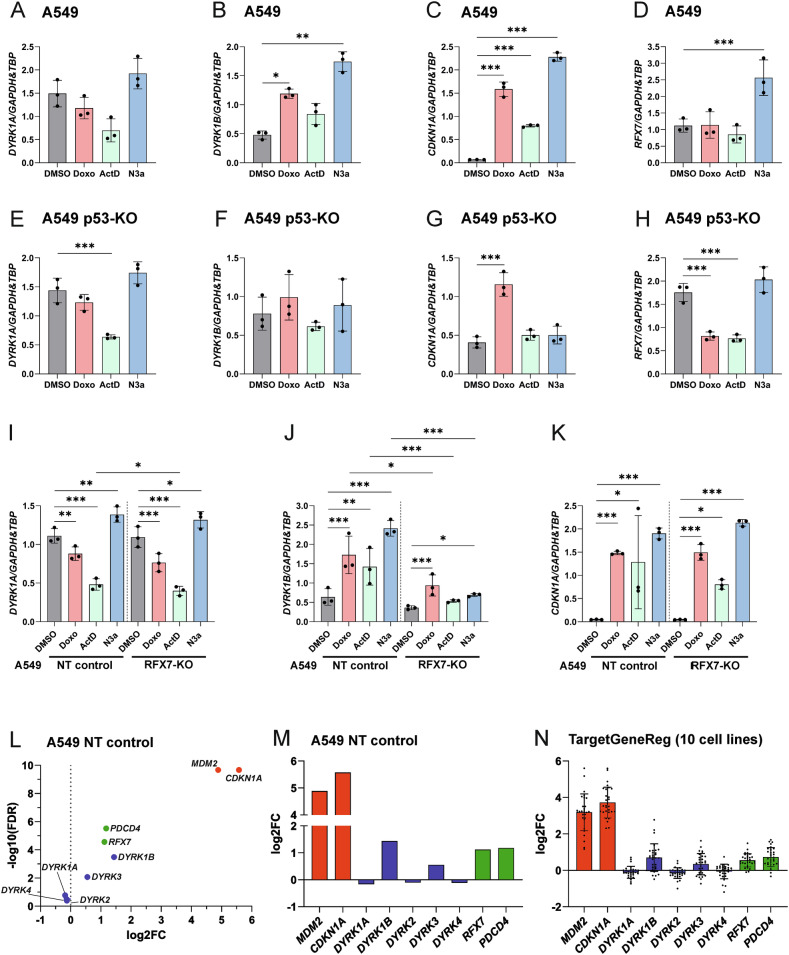
DYRK1B expression is regulated on mRNA level. RT-qPCR. A549 cells were treated with 1 µM Doxorubicin (Doxo), 10 nM Actinomycin D (ActD) or 10 µM Nutlin-3a (N3a) for 24 h. mRNA levels of *DYRK1A*, *DYRK1B*, *CDKN1A*, and *RFX7* were analyzed by RT-qPCR and quantified relative to *GAPDH* and *TBP* mRNA (*n* = 3, means ± SD). **A**–**D** A549 wild type cells, **E**–**H** A549 p53-KO cells, **I**–**K** A549 RFX7-KO cells and the corresponding A549-NT control cells. Statistical differences to the control are indicated by asterisks (* *p* < 0.05, ** *p* < 0.01, *** *p* < 0.001). RNA-seq analysis. A549-NT cells were treated with DMSO (*n* = 3) or 10 µM Nutlin-3a (N3a) (*n* = 4) for 24 h. The volcano plot (**L**) and the column diagram (**M**) illustrate logarithmic fold changes (log2FC) of select mRNAs in response to Nutlin-3a. A table of Log2FC and adjusted *p* values is provided in the supplementary information (Table S2). **N** Analysis of mRNA data in the TargetGeneRegulation Database 2.0 [47]. Columns show fold changes (FC) of select mRNAs in response to MDM2 inhibition (means ± SD). The diagram includes 29 datasets collected from ten different cell lines (NB1691, AG22153, MCF10A, IMR90, HepG2, U2OS, RPE1, HCT116, MCF7, SJSA). Data from genetically modified cell lines were excluded.

Next, we explored changes in *DYRK1A* and *DYRK1B* mRNA levels in response to p53-activating stimuli. Overall, the observed treatment effects closely mirrored corresponding changes in protein levels (Fig. 4A–K). Specifically, treatment with Doxorubicin, Actinomycin D or Nutlin-3a increased *DYRK1B* mRNA levels in parental A549 cells. This effect was abolished in A549 p53-KO cells (Fig. 4F) and significantly reduced in A549 RFX7-KO cells (Fig. 4J). As expected, *CDKN1A* (encoding p21), a well-established direct target of p53, remained unaffected by RFX7 knockout. Consistent with previous findings in other cell lines [13], *RFX7* mRNA was upregulated by Nutlin-3a treatment in a p53-dependent manner (Fig. 4D, H). It should be noted that Doxorubicin and Actinomycin D can suppress global mRNA transcription, which likely explains the reduction of mRNA levels observed in several samples.

To determine whether other members of the DYRK family are regulated by p53 signaling, we analyzed RNA-seq data from Nutlin-3a-treated and untreated A549-NT cells. *DYRK1B* mRNA was upregulated to a similar extent as *PDCD4* and *RFX7* (Fig. 4L, M and Table S2). *DYRK3* mRNA showed a more moderate increase, while the expression of the other DYRK family members remained largely unchanged.

To extend this analysis to additional tumor types, we utilized the TargetGeneReg 2.0 web atlas [47] to compare the effects of MDM2 inhibition across 10 different cell lines (Fig. 4N). The observed expression pattern closely mirrors the results in A549 cells, supporting the conclusion that DYRK1B upregulation in response to p53 activation is a common feature in various cell lines.

### DYRK1B negatively regulates p53-RFX7 signaling

**Fig. 5:**
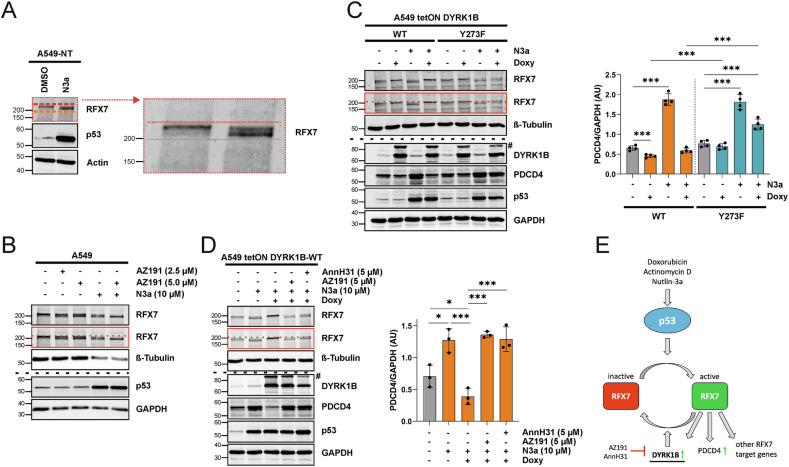
DYRK1B is a negative regulator of p53-RFX7 signaling. **A** p53-mediated activation of RFX7. A549-NT cells were treated for 24 h with 10 µM Nutlin-3a to activate p53. The cropped enlargement highlights the Nutlin-3a-induced change in RFX7 electrophoretic mobility. **B** Effect of DYRK1B inhibition. A549 cells were treated for 24 h as indicated and analyzed by western blotting. The RFX7 bands are shown as a close-up to illustrate the RFX7 band shift (horizontal red dashed line). The image is representative for *n* = 3 independent experiments. Effect of DYRK1B overexpression. A549 tetON DYRK1B-WT or DYRK1B-Y273F cells were treated as indicated for 24 h. PDCD4 levels were densitometrically quantified (*n* = 4 in (**C**), *n* = 3 in (**D**), means ± SD). The band marked by the hashtag is due to the incomplete cleavage of GFP and DYRK1B. AU, arbitrary units. Statistically significant differences are indicated by asterisks (* *p* < 0.05, ** *p* < 0.01, *** *p* < 0.001). **E** Scheme illustrating the proposed feedback regulation of RFX7 activity through DYRK1B induction. Pharmacological inhibition of overexpressed DYRK1B restores RFX7 signaling in A549 cells.

Activation of RFX7 is associated with a change in its electrophoretic mobility [13]. Under basal conditions, RFX7 migrates as a double band consisting of a predominant upper band and a much fainter lower band (Fig. 5A). Activation of p53 by Nutlin-3a increased the intensity of the faster-migrating band, which represents the transcriptionally active form of RFX7 [13]. Interestingly, treatment of A549 cells with the DYRK1B inhibitor AZ191 [48] led to an increase in the faster migrating form of RFX7, similar to the effect of p53 activation by Nutlin-3a (Fig. 5B). Of note, AZ191 did not affect p53 protein levels. Combined treatment with AZ191 and Nutlin-3a resulted in the almost complete shift from the upper to the lower RFX7 band. Thus, we concluded that the catalytic activity of DYRK1B counteracts the p53-induced downshift of the upper RFX7 band and thereby functions as a negative feedback regulator of RFX7 activation.

To further examine the effect of DYRK1B on RFX7, we used A549 cells with stable, tetracycline-inducible overexpression of DYRK1B [49]. As a measure of RFX7 transcriptional activity, we quantified cellular PDCD4 levels (Fig. 5C, D). In the absence of Nutlin-3a, DYRK1B overexpression completely converted RFX7 to its slower-migrating form and caused a slight reduction in PDCD4 levels. In the presence of Nutlin-3a, DYRK1B overexpression fully reversed the p53-mediated downshift of RFX7 and blocked PDCD4 upregulation. This effect of DYRK1B was significantly reduced in a control cell line expressing the kinase-impaired DYRK1B-Y273F point mutant. These results indicate that DYRK1B negatively regulates the p53-RFX7 signaling pathway.

Next, we explored the possibility of pharmacologically reactivating the tumor suppressor protein RFX7 in cells with elevated DYRK1B levels. A549-DYRK1B^WT^ cells were co-treated with Nutlin-3a to stimulate p53-dependent RFX7 activation, and with doxycycline to induce DYRK1B overexpression, which suppressed the p53-RFX7-mediated upregulation of PDCD4 expression (Fig. 5D). Additional treatment with either of two structurally unrelated DYRK1 inhibitors (AZ191 [48], AnnH31 [50]) restored the RFX7 band downshift and reestablished the p53-RFX7-mediated upregulation of tumor suppressor PDCD4 (Fig. 5D). These findings demonstrate that the inhibitory effect of DYRK1B on RFX7 activity can be effectively reversed through pharmacological intervention (Fig. 5E).

### DYRK1B is a negative regulator of RFX7

Pioneering ChIP and transcriptome studies by Martin Fischer and colleagues have identified a total of 90 RFX7 target genes [13, 45]. To investigate how DYRK1B regulates the proteins encoded by these genes, we performed global proteomics experiments in A549 tetON DYRK1B^WT^ cells. Cells were treated with Nutlin-3a to activate p53-RFX7 signaling and with doxycycline to induce DYRK1B overexpression. This analysis identified 6056 proteins, including 33 previously annotated as RFX7 target genes [13, 45].

**Fig. 6:**
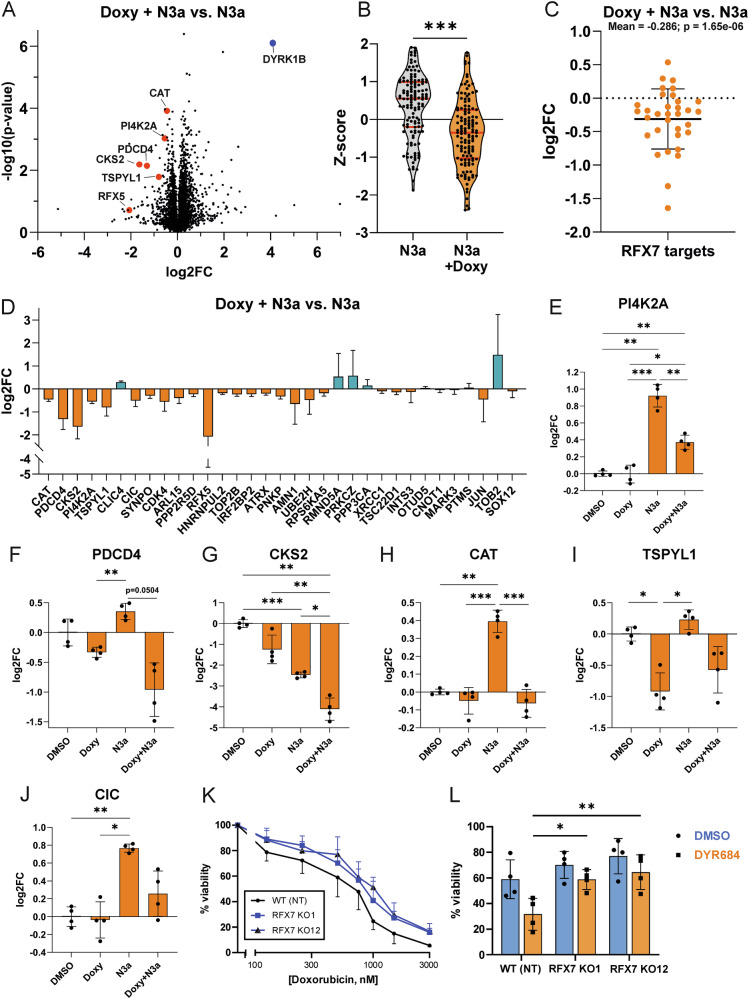
DYRK1B is a negative regulator of RFX7 function. Proteome analysis of A549 tetON DYRK1B-WT cells treated for 24 h with 10 µM Nutlin-3a and/or doxycycline as indicated (*n* = 4). **A** Volcano plot of differentially expressed proteins in cells treated with Nutlin-3a and doxycycline *vs*. cells treated only with Nutlin-3a. Select RFX7 targets are highlighted in red. DYRK1B is shown in blue. **B**–**D** Effects of DYRK1B overexpression on 33 established RFX7 targets [13, 45]. The violin plot shows the Z-score distribution, upper, median and lower quartiles. Log2 fold changes. The scatter plot (**C**) summarizes the mean differences of all 33 RFX7 targets and the column diagrams (**D**) represents the individual Log2 fold changes of the RFX7 targets relative to the mean of N3a-treated samples (*n* = 4, means ± SD). **E**–**J** Comparison of Log2 fold changes induced by the of indicated treatments for selected RFX7 targets normalized to the mean of DMSO treated cells (mean ± SD). **K** RFX7 sensitizes A549 cells to Doxorubicin. Viability assay of A549 cells 3 days post-recovery from a treatment with different concentrations of Doxorubicin for 24 h. Two clones of RFX7-KO cells (KO1, KO12) were compared with A549-NT control cells (means and SD, *n* = 4). **L** Reduced chemosensitizing effect of DYRK1 depletion in A549 RFX7-KO cells. Cells were treated with 500 nM Doxorubicin and the DYRK1 depleting PROTAC DYR684 (100 nM) as indicated (means and SD, *n* = 4). Asterisks mark statistically significant interaction effects between cell lines (WT vs. KO) and treatment, indicating that treatment responses differ between genotypes (two-way ANOVA). Statistical differences compared to the control are indicated by asterisks (* *p* < 0.05, ** *p* < 0.01, *** *p* < 0.001).

DYRK1B overexpression reduced the abundance of many RFX7 targets in Nutlin-3a-treated A549 cells (Fig. 6A–J). Notably, some targets, including PDCD4, CKS2, and TSPYL1, were downregulated by DYRK1B overexpression even in the absence of Nutlin-3a, suggesting that DYRK1B modulates basal RFX7 activity (Fig. 6F, G, I). As previously observed in the U2OS osteosarcoma cell line [45], CKS2 displayed a distinct regulatory pattern, as this gene is repressed by Nutlin-3a treatment *via* the p53-p21-DREAM pathway [51, 52].

To further explore whether DYRK1B acts as a general negative regulator of RFX7 independent of p53 activation, we performed RNA-seq analysis of A549 tetON DYRK1B^WT^ cells after long-term (7d) induction of DYRK1B overexpression, mimicking the elevated DYRK1B levels observed in many cancers. Gene set enrichment analysis revealed a significant downregulation of RFX7 target genes in DYRK1B overexpressing cells (Fig. S2A), further supporting the conclusion that DYRK1B functions as a negative regulator of RFX7 transcriptional activity.

Next, we asked whether DYRK1B modulates the function of RFX7 in the cellular stress response. To this end, we adopted the experimental strategy used by Coronel et al. [13], who demonstrated the chemosensitizing role of RFX7 in U2OS and HCT116 cells by showing that RFX7 depletion increased the viability of cells exposed to low concentrations of doxorubicin. In agreement with these findings, A549 RFX7-KO cells displayed higher viability than A549 NT cells when treated with doxorubicin (Fig. 6K).

To assess whether the effect of DYRK1B on RFX7 function is biologically relevant, we used the DYRK1-targeting PROTAC (proteolysis-targeting chimera) DYR684. We have previously shown that treatment with DYR684 effectively reduces DYRK1B levels in A549 cells [53]. Consistent with the established role of DYRK1B as a survival kinase [14], DYR684 treatment sensitized A549 NT cells to doxorubicin-induced cytotoxicity (Fig. 6L). Importantly, this chemosensitizing effect was substantially attenuated in two independent A549 RFX7-KO clones, suggesting that reactivation of RFX7 accounts, at least in part, for the enhanced drug sensitivity caused by DYRK1 depletion.

### DYRK1 kinases interact with RFX7

**Fig. 7:**
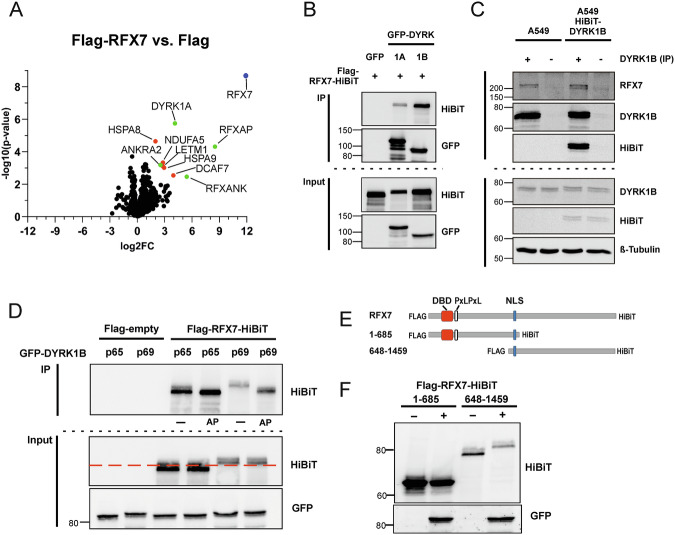
DYRK1 kinases interact with and phosphorylate RFX7. **A** RFX7 interacting proteins were identified by immunoprecipitation of Flag-RFX7 from transiently transfected HEK293 cells, followed by quantitative mass spectrometry. The volcano plot displays the fold enrichment of proteins co-precipitating with RFX7 compared to control immunoprecipitation using cells transfected with the empty Flag vector. Statistically significant interactors are labeled; the bait protein (RFX7) is highlighted in blue, and known RFX7 interactors listed in the BioGRID database are marked in green. **B** Co-immunoprecipitation of Flag-RFX7-HiBiT with overexpressed GFP-DYRK1A and GFP-DYRK1B from transiently transfected HEK293 cells. **C** A549 and A549 HiBiT-DYRK1B cells were grown to hyperconfluence [40] and treated with 10 µM Nutlin-3a for 24 h to induce maximal DYRK1B expression. Total cell lysates were subjected to immunoprecipitation (IP) with a DYRK1B reactive serum (+) or pre-immune serum as a negative control (–). Aliquots of the whole cell lysates are shown as input control. The blots in (**B**), (**C**) are representative of *n* = 3 independent experiments. **D** Flag-RFX7-HiBiT was co-expressed with GFP-DYRK1B (active p69 splicing variant). Control samples were transfected with empty vector or the catalytically inactive splicing variant DYRK1B-p65. Flag-RFX7-HiBiT immunoprecipitates were incubated for 2 h at 37 °C with Antarctic phosphatase (AP) or without (–). **E** Deletion constructs of Flag-RFX7-HiBiT. DBD denotes the DNA binding domain, PxLPxL is the sequence motif that mediates ANKRA2/RFXANK binding, and NLS marks the nuclear localization sequence. **F** RFX7 deletion constructs were co-expressed with GFP-DYRK1B-p69 as indicated, and total cellular lysates were analysed by Western blotting.

To further explore the molecular mechanism by which DYRK1B suppresses RFX7 transcriptional activity, we characterized RFX7-interacting proteins co-purified with immunoprecipitated Flag-RFX7 from HEK293 cells. Mass spectrometric analysis identified nine significantly enriched interactors (Fig. 7A). Among these, ANKRA2, RFXANK, and RFXAP were recently identified as core components of the functional RFX7 transcription initiation RFX7 complex [46]. ANKRA2 and RFXANK are known to directly bind to a PXLPXL motif in RFX7 [54]. Additional interactors include DYRK1A and DCAF7. DCAF7 is an adaptor protein for both DYRK1A and DYRK1B, acting as a substrate-recruiting subunit [55–57]. In silico modeling supported direct interactions of RFX7 with ANKRA2 and RFXANK and suggested further interactions between RFX7 and DYRK1A, DYRK1B, and DCAF7 (Supplementary Fig. S3).

DYRK1B itself was not detected in the interactomics experiment, likely due to its low basal expression in HEK293 cells. This finding is consistent with a recent total proteome analysis, which also failed to detect DYRK1B in HEK293 cells while readily identifying DYRK1A and DCAF7 [53]. However, overexpression experiments showed that not only DYRK1A but also DYRK1B can interact with RFX7 (Fig. 7B). Further co-immunoprecipitation experiments in A549 cells confirmed that endogenous DYRK1B associates with the RFX7 complex under physiological conditions (Fig. 7C).

The physical interaction between DYRK1B and RFX7 raises the question of whether RFX7 is a substrate of DYRK1B. Indeed, incubation with phosphatase reversed the DYRK1B-induced mobility shift of RFX7 (Fig. 7D). Analysis of RFX7 deletion constructs mapped the phosphorylation site(s) to the C-terminal region of RFX7, far distal to both the DNA-binding domain and the interaction sites for ANKRA2 and RFXANK (Fig. 7E, F). Collectively, these results indicate that DYRK1B physically interacts with and phosphorylates RFX7 within its C-terminal region, leading to slower electrophoretic migration and reduced transcriptional activity.

## Discussion

The tumor suppressor p53 regulates a wide range of target genes with divergent functions [2, 7, 12]. Surprisingly, protein kinases have received relatively little attention as downstream mediators of p53, with the notable exception of CDK inhibition by p21. In this study, we identified *DYRK1B* as a novel indirect target gene of p53, whose upregulation depends on the transcription factor RFX7. Additionally, we uncover a negative feedback loop in which DYRK1B suppresses RFX7 activity, revealing a new mechanism for fine-tuning p53-driven cellular responses.

The induction of DYRK1B expression upon treatment with the cytotoxic drugs Doxorubicin and Actinomycin D aligns with its known function as a promoter of cancer cell survival under stress conditions. Previous studies have demonstrated that DYRK1B levels increase in response to serum deprivation, hypoxia, high cell density, or mTOR inhibition [24, 40, 58, 59]. The identification of p53 as a regulator of DYRK1B expression integrates *DYRK1B* into the gene regulatory network through which p53 exerts its tumor suppressor functions. Notably, analysis of the TargetGeneRegulation Database [7, 47] revealed p53-dependent upregulation of DYRK1B not only in several human cancer lines but also in non-cancer cell lines and in murine embryonic fibroblasts. DYRK1A is phylogenetically and structurally closely related to DYRK1B and has overlapping functions in cell cycle regulation [14, 27, 36]. In contrast to *DYRK1B*, *DYRK1A* is not a p53 target gene and is not upregulated in cellular stress conditions [40]. Interestingly, *DYRK1B* expression is positively correlated with *TP53* mRNA levels in most tumor types, while the correlation between *TP53* and *DYRK1A* is negative (Fig. 2K).

Our results demonstrate that DYRK1B is regulated *via* p53-mediated activation of RFX7. The transcription factor RFX7 accounts for a significant part of the p53-induced changes in gene expression [13]. RFX7 controls several established tumor suppressor genes by direct interaction with their X-box promoter motifs [12, 45, 46]. Interestingly, murine *Dyrk1b* has previously been identified as a direct RFX7 target gene in natural killer lymphocytes [44]. In this report, RFX7 overexpression increased reporter gene activity driven by the *Dyrk1b* promoter region. Published ChIP-seq data document direct binding of RFX7 within 5 kb from the transcription start site of the *DYRK1B* gene in RPE-1 (retinal pigment epithelial) cells [13].

While p53 induces a modest increase in *RFX7* mRNA levels, RFX7 is primarily regulated through a reversible post-translational modification, as apparent by a change in its electrophoretic mobility [13]. The faster migrating form of RFX7, induced by p53 activation, is strongly associated with the transcriptional upregulation of RFX7 target genes [13, 45, 60]. Here, we discovered that DYRK1B counteracts p53-mediated RFX7 activation by upshifting the RFX7 band and inhibiting the induction of its target genes. This upshift depends on the catalytic activity of DYRK1B and was reversed by phosphatase treatment, suggesting that DYRK1B inactivates RFX7 through direct phosphorylation. Interestingly, we found that both DYRK1A and DYRK1B are associated with the RFX7 complex. Given that only DYRK1B is an RFX7 target gene, it can be speculated that the constitutive presence of DYRK1A maintains low basal activity of RFX7, while DYRK1B upregulation by RFX7 serves as a negative feedback mechanism to attenuate its activation.

RFX7 has recently been recognized as a tumor suppressor with recurrent mutations reported in hematopoietic malignancies [61]. In addition, RFX7 appears to be functionally silenced in many cancers in which it is not mutated, and reduced RFX7 expression is associated with unfavorable clinical outcomes across diverse tumor types [13]. Collectively, these findings underscore the rationale for therapeutic approaches that restore RFX7 function.

In our cell-based assays, pharmacological inhibition of DYRK1 converted RFX7 into its active form and increased levels of tumor suppressor protein PDCD4. Furthermore, DYRK1 depletion using the DYRK1-selective PROTAC DYR684 sensitized A549 cells to the cytotoxic effects of doxorubicin, and this response was partially dependent on the presence of RFX7. These findings suggest that reactivation of RFX7 contributes, at least in part, to the enhanced drug sensitivity observed upon DYRK1 depletion. It should be noted that DYRK1A and DYRK1B both interact with RFX7 and are both targeted by DYR684; however, only DYRK1B is induced by cytostatic drugs and is frequently elevated in human tumors. Thus, targeting DYRK1B with small-molecule inhibitors may represent a promising therapeutic strategy to restore RFX7 activity in cancer treatment.

In conclusion, this study identifies *DYRK1B* as an indirect p53 target gene that is induced through activation of RFX7 and functions as a negative feedback regulator of RFX7 transcriptional activity. Further investigation is required to elucidate the precise molecular mechanisms by which DYRK1B dampens RFX7 activity and to fully characterize the role of DYRK1B in the p53 gene regulatory network.

## Materials and methods

### Materials

Information about reagents and tools (cell lines, antibodies, plasmids, oligonucleotides, commercial kits and chemicals) is provided in the supplementary information.

### Cell culture and transient transfections

HEK293tsa201, PANC-1, HeLa, OVCAR3, MCF7, A549 cells and genetically-modified variants thereof were cultivated in DMEM/F12 or RPMI medium supplemented with 10% fetal bovine serum in a humidified atmosphere containing 5% CO_2_ at 37 °C. Specifications of media for each cell line, method of transient transfection, cell line authentication, mycoplasma testing, and Research Resource Identifiers (#RRID) are provided in the supplementary information. The generation of genetically modified cell lines is described in the supplementary information.

### HiBiT assays

Detection of soluble HiBiT-DYRK1B in cell lysates or immobilized on blotting membranes after SDS-PAGE was performed using commercial kits based on luciferase*-*based fragment complementation (Table S7 in the supplementary material).

### Cell lysis and Western blotting

Cells were washed with ice-cold PBS and lysed at 4 °C for 30 min in a non-denaturing lysis buffer (Table S4). After protein quantitation, equal amounts of protein were separated by SDS-PAGE and then transferred to nitrocellulose membranes for subsequent immunoblot analysis or HiBiT detection. Chemiluminescence signals were recorded with a CCD imager (Fujifilm LAS-3000). Bands were quantified using the Multi Gauge software (Fujifilm). Background signals were subtracted. To normalize results of independent replicate experiments, intensity values of the individual bands were normalized to the sum of the relevant data points from an individual experiment [62].

### Immunoprecipitation and phosphatase treatment

For co-immunoprecipitation experiments, Flag-RFX7-HiBiT and/or GFP-DYRK1A/B were transiently overexpressed in HEK293 tsa201 cells (3 µg Flag-RFX7-HiBiT per 10-cm dish for Fig. 7A; 1 µg Flag-RFX7 plus 100 ng of GFP-DYRK1A/B per 6-cm dish for Fig. 7B). Transient transfections were performed using FuGENE HD (Promega). Cells were lysed in IP buffer on ice. Bait proteins were isolated from cleared lysates by overnight incubation at 4 °C in an end-over-end rotator with appropriate affinity resins directed against the Flag tag or GFP (Table S7). Endogenous DYRK1B was immunoprecipitated using a custom-made antiserum directed against the C-terminus of DYRK1B [63]. For phosphatase treatment (Fig. 7D), lysates of two wells from a 6-well plate were combined, phosphatase inhibitors and EDTA were omitted from the lysis buffer, and the anti-Flag IP proceeded only for 2 h. After extensive washes, beads were transferred to fresh test tubes, resuspended in a volume of 50 µL, and incubated for 2 h at 37 °C with 20 U of Antarctic Phosphatase (NEB) in the dedicated Zn^2+^-containing buffer provided by the manufacturer.

### Cell proliferation and viability assay

A549-NT and A549-RFX7-KO cells (clones K1 and K12) were seeded in 96-well plates (9000 cells per well). After 24 h, cells were treated with Doxorubicin and DYR684 or DMSO control for 24 h. Subsequently, the cells were washed twice with PBS and recovered for 3 days in fresh media without Doxorubicin in the continuous presence of DYR684. WST-1 reagent (Sigma Aldrich) was added for 1 h before absorbance was measured at 450 nm.

### RNA-seq and RT-qPCR analysis

Commercial kits were used for RNA isolation, cDNA synthesis and RT-qPCR as specified in Table S7. RNA concentrations were determined photometrically (NanoDrop ND-1000, Peqlab). For RT-qPCR, reverse transcription was performed with 500 ng total RNA. Samples were diluted 1:10 before PCR reactions were run with three technical replicates, and a few erroneous data points were excluded as outliers. Primer sequences and thermocycling parameters are given in Table S8. Data were evaluated with the CFX Maestro Software 1.1 (Bio-Rad), and qPCR efficiencies were determined using the LinRegPCR program (version 2020.2) [64, 65]. RNA-seq analysis is described in the supplementary information.

### Proteomics and interactomics analyses

Methods for sample preparation, proteolytic digestion and analysis by mass spectrometry for whole proteome analysis of A549 cells and interactome analysis of RFX7-immunoprecipitates are detailed in the supplementary material.

### Statistics

If not stated otherwise, all data are presented as mean ± standard deviation (SD) and were calculated from at least three independent experiments. Exact sample sizes are indicated in each figure. Statistical analyses were performed using either Student’s *t* test or one-way/two-way ANOVA, depending on the experimental design. ANOVAs were conducted using the general mixed model procedure (PROC GLIMMIX, SAS 9.4, SAS Institute Inc., Cary, North Carolina, USA), while t-tests were performed using GraphPad Prism 9 (GraphPad Software, San Diego, California, USA). Two-way ANOVA for Fig. 6L was done using GraphPad Prism 9. All datasets were confirmed to be normally distributed based on residual analysis, the Shapiro-Wilk test, and model diagnostics, including the Akaike Information Criterion (AIC) and Bayesian Information Criterion (BIC). Variance homogeneity between groups was assessed using residual diagnostics, and in cases of heteroscedasticity, the Kenward–Roger approximation was applied to adjust degrees of freedom. All *p*-values were adjusted for multiple comparisons using the Bonferroni correction when appropriate. A *p*-value < 0.05 was considered statistically significant, with significant differences indicated by asterisks. In cases where the Shapiro-Wilk test indicated a significant deviation from normality (Fig. 3E), a nonparametric Scheirer-Ray-Hare test was performed, followed by Dunn’s post hoc test with Bonferroni correction. These analyses were conducted in R (version 4.5.0) using the rcompanion and FSA packages. Statistical analyses related to the RNA-seq and proteomics data are described in the respective sections of the supplementary information.

### In silico expression analysis

Expression changes of *DYRK1A* and *DYRK1B* between tumor and normal tissues were analyzed using the pan-cancer box plot tool of the TNMplot web application (accessed 2024/07/09) [39]. Spearman correlation analyses between *TP53* and *DYRK1*A or *DYRK1B* mRNA were performed using the implemented gene vs. gene correlation tool based on gene chip data from tumor tissues. Log2FC values of p53-dependent regulated genes upon MDM2 inhibition were obtained from the TargetGeneRegulation Database 2.0 (www.targetgenereg.org; accessed 2024/07/02) [47]. Data from genetically modified cell lines (HCT116_ATF3_KO, HCT116_p21_KO, and HCT116_LIN37_KO) were excluded from this analysis.

### In silico interaction modeling

Binary protein-protein interactions of RFX7 were modeled using AlphaFold Multimer [66] as described in the supplementary information.

## Supplementary information

Supplementary information

Uncropped Western blots

RNAseq_Readme

RNAseq_DEG

RNAseq_ raw read counts

A549_control_vs_DYRK1Boe.xlsx

## Data Availability

The mass spectrometry proteomics data have been deposited to the ProteomeXchange Consortium (http://proteomecentral.proteomexchange.org) via the PRIDE partner repository with the data set identifier PXD067702. The RNA-seq datasets analyzed in this study are provided as supplementary information. Publicly available data used in this study originated from TNMplot (https://tnmplot.com/analysis/) and TargetGeneReg 2.0 web atlas (http://www.targetgenereg.org/). All other data generated and/or analyzed during the current study are included in this published article and its supplementary information files.
