# Supplementary material for: The protein kinase DYRK1B is a p53 target gene and functions as a negative feedback regulator of the transcription factor RFX7
Source: Cell Death Dis. 2026 Mar 26;17(1):386. doi: 10.1038/s41419-026-08660-x (PMC13066115; doi:10.1038/s41419-026-08660-x)
Supplement: Supplementary file 1 — Supplementary information [file 41419_2026_8660_MOESM1_ESM.pdf]

Supplementary information to

Gerrit Wilms, Katharina Schwandt, Stefan Düsterhöft, Philip Helmich, Justyna Wozniak, Florian Kraft, Sebastian Kallabis, Felix Meissner, Walter Becker

The protein kinase DYRK1B is a p53 target gene and functions as a negative feedback regulator of the transcription factor RFX7

Contents

		Page
1.	**Supplementary Results**	
	Figure S1: Differential expression of *DYRK1A* and *DYRK1B* in human tumors	2
	Figure S2: Gene set enrichment analyses of RFX7 targets	3
	Figure S3: Protein interaction heatmap of RFX7-associated proteins	4
	Table S1: Correlation between *TP53* and *DYRK1* expression in different tumor tissues	5
	Table S2: Changes of select mRNAs in response to MDM2 inhibition	6
2.	**Supplementary Materials**	
	Table S3: Cell lines	7
	Table S4: Experimental conditions for cell culture experiments	8
	Table S5: Plasmids	9
	Table S6: Antibodies	10
	Table S7: Chemicals and commercial kits	11
	Table S8: Oligonucleotide primers for RT-qPCR	12
**3.**	**Supplementary Methods**	
	Generation of genetically modified cell lines	13
	Vector construction: DYRK1B pN-PITCh-HiBiT	13
	Vector construction: FUW-tetON-GFP-2A-hDYRK1B and mRFX7	14
	Vector construction: lentiCRISPR v2 RFX7	15
	Lentivirus production and transduction	16
	RNA-seq	16
	Proteomics	16
	Interactomics	18
	Modeling of binary protein-protein interactions	19
**4.**	**References**	19

1. Supplementary Results

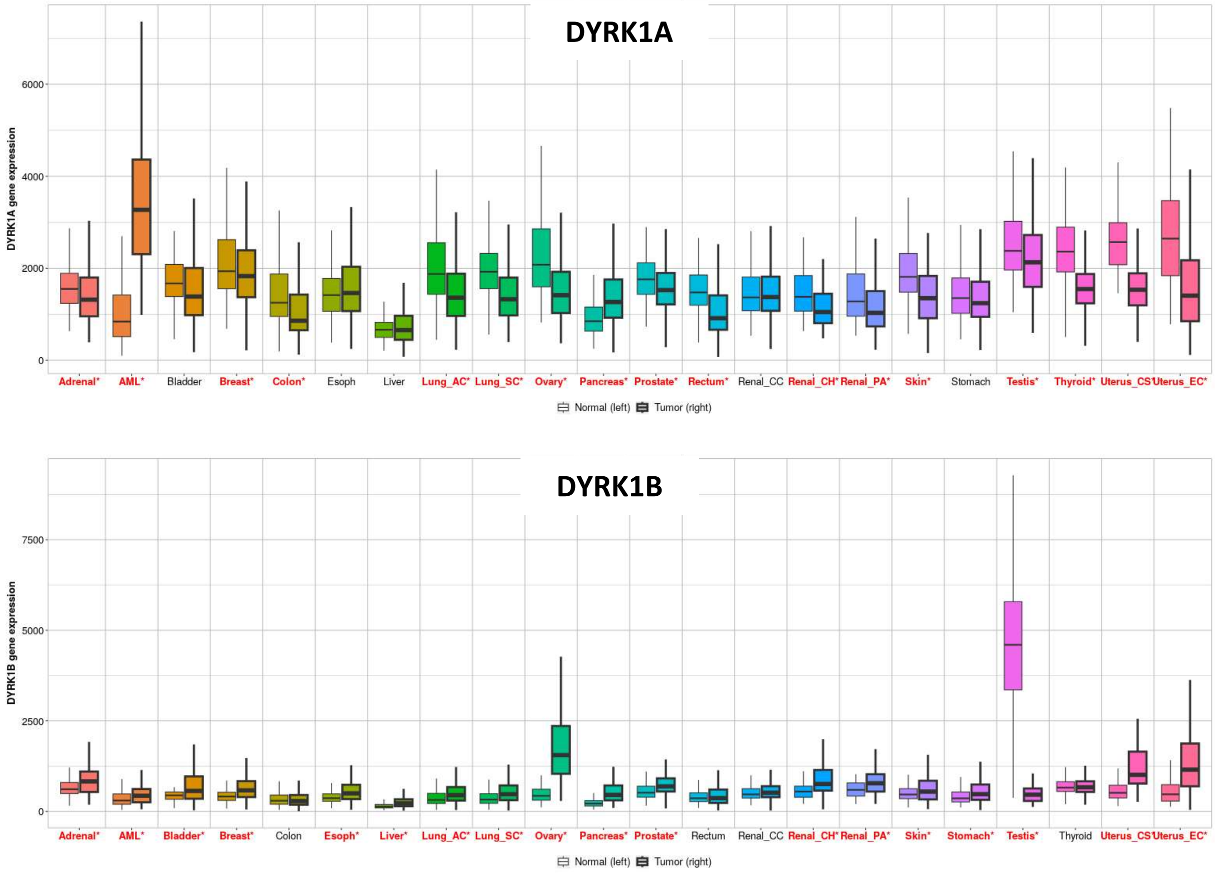

Figure S1: Differential expression of *DYRK1A* and *DYRK1B* in human tumors.

Box plots illustrate the differential expression of *DYRK1A* (top panel) and *DYRK1B* (lower panel) in unpaired samples of 22 types tumor types (bold, right boxes) compared to healthy tissues (thin, left boxes). The analysis was conducted using the TNMplot database based on RNA expression data from 56,938 unique human tissue samples (Bartha et al. 2021). Tissue with significantly different (Mann-Whitney test $p < 0.05$) mRNA levels between normal and tumor tissue are marked in red. *DYRK1B* is known to be highly expressed in normal testis (Leder et al. 1999).

List of tissues (alphabetically from left to right):
Adrenal gland, Acute myeloid leukemia (AML), Bladder, Breast, Colon, Esophagus, Liver, Lung adenocarcinoma, Lung small cell carcinoma, Ovary, Pancreas, Prostate, Rectum, Renal cell carcinoma, Chromophobe renal cell carcinoma, Renal papillary adenoma, Skin, Stomach, Testis, Thyroid, Uterus cervix, Uterus endometrium

A

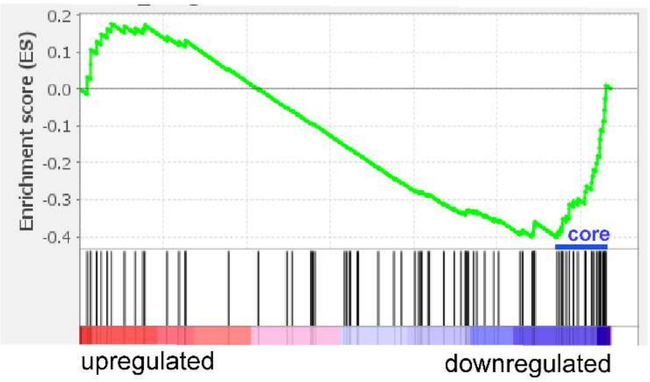

RFX7 target genes

Coronel et al. 2021				Schwab et al. 2023	
ABAT	EMC9	PI4K2A	SOX12	ARRDC3	PINLYP
ARL15	FAM111A	PIK3IP1	SOX4	CCND2	PMEPA1
ATRX	FAM214A	PIK3R3	TOB1	CLIC4	PNKP
CABIN1	HNRNPUL2	PLCXD2	TOB2	CNOT1	PNRC2
CAT	INTS3	PNRC1	TOP2B	COQ10A	PPP2R5D
CCND1	IP6K2	PRKCZ	TP53INP1	DCP2	PPP3CA
CCNG2	JUNB	PTMS	TSC22D1	FAM111A-DT	PRCD
CDK4	KDM4A	RAB40B	TSPYL1	FLJ20021	RIBC1
CIC	KDM6B	RAP2A	TSPYL2	IRF2BP2	SLFN12
CKAP2	KLF9	REV3L	UBE2H	JUN	SYNPO
CKS2	MAF	RFX5	XRCC1	MAFTRR	TOB1-AS1
DDIT4	MXD4	RMND5A	YPEL2	MARK3	TUBA1A
DIP2A	NRSN2-AS1	RPS6KA5	AMN1	MIR22HG	UCP2
DOLPP1	OTUD5	SESN3	APC2	N4BP2	ZFAND4
DSE	PDCD4	SLC43A2	ARNTL	PDCD4-AS1	ZNF791

B

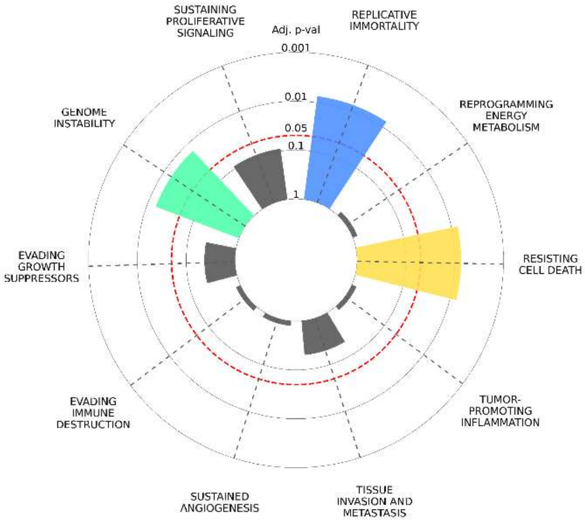

Figure S2: Gene set enrichment analyses of RFX7 target genes

A, Downregulation of RFX7 target genes in DYRK1B-overexpressing A549 cells. The GSEA 4.4.0 software (www.gsea-msigdb.org; Subramanian et al. 2005) was used to assess the enrichment of the listed RFX7 target genes in A549 tetON DYRK1B^{WT} cells. DYRK1B overexpression was induced by treatment doxycycline and proceeded for 7 days before RNA was isolated and subjected to RNA-seq analysis. Expression of RFX7 target genes was significantly downregulated DYRK1B overexpressing cells (normalized enrichment score (NES) = -1.56; FDR q-value = 0.005. Genes contributing to the core set of DYRK1B-downregulated genes are highlighted in blue.

B, Cancer hallmark enrichment of RFX7 target genes**.** The RFX7 target genes identified in the proteomic experiment (Fig. 6B, C, D) were analysed for enrichment of cancer hallmark genes using the online tool developed by Menyhart et al. (2025) (www.cancerhallmarks.com). The plot shows the enrichment of the input gene list for the 10 cancer hallmark categories as defined in the core cancer hallmark gene-set defined by Menyhart et al. (2025). The colored slices represent those hallmarks for which the over-representation test yielded an adjusted *p*-value < 0.05 (red dotted threshold line shown). The size (angle) of each slice corresponds to the number of genes from the input list mapped to that hallmark, and the radial distance reflects the enrichment score.

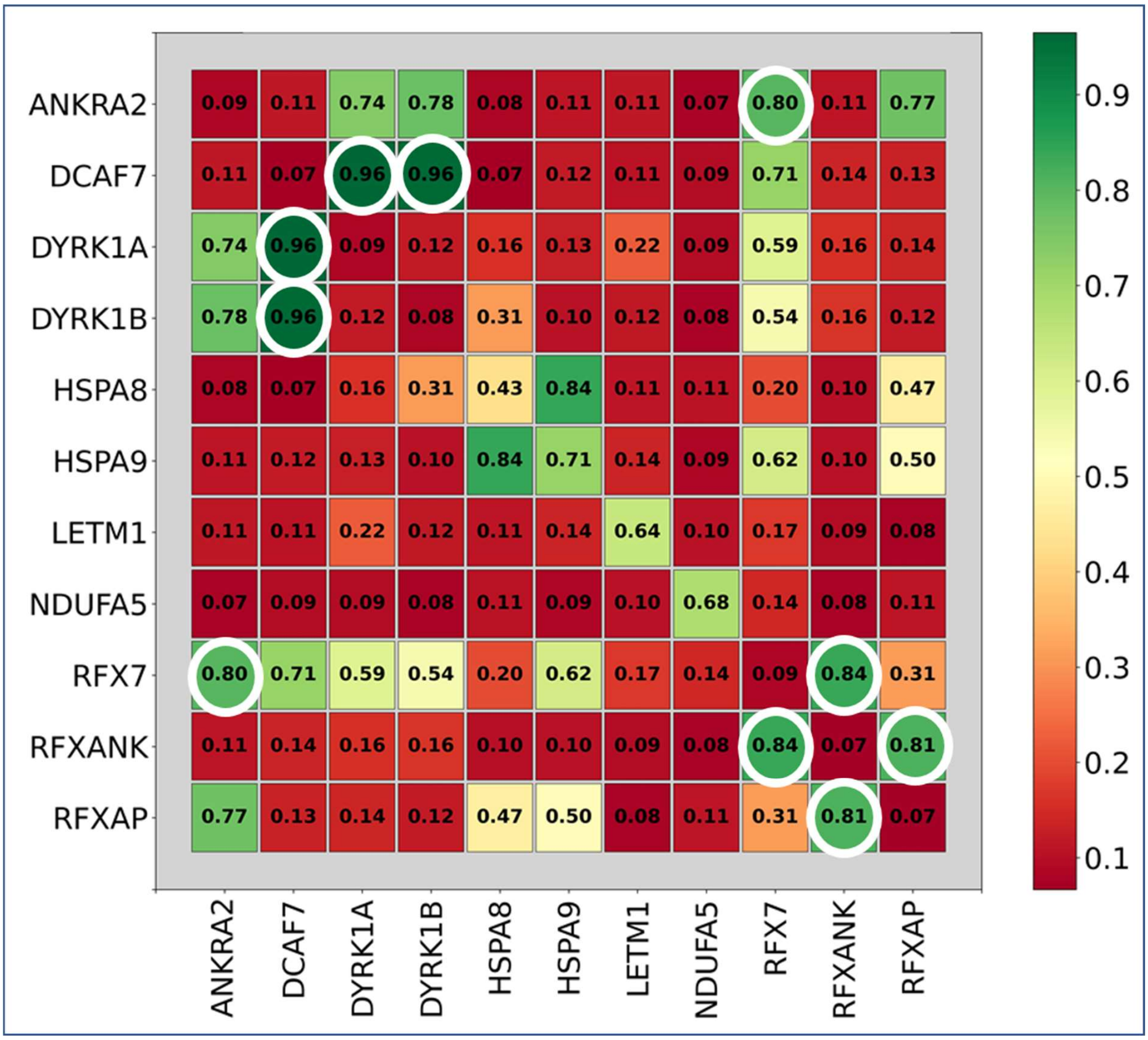

Fig. S3: Protein interaction heatmap of RFX7-associated proteins.

Binary protein-protein interactions of RFX7 and co-immunoprecipitated proteins as well as DYRK1B were modeled using AlphaFold Multimer. Each AlphaFold prediction produces five different models, which are predicted using five sets of parameter weights. As high consensus among the five models implies high reliability, we calculated the average interaction confidence score. High scores above 0.75 indicate high confidence interaction, while scores above 0.5 still indicate moderate confidence. Modeling results are validated by the high scores obtained for known binary interactions (circled, data taken from "binary interactions" in the UniProt knowledgebase). DCAF7, DYRK1A, DYRK1B and HSPA9 show moderate confidence interactions with RFX7. No direct interactions were detected between LETM1 and NDUFA5 with any of the other proteins. This suggests that these proteins are false positives or that AlphaFold Multimer did not capture their interaction, e.g., because these interactions are driven by post-translational modifications.

Table S1: Correlation between *TP53* and *DYRK1* expression in different tumor tissues.

The table supports Fig. 2K in the main text and summarizes the relationships between the co-expression of *TP53* and *DYRK1A* (left) or *DYRK1B* (right) based on gene chip data from 30 different tumor types. Correlation analyses were conducted using TNMplot.com (accessed on August 14, 2024) (Bartha et al. 2021). The table presents the sample size (N), p-values, and correlation coefficients (R) determined by Spearman correlation. Positive correlation coefficients are highlighted in green, negatives in red. Non-significant correlation ($p > 0.05$) are marked by shading. Results for lung tumors, labelled with *, are shown as scatter plots in Figure 2J of the main text.

		DYRK1A		DYRK1B	
**Tissue**	**N**	**R**	**p**	**R**	**p**
Adrenal Gland	61	0.24	0.06	0.17	0.19
Bladder	144	0.01	0.91	0.15	0.07
Breast	7569	-0.22	0.00	0.23	0.00
Cervix	189	-0.43	0.00	0.36	0.00
CNS	2159	-0.54	0.00	0.23	0.00
Colon	1450	-0.37	0.00	0.27	0.00
Endometrium	39	-0.20	0.22	0.35	0.03
Gastric	1221	-0.29	0.00	0.41	0.00
Head and neck	99	0.73	0.00	0.76	0.00
Intestine	10	-0.41	0.24	-0.37	0.29
Kidney	556	-0.35	0.00	0.60	0.00
Liver	806	0.03	0.35	0.17	0.00
***Lung**	**1865**	**-0.50**	**0.00**	**0.40**	**0.00**
Lymphoid	5539	-0.33	0.00	0.26	0.00
Myeloid	3570	-0.33	0.00	0.32	0.00
Nasopharyngeal	56	-0.28	0.04	0.45	0.00
Neural	245	-0.25	0.00	0.23	0.00
Oesophageal	440	-0.24	0.00	0.26	0.00
Oral cavity	38	-0.53	0.00	-0.24	0.14
Ovarian	744	-0.28	0.00	0.33	0.00
Pancreas	248	-0.27	0.00	0.38	0.00
Pituitary	18	-0.42	0.08	0.34	0.17
Prostate	283	0.03	0.65	0.10	0.09
Salivary gland	10	0.42	0.23	0.27	0.45
Skin	253	0.48	0.00	0.02	0.69
Soft tissue	1017	-0.19	0.00	0.19	0.00
Thyroid	215	-0.10	0.13	0.34	0.00
Tongue	57	0.18	0.17	0.23	0.09
Uterus	154	-0.65	0.00	0.33	0.00
Vulva	15	-0.61	0.02	0.34	0.21

.

Table S2: Changes of select mRNAs in response to MDM2 inhibition

Tabular summary of Log2 fold changes and adjusted p-values of select mRNAs as determined by RNA-seq analysis of A549^{NT} cells treated with 10 µM Nutlin-3a (n=4) or DMSO (n=3) for 24 h. This table supports Fig. 4L & M. *MDM2*, *CDKN1A* and *RFX7* represent direct p53 target genes, while *PDCD4* is an RFX7 target.

Gene	Log2FC	Adj. p-value
MDM2	4.886	2.1212e-10
CDKN1A	5.576	2.1212e-10
DYRK1A	-0.160	0.2158
DYRK1B	1.436	0.0003
DYRK2	-0.102	0.5188
DYRK3	0.554	0.0086
DYRK4	-0.112	0.4084
RFX7	1.118	2.7399e-05
PDCD4	1.176	3.0344e-06

2. Supplementary Materials

Table S3. Cell lines

Human cell line	Description	Provided by	RRID Identifier	Authentication
HEK tsA201	Transformed cell line (fetal kidney)	Sigma-Aldrich # 96121229	RRID:CVCL_2737	(provider)
A549 (Abcam)	Lung adenocarcinoma	Abcam Cat# (ab275463)	not available	(provider)
A549 p53-KO[c]	Lung adenocarcinoma	Abcam Cat# ab276092	not available	(provider)
A549 and genetically modified derivatives[d]	Lung adenocarcinoma	Available in the lab	RRID:CVCL_0023	MCA[a]
HeLa	Cervical adenocarcinoma	Available in the lab	RRID:CVCL_0030	STR profiling[b]
MCF7	Breast carcinoma	Kind gift of Rudolf Leube*	RRID:CVCL_0031	MCA[a]
OVCAR-3	Ovarian serous adenocarcinoma	CLS Cell Line Services GmbH	RRID:CVCL_0465	(provider)
PANC-1	Pancreatic ductal adenocarcinoma	Kind gift of Edgar Dahl**	RRID:CVCL_0480	MCA[a]

[a] Multiplex human Cell line Authentication Test (MCA) by Multiplexion GmbH, Friedrichshafen, Germany
[b] the used HeLa cells deviate in one marker from CVCL_0030 (Multiplexion GmbH, Friedrichshafen, Germany)
[c] homozygous knockout achieved by using CRISPR/Cas9 (49 bp deletion in exon 4)
[d] Generation of genetically modified A549 cell lines (HiBiT-DYRK1B, RFX7-KO, tetON-RFX7) is described in this paper. A549 tetON-DYRK1B cells were reported previously (Sester et al. 2024).
* Institute of Molecular and Cellular Anatomy, RWTH Aachen University, Aachen, Germany
** Institute of Pathology, RWTH Aachen University, 52074 Aachen, Germany

Table S4. Experimental conditions for cell culture experiments

Cell line	Cells plated	Figure	Type of plate[a]	Lysis buffer[b]	Lysis volume (µl)	BCA Assay[d]
A549	200,000	1A-D	6-well	Native	110-130	Kit A
HeLa	250,000	1 E	6 well	Native	110-130	Kit A
MCF7	300,000	1F	6 well	native	140	Kit A
A549 and A549-HiBiT-DYRK1B	1,250,000	1H, 7C	10-cm dish	IP LP	1000	n.d.
A549	200,000	2A-B	6 well	Native	110-130	Kit A
A549-HiBiT-DYRK1B	150,000	1I-L 2C-D	6 well	Native	150	Kit A
PANC-1	250,000	2H	6 well	Native	110-130	Kit A
OVCAR3	250,000	2I	6 well	Native	110-130	Kit A
A549 WT (Abcam) and A549 p53-KO	200,000	2E-G	6 well	Native	110-130	Kit A
A549 NT and A549 RFX7-KO	200,000	3B-E 5A,	6 well	Native	110-130	Kit A
A549 tetON mRFX7^{WT} and mRFX7$^{\Delta NLS}$	200,000	3F	6 well	Native	110-130	Kit A
A549 A549 p53-KO A549 NT and A549 RFX7-KO	200,000	4A-M	6 well	RLT	350	
A549	200,000	5B	6 well	Native	110-130	n.d.
A549 tetON DYRK1B^{WT} and DYRK1B^{Y273F}	200,000	5C-D	6 well	Native	110-130	Kit A
A549 tetON DYRK1B^{WT}	2,000,000	6 (A-D)	10-cm dish	GuHCl	250	Kit B
HEK tsA201	500,000	7B	6-cm dish	IP LP	500	n.d.
HEK tsA201	250,000	7D	6 well	IP LP[c]	250	n.d.
HEK tsA 201	1.000.000	7A	10-cm dish (Cell+)	IP LP	700	Kit B

[a] Standard 6-well cell culture plates (Sarstedt #833.920.005) or 10-cm dishes (Sarstedt, #833.902) were used throughout except for HEK tsa201 cells, which were grown on plates with Cell+ surface (Sarstedt 83.3901.300 and 83.3902.300).

[b] Native lysis buffer (for Western blot analysis): 20 mM HEPES pH 7.4, 150 mM NaCl, 2 mM EDTA and 1% Igepal CA 630 + (1 mM Na_3VO_4, 1 mM PMSF, 10 µg/ml aprotinin,4 µg/ml pepstatin and 16 µg/ml leupeptin)
IP lysis buffer (for immunoprecipitation): 20 mM HEPES pH 7.4, 150 mM NaCl, 2 mM EDTA and 0.5% Igepal CA 630 + (1 mM Na3VO4, 1 mM PMSF, 10 µg/ml aprotinin,4 µg/ml pepstatin and 16 µg/ml leupeptin, 1 mM NaF)
RLT buffer (for RNA isolation) provided in the RNeasy kit (Qiagen)
GuHCl (for proteomics analysis): 6 M GuHCL, 5 mM Bond-Breaker TCEP solution, 10 mM 2-Chloracetamid, 100 mM Tris-HCl (pH 8.5)

[c] phosphatase inhibitors (EDTA, Na3VO4, NaF) were omitted in dephosphorylation experiments (Fig. 7D)

[d], protein concentrations were determined using Pierce BCA Protein Assay Kit A (Thermo Scientific Cat.-No. 23225) and adjusted to ensure equal loading in SDS PAGE or using Kit B (Cat.-No. 22660) to in proteomics experiments..

Table S5: Plasmids

Plasmid	cDNA / insert	Vector	Source / Reference
pN-PITCh-HF	GFP-2A-Puro-2A-HF	pN-PITCh	Addgene (#127882)
pX330-BbsI-PITCh	Cas9	pX330-BbsI-PITCh	Addgene (#127875)
FUW-tetON-hMyc	hMYC	FUW-tetON	Addgene (#20723)
FUW-M2rtTA	rTetR	FUW-M2rtTA	Addgene (#20342)
lentiCRISP v2		lentiCRISP v2	Addgene (#52961)
lentiCRISP v2-NT	NT gRNA	lentiCRISP v2	Athanassios Fragoulis (RWTH Aachen)
lentiCRISP v2-RFX7	RFX7 gRNA	lentiCRISP v2	This paper
pEGFP-DYRK1A	GFP-DYRK1A	pEGFP-C1	Becker et al. 1998
pEGFP-DYRK1B-p69	GFP-DYRK1B-p69	pEGFP-C1	Leder et al. 1999
pEGFP-DYRK1B-p65	GFP-DYRK1B-p65	pEGFP-C1	Leder et al. 1999
FUW-tetON-hDYRK1B-WT	GFP-2A-hDYRK1B-WT	FUW-tetON	Sester et al. 2024
FUW-tetON-hDYRK1B-Y273F	GFP-2A-hDYRK1B-Y273F	FUW-tetON	Sester et al. 2024
FUW-tetON-mRFX7-WT	GFP-2A- mRFX7-WT	FUW-tetON	This paper
FUW-tetON-mRFX7-ΔNLS	GFP-2A- mRFX7-ΔNLS	FUW-tetON	This paper
pN-PITCh-HiBiT	GFP-2A-Puro^R-2A-HiBiT	pN-PITCh	This paper
pN-PITCh-HiBiT MH-DYRK1B-p69	MH1-GFP-2A-Puro^R-2A-HiBiT-MH2	pN-PITCh	This paper
pX330-BbsI-PITCh DYRK1B-p69	DYRK1B-p69 gRNA	pX330-BbsI-PITCh	This paper
pcDNA5/FRT/TO Flag-HiBiT		pcDNA5/FRT/TO	Papenfuss et al. 2022
pcDNA5/FRT/TO Flag-mRFX7-HiBiT and deletion constructs	Flag-mRFX7-HiBiT	pcDNA5/FRT/TO	This paper
pMD2.G	VSV-G	pMD2.G	Addgene (#12259)
psPAX2	HIV-1 gag pol	psPAX2	Addgene (#12260)

Table S6. Primary and secondary antibodies

Antibody target	Provider	Source/Clone	Dilution	Cat.-No.	RRID*
DYRK1A	Santa Cruz Biotechnology	Mouse mAb RR.7	1:200	sc-100376	RRID:AB_1122375
DYRK1B	Cell Signaling Technology	Rabbit polyclonal	1:1000	#2703	RRID:AB_2261790
DYRK1B	custom made (Leder et al. 2003)	Rabbit polyclonal	1:500 (for IP)	n.a.	n.a.
p53 (TP53)	Cell Signaling Technology	Mouse mAb DO-7	1:1000	#48818	RRID:AB_2713958
p21 (CDKN1A)	Cell Signaling Technology	Mouse mAb DCS60	1:1000	#2946	RRID:AB_2260325
RFX7	Bethyl Laboratories	Rabbit polyclonal	1:1000	# A303-062A	RRID:AB_10892718
PDCD4	Cell Signaling Technology	Rabbit mAb D29C6	1:1000	#9535	RRID:AB_2162318
GAPDH	Cell Signaling Technology	Rabbit mAb 14C10	1:1000	#2118	RRID:AB_561053
GFP	Rockland	Goat polyclonal	1:1000	600-101-215	RRID:AB_218182
β tubulin	Cell Signaling Technology	Mouse mAb D3U1W	1:1000	#86298	RRID:AB_2715541
Actin	Sigma-Aldrich	Rabbit polyclonal	1:1000	A2066	RRID:AB_476693
Phospho-Histone H2A.X (Ser139)	Cell Signaling Technology	Rabbit polyclonal	1:1000	#2577	RRID:AB_2118010
Rabbit IgG (H+L)	Rockland	Donkey (Secondary)	1:2000	611-703-127	RRID:AB_218614
Mouse IgG	Rockland	Donkey (Secondary)	1:2000	610-703-124	RRID:AB_218548
Mouse IgG	Invitrogen (Thermo Scientific)	Goat (Secondary)	1:2000	# 31430	RRID:AB_228307
Goat IgG(H+L)	Rockland	Donkey (secondary)	1:2000	605-703-125	RRID:AB_218291
IgG subclasses (Clean-Blot IP Detection Reagent)	Thermo Fisher Scientific	Undisclosed by provider	1:200	#21230	RRID:AB_2864363

* Research Resource Identification Portal (https://scicrunch.org/resources)

Table S7. Chemicals and commercial kits

Chemical or Kit	Provider	Cat.-No.
Actinomycin D	Selleckchem (Houston, TX, USA)	S8964
AnnH31	available in the lab (Rüben et al 2015)	
AZ191	Sigma-Aldrich (St. Louis, MO, USA)	SML1089
Doxorubicin	MedChemExpress (Monmouth Junction, NJ, USA)	HY-15142
Doxycyclin	AppliChem (Darmstadt, Deutschland)	A2951
DYR684	available in the lab (Wilms et al. 2024)	n.a.
Nutlin-3a	MedChemExpress	HY-10029
Puromycin	Calbiochem (San Diego, CA, USA)	540411
DMEM/F12, Hepes (Gibco)	Thermo Fisher Scientific (Waltham, MA, USA)	D8537
RPMI 1640	PAN Biotech (Aidenbach, Germany)	P04-18047
Fetal Bovine Serum (Gibco A5256801)	Thermo Fisher Scientific	17593595
FuGENE® HD Transfection Reagent	Promega (Madison, WI, USA)	E2312
NEBuilder HiFi DNA Assembly Master Mix	NEB (Ipswich, MA, USA)	E2621L
Cell line Nucleofector kit T	Lonza	VACA-1002
Q5 High-Fidelity DNA Polymerase	NEB (Ipswich, MA, USA)	M0491S
Pierce BCA Protein Assay Kit	Thermo Fisher Scientific	#23225
Antarctic Phosphatase	NEB	M0289S
Antarctic Phosphatase Buffer	NEB	B0289S
RNeasy Mini Kit (for RNA extraction)	Qiagen (Hilden, Germany)	#74104
QIAshredder Kit	Qiagen	#79656
QIAGEN Plasmid Maxi Kit	Qiagen	#12163
GeneJET Plasmid-Miniprep Kit	Thermo Fisher Scientific	K0503
ChromoTek GFP-Trap™ Magnetic Agarose	Proteintech	gtma-100
EZview™ Red Protein A Affinity Gel	Sigma-Aldrich	#P6486
anti-FLAG M2 magnetic beads	Sigma-Aldrich	A2220
NucleoBond Xtra Midi Plus Kit	Macheray & Nagel (Düren, Germany)	740412.50
PrimeScript RT Master Mix	Takara/Clontech (Saint-Germain-en-Laye, France	#RR036A
LightCycler 480 SYBR Green I Master	Roche (Penzberg, Germany)	04707516001
Nano-Glo HiBiT Blotting System	Promega	#N2410
Nano-Glo HiBiT Lytic Detection System	Promega	#N3040
Quick Cell Proliferation Assay Kit (WST-1 based)	BioCat (Heidelberg, Germany)	K301-500-BV
WesternFroxx all-in-one Proteinmarker (15 – 200 kDa)	neoFROXX (Einhausen, Germany)	# 7777YL250

Table S8. Oligonucleotide primers for RT-qPCR

PCR products were analyzed by agarose gel electrophoresis to verify the correct amplicon size and to check for the presence of non-specific products.

Gene symbol	Primer sequence (forward)	Primer sequence (reverse)	Annealing temperature*
DYRK1A	TCTGGGTATTCCACCTGCTC	GTCCTCCTGTTTCCACTCCA	63°C
DYRK1B	GATCTACCAGTATATCCAGAGCC	CCCTGGTAATCCTTCCTGAG	59°C
CDKN1A	AGGTGGACCTGGAGACTCTCAG	TCCTCTTGGAGAAGATCAGCCG	60°C
RFX7	CATGAAGGCACGTCGTTTGG	ACGGTGTCAAATGGTTGGCT	58°C
*GAPDH***	CGGGGCTCTCCAGAACATCATCC	CCAGCCCCAGCGTCAAAGGTG	66°C
*TBP***	GAGCCAAGAGTGAAGAACAGTC	GCTCCCCACCATATTCTGAATCT	60°C

* Cycling program: Initial denaturation 5 min 95°C; 40 cycles 10 s 95°C, 20 s annealing; 20sec 72°C; melting curve 65-95°C with 0.5°C increment per 5 s.

** GAPDH and TBP were identified as stable reference genes for treatments with doxorubicin, actinomycin D, and Nutlin-3a using the 'Reference Gene Selection Tool' in CFX Maestro Software 1.1 (Bio-Rad, Hercules, USA).

Table S9: Oligonucleotides used for the construction of the DYRK1B HiBiT knock-in constructs

Designation in Fig. S3	Sequenz 5´-> 3´	comment
a	CACCGGTGGGCTCAGAGGGCCGCAG	Insertion of gRNA into BbsI site
b	AAACCTGCGGCCCTCTGAGCCCACC	
c	TAGCGGATCCTCGGGTCCAAACACGTACGCGTACG	amplification of backbone
d	GCCAGCCGCTCACCATAGGTCCAGGGTTCTCC	
e	GGACCTATGGTGAGCGGCTGGCGGCTGTTCAAGAAGA TTAGCGGATCCTCGGGTCCAAACACG	template for HiBiT sequence
f	TGCTATGTAACGCGGAACTCCATATATGGG	amplification of backbone
g	CCAAACACGTACGCGTACGATGCTCTAGAATG	
h	GCATCGTACGCGTACGTGTTTGGCCATGGCCCGGTGGG ACGGCACCCGAGGATCCGCTAATCTTCTTGAA	changing microhomologies
i	CCGCGTTACATAGCATCGTACGCGTACGTGTTTGGCTCC AGGCCTCGCTCCCCTGGCCACCATGGTGAGCAAGGG	

3. Supplementary Methods

Generation of genetically modified cell lines

The generation of A549-tetON DYRK1B-WT and Y273F cell pools by lentiviral co-transduction of the FUW-tetON expression vector with FUW-M2rtTA, which encodes the tetracycline-controlled transactivator protein (gift from Rudolf Jänisch, Addgene #20342), was described previously (Sester et al. 2024). The same vector system was used to establish A549 cells with stable and inducible expression of murine RFX7. cDNA clones for wild type RFX7 or the mutant version (ΔNLS) were kindly provided by Greta Guarda (Castro et al. 2018). Positive cell clones were identified by autofluorescence of co-expressed GFP. Experiments were performed with pools that were compiled from 8-9 GFP^+ cell clones. Transgene expression was induced with doxycycline at a final concentration of 2 µg/ml.

TP53 (p53) knockout A549 cells and the parental A549 TP53WT cell line were purchased from Abcam (Cambridge, UK). A549–RFX7 knockout cells were generated with the help of the lentiCRISPR v2 vector (gift from Feng Zhang, Addgene #52961). Positive cells were selected with puromycin (0.75 µg/ml) and used without isolation of cell clones except for the chemoresistance assays (Fig. 6L, K), for which cell clones were isolated and expanded. For CRISPR/Cas9-based N-terminal HiBiT tagging of endogenous DYRK1B, we utilized the plasmid system developed by Lin and coworkers (2019) (gift of Peter Kaiser, Addgene #127875, #127882). Plasmids were co-transfected into A549 cells using the Amaxa Nucleofector II with 82 µl Nucleofector® Solution T + 18 µl Supplement 1 and program X-001 (Lonza, Basel, Switzerland).

Vector construction: DYRK1B pN-PITCh-HiBiT

N-terminal HiBiT tagging of endogenous DYRK1B was achieved using the two-plasmid-based PITCh (precise integration into target chromosome) system as described by Lin and co-workers (2019). A suitable N-terminal gRNA target site close to the translation start site of the DYRK1B-p69 splice variant was identified using the sgRNA designer tool from the Broad Institute (https://portals.broadinstitute.org/gpp/public/, Doench et al. 2016, Sanson et al. 2018). The cloning strategy is described in Fig. S3.

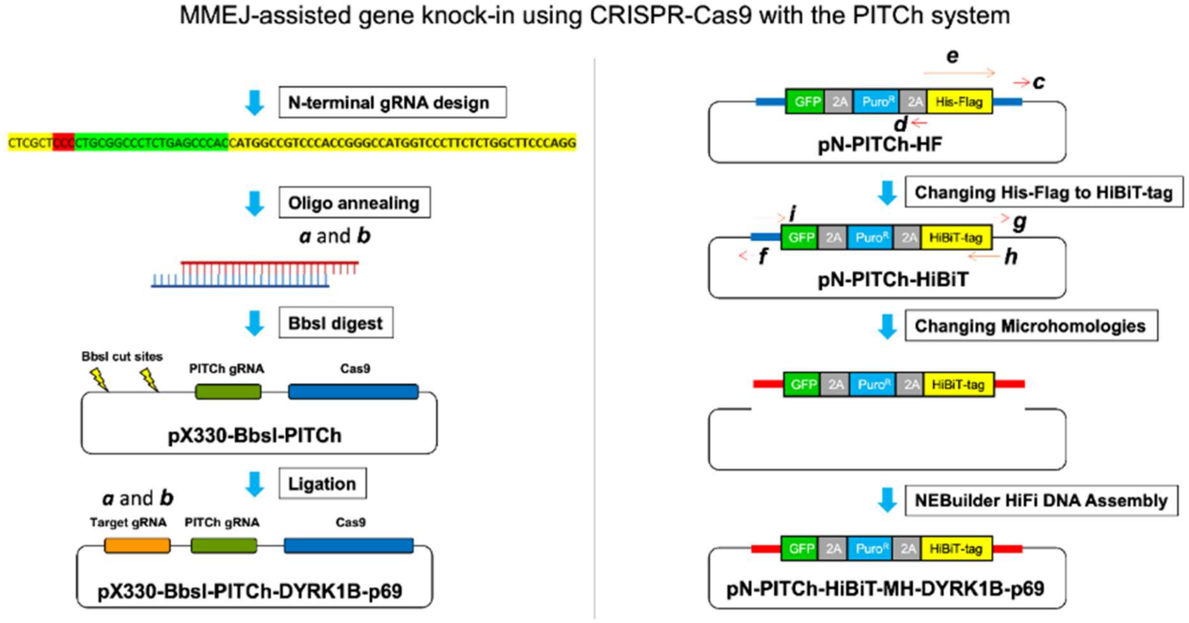

Fig. S4: Cloning strategy to generate pX330-BbsI-PITCh and pN-PITCh-HiBiT

To integrate the DYRK1B gRNA into the pX330-BbsI-PITCh vector, complementary oligonucleotides with appropriate overhangs were designed (see Table S9). Single-stranded oligonucleotides were phosphorylated, annealed and ligated into the *Bbs*I-linearized vector to produce pX330-BbsI-PITCh-DYRK1B-p69.

The second plasmid is designed to release the repair fragment that contains the tags flanked by microhomologies to the gRNA target site. pN-PITCh-HiBiT was generated by replacing the original 6xHis+3xFlag tag in the pN-PITCh-HF plasmid by the HiBiT tag. To this end, the HiBiT sequence was inserted into the linearized vector as a single oligonucleotide (***e***) using the NEBuilder HiFi DNA Assembly Cloning Kit. The DYRK1B-specific microhomology (MH) sequences targeting the genomic Cas9 cleavage site were introduced through the design of the PCR primer sequences (primers ***f-i***) that were used to amplify the GFP-PuroR-HiBiT-repair cassette and the vector backbone for NEBuilder assembly. In this process, the *Bam*HI restriction site located upstream of GFP was removed and replaced by a Kozak sequence.

The gRNA target site in the *DYRK1B* sequence is highlighted in green and the protospacer adjacent motif (PAM) in red. Bold face print is used for the coding sequence of DYRK1B.

Vector construction: FUW-tetON-GFP-2A-hDYRK1B and mRFX7

The generation of lentiviral expression vectors with tetON-inducible expression of GFP-2A-hDYRK1B was carried out by modifying the FUW-tetON-hMYC plasmid (gift from Rudolf Jaenisch, Addgene plasmid # 20723). The cDNA encoding hMYC was exchanged for the GFP-2A-hDYRK1B or GFP-2A-mRFX7 cassette (Fig. S4) using NEBuilder HiFi DNA assembly. Murine cDNA clones for wild type RFX7 (pEF-DEST51-mRfx7) and the ΔNLS mutant were kindly provided by Greta Guarda (Castro et al. 2018).

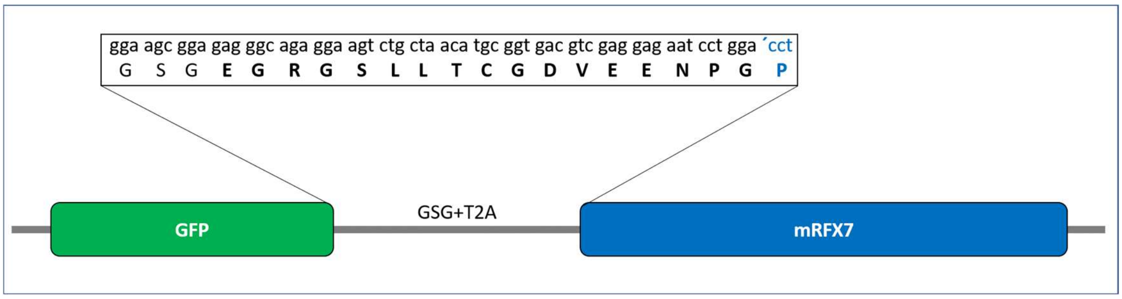

Fig. S5: Expression cassette used for inducible co-overexpression of GFP and mRFX7 The viral 2A sequence (shown in bold face print) induces ribosomal skipping during translation, resulting in stoichiometric co-expression of the proteins. The apostrophe marks the site of the skipped peptide bond.

Vector construction: pcDNA5/FRT/TO Flag-mRFX7-HiBiT

The murine RFX7 cDNAs (wild type and ΔNLS) were subcloned from pEF-Dest51-mRFX7-V5 using NEBuilder HiFi DNA assembly. The NLS-mutated version carries 5 point mutations that disrupt the bipartite nuclear localization motif characterized by Castro et al (Fig. S5).

```
mRFX7_ΔNLS   653   CTKSPAAALSATLQESQVPPVAAPIVEQLSAV  684
                   |||||   |||||||||||||  |||||||||
mRFX7_WT     653   CTKSPRKRLSATLQESQVPPVKKPIVEQLSAV  684
                   |||||||||| |||| ||||||||||||||| 
hRFX7        652   CTKSPRKRLSSTLQETQVPPVKKPIVEQLSAA  683
```

Fig. S5: Mutation of the bipartite nuclear localization sequence in RFX7-ΔNLS. The sequence is well conserved in mouse and human RFX7.

Vector construction: lentiCRISPR v2 RFX7

A suitable gRNA for CRISPR/Cas9-mediated knockout of RFX7 (gRNA CTGGATTCGGAATACCCTAG, PAM sequence AGG, targeting the DNA binding domain of human RFX7) was identified using the Broad Institute sgRNA design tool CRISPick (Doench et al. 2016, Sanson et al. 2018). For integration of the RFX7 gRNA into the lentiCRISPR v2 vector (gift from Feng Zhang, Addgene plasmid #52961), a modified protocol from the Zhang laboratory was used (Shalem et al. 2014; Sanjana et al., 2014). Single-stranded oligonucleotides were phosphorylated, annealed and ligated into the *Bsm*BI-linearized vector to produce lentiCRISPR v2-RFX7. The lentiCRISPR v2 NT control plasmid with a non-targeting (NT) control sequence (TTCCGGGCTAACAAGTCCT, Wesely et al. 2017) was kindly provided by Athanassios Fragoulis (Institute of Anatomy and Cell Biology, RWTH Aachen University, Aachen, Germany).

Lentivirus production and transduction

Lentiviral particles were produced using 7 × 10^6 HEK293T cells seeded in collagen-coated 25 cm^2 cell culture dishes. Co-transfection of lentiviral expression plasmids, namely FUW-tetON, FUW-M2rtTA or lentiCRISP v2 together with packaging plasmids (pMD2.G, psPAX2) in 1:1:1 plasmid molar ratio was carried out using JetPEI. Media was changed 6 h post-transfection, and culture supernatants were collected after 48 h and centrifuged at 4 000 g for 10 minutes at 4°C. The supernatants were sterile-filtered using a 0.45 µm filter and viral particles were collected by centrifugation (2 h, 26 000 rpm, 4°C). Pelleted viral particles were air-dried, resuspended in 50 µl ice-cold PBS, and aliquoted in 5 µl portions for storage at –80°C. Transduction in A549 cells was performed using polybrene (5 µg/ml).

RNA-seq

RNA concentration and integrity was verified on a TapeStation (Agilent) and QFX fluorometer (Denovix). Libraries for RNA-Seq were prepared using the QuantSeq FWD V2 kit (Lexogen) according to the manufacturer's protocol. Sequencing was performed on an Illumina NovaSeq6000 (1×118 cycles), generating approximately 8-10 million raw reads per sample. Raw data were demultiplexed and FASTQ files were generated using bcl_convert. Data were aligned to the GRCh38p14 genome and counted with STAR Aligner (Dobin et al. 2013) and further analyzed and visualized with BioJupies using default parameters (Torre et al. 2018). For differential expression (DE) analysis at least three independent biological replicates (n=3) of each treatment were used. To calculate DE, we compared the treatments against each other. BioJupies uses *limma* for differential gene expression analysis (Ritchie et al. 2015). In brief, raw read counts are converted to CPM and normalized by the voom function. A gene-wise linear model was used to assess DE. DE is estimated by an empirical Bayes framework, including a robust shrinkage, before utilizing t-statistics for DE assessment.

Proteomics

<u>Sample preparation for LC-MS/MS analysis</u>

A549 tetON cells were harvested 24 h after treatment by adding 1 ml of Accutase, pelleted by centrifugation (4 °C, 150 g, 10 min), and subsequently frozen at −80 °C. Pelleted cells were lysed with guanidine hydrochloride (GuHCl) lysis buffer containing 6 M GuHCl (Merck), 5 mM Bond-Breaker TCEP solution (Thermo), 10 mM 2-chloroacetamide (Merck), and 100 mM Tris-HCl buffer pH 8.5. Samples were incubated at 90 °C and 800 rpm in a thermo shaker for 10 minutes. DNA was sheared by incubation with Benzonase (15 U/sample, Merck, #E1014-25KU) for 30 minutes at 37 °C, and the samples were centrifuged at 15,000 g for 10 minutes. Supernatants were transferred to fresh tubes, and protein concentrations were determined with the Pierce 660 nm protein assay (Thermo). Samples were diluted to < 1 M GuHCl with 25 mM Tris-HCl, and trypsin / Lys-C (Promega) was added in a 1:100 enzyme-to-protein ratio and digested at 37 °C overnight. The next day, samples were acidified with formic acid (final concentration of 1 %) and desalted using C18 cartridges (Waters). In brief, the C18 sorbent was activated with 1 ml each of 100 % isopropanol and washed twice with buffer A (0.1 % formic acid in LC-MS-grade water). Solvents were pushed through the cartridges by applying positive pressure. Samples were loaded completely onto the cartridges and washed twice with buffer A. For peptide elution, buffer B (80% acetonitrile, 0.1% formic acid in LC-MS-grade water) was

added to the cartridges and the flow-through was collected in clean reaction tubes. Samples were dried completely by vacuum centrifugation, redissolved in buffer R (2% acetonitrile, 0.1% formic acid in LC-MS-grade water), and peptide concentrations were determined. For each LC-MS/MS measurement, 200 ng of peptides were injected into the LC-MS system.

Liquid chromatography-mass spectrometry:

A liquid chromatography-tandem mass spectrometry system consisting of a Vanquish Neo UHPCL operated in trap mode, and an Orbitrap Exploris 480 MS (both Thermo Fisher Scientific) was used for proteomics analysis. Peptides were separated with a 90-minute chromatographic gradient consisting of a binary buffer system with buffer A and buffer B. An IonOpticks Aurora Ultimate analytical column was used for peptide separation. Starting at 5% buffer B, the amount of B was linearly increased to 25 % over 71 minutes, followed by a linear increase to 55% over 9 minutes. Lastly, the analytical column was washed at 95% B for 10 minutes.

Eluting peptides were online injected into the mass spectrometer by electrospray ionisation using a nanoFlex ion source operated at 2.5 kV. The MS was operated in data-independent acquisition (DIA) mode with staggered DIA windows. In brief, full spectra were acquired at a resolution of 120,000, an injection time of 55 ms and an AGC target of 300%. Ions were collected within a range of 380 – 1,020 m/z. For MS/MS fragment spectra, ions were isolated from 24 equal-sized windows of 24 m/z within a range of 400 – 1,000 m/z. The acquisition was performed at a resolution of 15,000, an injection time of 22 ms and an AGC target of 1,000%. Fragmentation was achieved by higher-energy collisional dissociation (HCD) at 31% relative collision energy. For every second cycle, the isolation windows for MS/MS acquisition were shifted by 12 m/z.

Data processing and statistical analysis

MS raw data processing was performed in Spectronaut (version 19.1.240724.62635) using the implemented directDIA search strategy. For spectral library generation, the SWISS-PROT *Homo sapiens* database was used (downloaded 2024-08-01). The default settings for peptide identification and quantification were used. Protein group intensities were LFQ (label-free quantification) normalised with directLFQ (Ammar et al. 2023).

The statistical analysis was performed with Perseus (v. 1.6.15, Tyanova et al. 2016). Protein LFQ intensities were log2 transformed and filtered for data completeness in at least one condition. Missing protein intensities were replaced sample-wise by random selection of values from 1.8 standard deviations downshifted, 0.3 standard deviations broad normal distribution. Identification of significantly enriched proteins was performed by Welch's t-testing (S0 = 0.1, permutation-based FDR correction = 0.05, with 250 randomisations). 1D annotation enrichment tests (Benjamini-Hochberg FDR = 0.02, Cox and Mann 2012) were performed for identification of enriched RFX7 target gene sets (selection based on Coronel et al. 2021; Schwab et al. 2023). Statistical differences from multiple comparisons in Figure 6E were identified, as previously described in the statistical methods section, using a generalized mixed model (PROC GLIMMIX, SAS 9.4, SAS Institute Inc., Cary, NC, USA) based on $\log_2$-transformed LFQ intensities. Statistical significance for Figure 6B was determined using a Wilcoxon matched-pairs signed rank test (GraphPad Prism 9; GraphPad Software, San Diego, California, USA).

Interactomics

Sample preparation for LC-MS/MS analysis

Flag-RFX7 was immunoprecipitated from transiently transfected HEK293tsa cells as described in the method section of the main paper. A total of 50 µl Urea lysis buffer (8 M urea, 10 mM Bond-Breaker TCEP solution, 30 mM CAA in 50 mM Tris-HCl buffer pH 8.5) was added to the pelleted anti-FLAG M2 affinity beads (Sigma-Aldrich) and incubated at room temperature in a thermo shaker at 800 for 30 min. Following, samples were equally split, diluted with 50 mM Tris-HCl to a concentration of <2 M Urea, and digested either with trypsin/Lys-C or chymotrypsin (both 500 ng/sample, Promega) at room temperature and 800 g in a thermo shaker overnight. The next day, samples were acidified with formic acid (final concentration 0.5 %) and desalted using a modified C18 StageTip protocol. In-house-made polystyrene-divinylbenzene reversed phase sulfonate (SDB-RPS, Affinisep) double-layer StageTips were activated with 100 µl methanol and washed twice with 100 µl buffer B. The tips were equilibrated with 100 µl buffer A, and samples were loaded onto the StageTips. Bound peptides were washed once with 200 µl buffer A and twice with 200 µl buffer B. After each step, samples were centrifuged at 500 g for 1 min or until all liquid passed through the SDB-RPS material. Peptides were eluted with 60 µl buffer X (80% ACN, 5% ammonia in LC-MS-grade water) and vacuum-dried in a vacuum centrifuge. Peptides were resuspended in 10 µl buffer R, peptide concentrations were determined, and 200 ng was injected into the LC-MS systems.

Liquid chromatography-mass spectrometry:

The same LC-MS system was used, as described earlier, but operated in data-dependent acquisition (DDA) mode. A 60-minute chromatographic gradient was used where buffer B was linearly increased from 2 – 34% over 41 minutes, followed by an increase to 50% over 6 minutes. For the remaining 13 minutes, the analytical column was washed at 95% buffer B. Full MS were acquired at a resolution of 60,000, an injection time of 25 ms and an AGC target of 300%. Per cycle, 20 potential precursor ions were selected for fragmentation from a range of 350 – 1,750 m/z with an isolation width of 1.4 m/z. Fragment spectra were acquired at a resolution of 15,000, an injection time of 22 ms and an AGC target of 100%. Peptides were fragmented by HCD operated at 27%.

Data processing and statistical analysis

Raw data was processed in MaxQuant using the implemented Andromeda search engine (version 2.4.3.0; Cox et al. 2008, Cox et al. 2011). Default settings were applied, except trypsin or chymotrypsin were selected as digestion enzymes. The SWISS-PROT *Homo sapiens* database (downloaded 2023-02-15) was used, complemented with the *Mus musculus* RFX7 sequence (F8VPJ6), replacing the human RFX7 version.

The statistical analysis was performed with Perseus as described earlier. Identification of significantly enriched proteins was performed by Welch's t-testing (S0 = 0.1, permutation-based FDR correction = 0.05, with 250 randomisations).

Modeling of binary protein-protein interactions

Binary protein-protein interactions of RFX7 were modeled using AlphaFold Multimer. The full amino acid sequences of the proteins of interest were retrieved from UniProt (UniProt, 2024): RFX7: Q2KHR2; ANKRA2: Q9H9E1; RFXAP: O00287, RFXANK: O14593 and DYRK1A: Q13627; DYRK1B: Q9Y463; DCAF7: P61962; HSPA8: P11142; HSPA9: P38646; LETM1: O95202 and NDUFA5: Q16718. The ab initio prediction of the structure and interactions of all protein pairs of interest was performed using AlphaFold Multimer (Jumper et al., 2021) in a high-throughput, batch-based pipeline, which was developed using the ColabFold notebook v1.5.5 (Mirdita et al., 2022). The modelling was performed using MMseq2 for multiple sequence alignment without homology templates. The prediction models were ranked according to the ipTM (Interface Predicted Template Modelling) score. A custom code was used to extract CA atom coordinates from chains A and B in order to identify pairs of residues within 10 Å of each other, and to calculate the average interface PAE (predicted alignment error) for each model. These pairs were then clustered into putative interfaces based on spatial continuity. For each identified interface, the average interface PAE (iPAE) of all residues involved was calculated. An overall interaction confidence score was calculated by using the weighted average of the normalised iPAE score and the ipTM scores, with a weighting of 50:50. The iPAE was normalised by assigning a value of 1 to scores ≤5, a value of 0 to scores >15, and linearly scaling scores for values between 5 and 15.

References :

Ammar C, Schessner JP, Willems S, Michaelis AC, Mann M. Accurate Label-Free Quantification by directLFQ to Compare Unlimited Numbers of Proteomes. Mol Cell Proteomics. 2023 22(7):100581. doi: 10.1016/j.mcpro.2023.100581.

Bartha Á, Győrffy B. TNMplot.com: A Web Tool for the Comparison of Gene Expression in Normal, Tumor and Metastatic Tissues. Int J Mol Sci. 2021 22(5):2622. doi: 10.3390/ijms22052622.

Becker W, Weber Y, Wetzel K, Eirmbter K, Tejedor FJ, Joost HG. Sequence characteristics, subcellular localization, and substrate specificity of DYRK-related kinases, a novel family of dual specificity protein kinases. J Biol Chem. 1998 273(40):25893-902. doi:10.1074/jbc.273.40.25893.

Castro W, Chelbi ST, Niogret C, Ramon-Barros C, Welten SPM, Osterheld K, Wang H, Rota G, Morgado L, Vivier E, Raeber ME, Boyman O, Delorenzi M, Barras D, Ho PC, Oxenius A, Guarda G. The transcription factor Rfx7 limits metabolism of NK cells and promotes their maintenance and immunity. Nat Immunol. 2018 19(8):809-820. doi: 10.1038/s41590-018-0144-9.

Coronel L, Riege K, Schwab K, Förste S, Häckes D, Semerau L, Bernhart SH, Siebert R, Hoffmann S, Fischer M. Transcription factor RFX7 governs a tumor suppressor network in response to p53 and stress. Nucleic Acids Res. 2021 49(13):7437-7456. doi: 10.1093/nar/gkab575.

Cox J, Mann M. 1D and 2D annotation enrichment: a statistical method integrating quantitative proteomics with complementary high-throughput data. BMC Bioinformatics. 2012;13 Suppl 16:S12. doi: 10.1186/1471-2105-13-S16-S12.

Cox J, Mann M. MaxQuant enables high peptide identification rates, individualized p.p.b.-range mass accuracies and proteome-wide protein quantification. Nat Biotechnol. 2008 26(12):1367-72. doi: 10.1038/nbt.1511.

Cox J, Neuhauser N, Michalski A, Scheltema RA, Olsen JV, Mann M. Andromeda: a peptide search engine integrated into the MaxQuant environment. J Proteome Res. 2011 10(4):1794-805. doi: 10.1021/pr101065j.

Dobin A, Davis CA, Schlesinger F, Drenkow J, Zaleski C, Jha S, Batut P, Chaisson M, Gingeras TR. STAR: ultrafast universal RNA-seq aligner. Bioinformatics. 2013 29(1):15-21. doi: 10.1093/bioinformatics/bts635.

Doench, J. G., Fusi, N., Sullender, M., Hegde, M., Vaimberg, E. W., Donovan, K. F., et al. Optimized sgRNA design to maximize activity and minimize off-target effects of CRISPR-Cas9. Nature biotechnology 2016 34(2):184-191. doi:10.1038/nbt.3437

Jumper J, Evans R, Pritzel A, Green T, Figurnov M, Ronneberger O, Tunyasuvunakool K, et al. Highly accurate protein structure prediction with AlphaFold. Nature. 2021 596(7873):583-589. doi: 10.1038/s41586-021-03819-2.

Leder S, Weber Y, Altafaj X, Estivill X, Joost HG, Becker W. Cloning and characterization of DYRK1B, a novel member of the DYRK family of protein kinases. Biochem Biophys Res Commun. 1999 254(2):474-9. doi: 10.1006/bbrc.1998.9967.

Leder S, Czajkowska H, Maenz B, De Graaf K, Barthel A, Joost HG, Becker W. Alternative splicing variants of dual specificity tyrosine phosphorylated and regulated kinase 1B exhibit distinct patterns of expression and functional properties. Biochem J. 2003 372:881-8. doi: 10.1042/BJ20030182.

Lin DW, Chung BP, Huang JW, Wang X, Huang L, Kaiser P. Microhomology based CRISPR tagging tools for protein tracking, purification, and depletion. J Biol Chem. 2019 294:10877-10885

Menyhart O, Kothalawala WJ, Győrffy B. A gene set enrichment analysis for cancer hallmarks. J Pharm Anal. 2025 15(5):101065. doi: 10.1016/j.jpha.2024.101065.

Mirdita M, Schütze K, Moriwaki Y, Heo L, Ovchinnikov S, Steinegger M. ColabFold: making protein folding accessible to all. Nat Methods. 2022 19:679-682. doi: 10.1038/s41592-022-01488-1.

Ritchie ME, Phipson B, Wu D, Hu Y, Law CW, Shi W, Smyth GK. limma powers differential expression analyses for RNA-sequencing and microarray studies. Nucleic Acids Res. 2015 43(7):e47. doi: 10.1093/nar/gkv007

Rüben K, Wurzlbauer A, Walte A, Sippl W, Bracher F, Becker W. Selectivity Profiling and Biological Activity of Novel β-Carbolines as Potent and Selective DYRK1 Kinase Inhibitors. PLoS One 2015 10:e0132453.

Sanjana NE, Shalem O, Zhang F. Improved vectors and genome-wide libraries for CRISPR screening. Nat Methods. 2014 11(8):783-784. doi: 10.1038/nmeth.3047.

Schwab K, Coronel L, Riege K, Sacramento EK, Rahnis N, Häckes D, Cirri E, Groth M, Hoffmann S, Fischer M. Multi-omics analysis identifies RFX7 targets involved in tumor suppression and neuronal processes. Cell Death Discov. 2023 9(1):80. doi: 10.1038/s41420-023-01378-1.

Sanson, K. R., Hanna, R. E., Hegde, M., Donovan, K. F., Strand, C., Sullender, M. E., et al. Optimized libraries for CRISPR-Cas9 genetic screens with multiple modalities. Nature Communications 2018 9(1):5416. doi:10.1038/s41467-018-07901-8

Sester S, Wilms G, Ahlburg J, Babendreyer A, Becker W. Elevated expression levels of the protein kinase DYRK1B induce mesenchymal features in A549 lung cancer cells. BMC Cancer. 2024 24(1):1341. doi: 10.1186/s12885-024-13057-0.

Shalem O, Sanjana NE, Hartenian E, Shi X, Scott DA, Mikkelson T, Heckl D, Ebert BL, Root DE, Doench JG, Zhang F. Genome-scale CRISPR-Cas9 knockout screening in human cells. Science. 2014 343:84-87. doi: 10.1126/science.1247005.

Subramanian A, Tamayo P, Mootha VK, Mukherjee S, Ebert BL, Gillette MA, Paulovich A, Pomeroy SL, Golub TR, Lander ES, Mesirov JP. Gene set enrichment analysis: a knowledge-based approach for interpreting genome-wide expression profiles. Proc Natl Acad Sci U S A. 2005 102(43):15545-50. doi: 10.1073/pnas.0506580102.

Torre D, Lachmann A, Ma'ayan A. BioJupies: Automated Generation of Interactive Notebooks for RNA-Seq Data Analysis in the Cloud. Cell Syst. 2018 7(5):556-561.e3. doi: 10.1016/j.cels.2018.10.007.

Tyanova S, Temu T, Sinitcyn P, Carlson A, Hein MY, Geiger T, Mann M, Cox J. The Perseus computational platform for comprehensive analysis of (prote)omics data. Nat Methods. 2016 13(9):731-40. doi: 10.1038/nmeth.3901.

Wesely J, Steiner M, Schnütgen F, Kaulich M, Rieger MA, Zörnig M. Delayed mesoderm and erythroid differentiation of murine embryonic stem cells in the absence of the transcriptional regulator FUBP1. Stem Cells Int. 2017 2017:5762301. doi: 10.1155/2017/5762301.

Wilms G, Schofield K, Maddern S, Foley C, Shaw Y, Smith B, Basantes LE, Schwandt K, Babendreyer A, Chavez T, McKee N, Gokhale V, Kallabis S, Meissner F, Rokey SN, Dunckley T, Montfort WR, Becker W, Hulme C. Discovery and Functional Characterization of a Potent, Selective, and Metabolically Stable PROTAC of the Protein Kinases DYRK1A and DYRK1B. J Med Chem. 2024 67(19):17259-17289. doi: 10.1021/acs.jmedchem.4c01130.
