# Supplementary material for: The protein kinase DYRK1B is a p53 target gene and functions as a negative feedback regulator of the transcription factor RFX7
Source: Cell Death Dis. 2026 Mar 26;17(1):386. doi: 10.1038/s41419-026-08660-x (PMC13066115; doi:10.1038/s41419-026-08660-x)
Supplement: Supplementary file 2 — Uncropped Western blots [file 41419_2026_8660_MOESM2_ESM.pdf]

Figure 1 A, B, Figure 2 A ;10%-gel

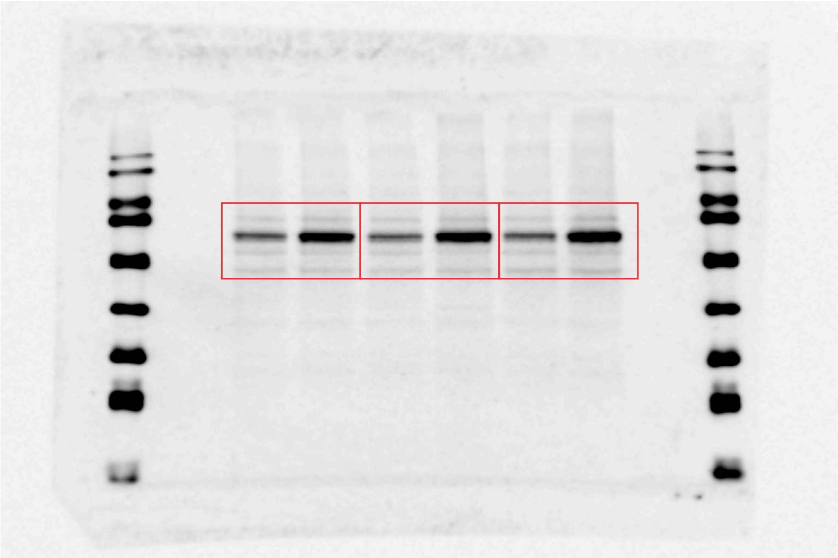

Anti-DYRK1B

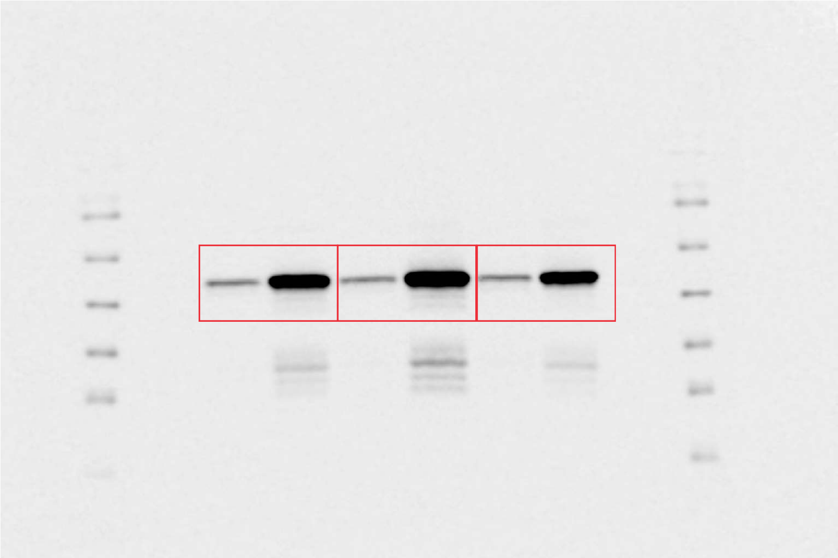

Anti-p53

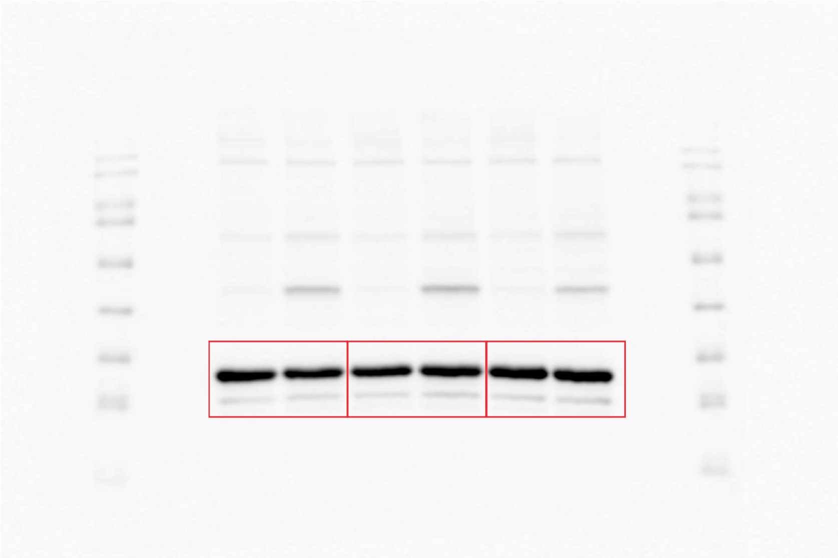

Anti-GAPDH

Figure 1 A, B, Figure 2 A ;16%-gel

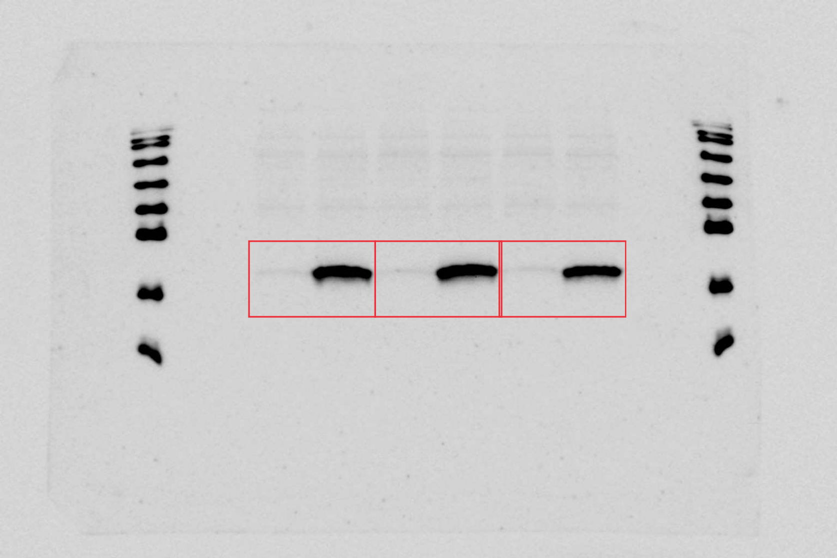

Anti-p21

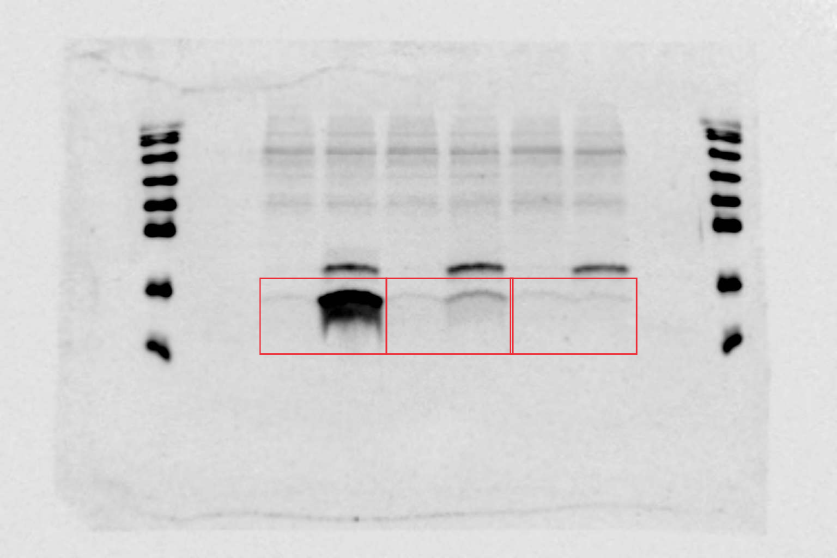

Anti-H2A.X-P(Ser139)

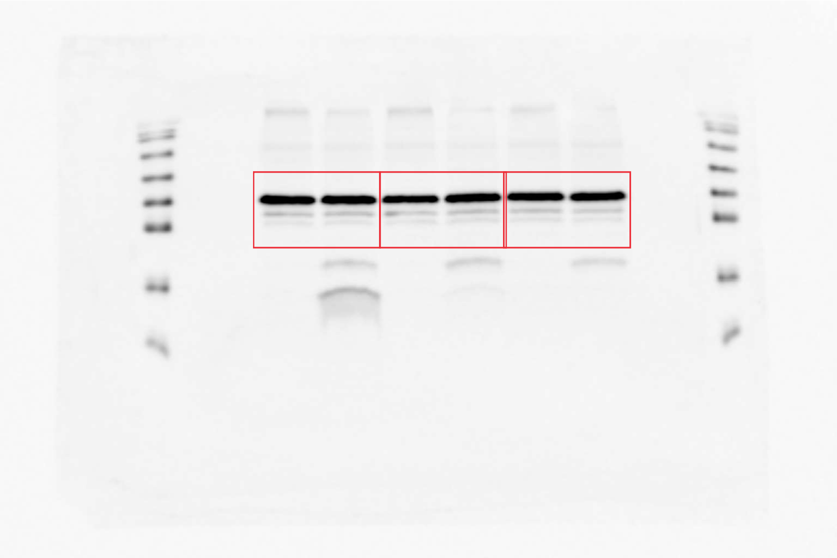

Anti-GAPDH

Figure 1 C, D, Figure 2 B ;10%-gel

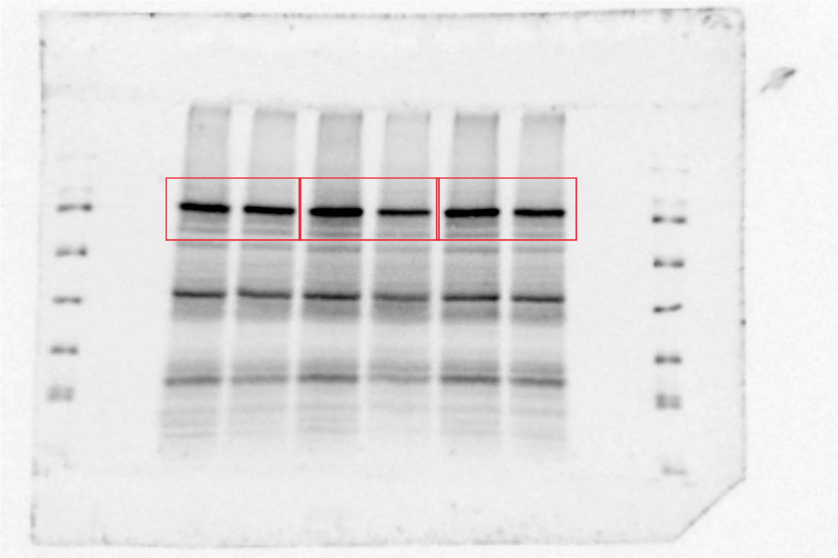

Anti-DYRK1A

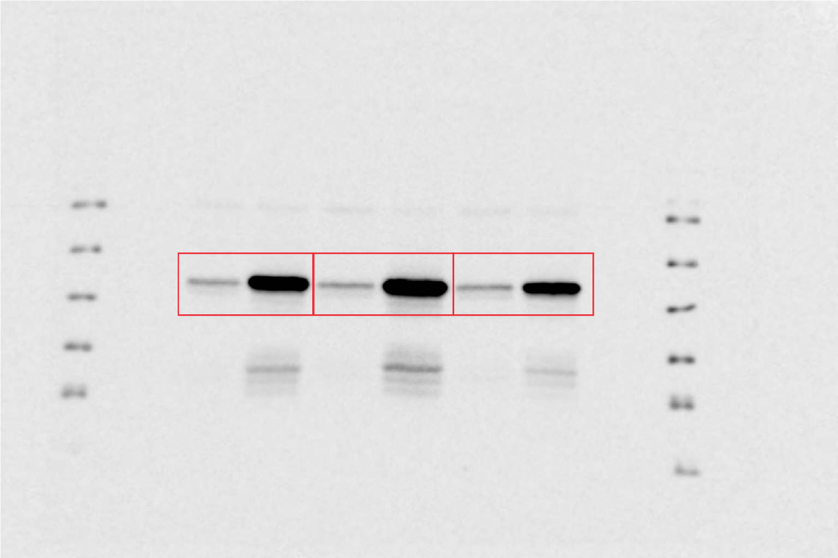

Anti-p53

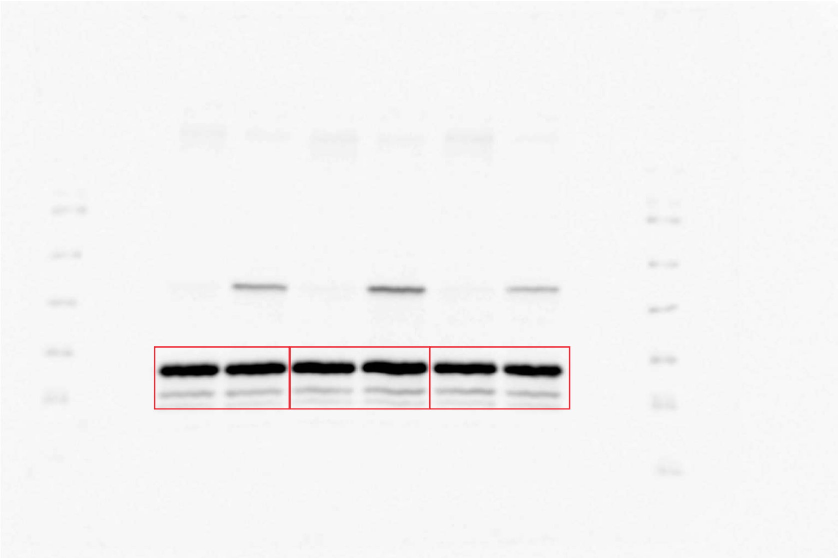

Anti-GAPDH

Figure 1 E ;10%-gel

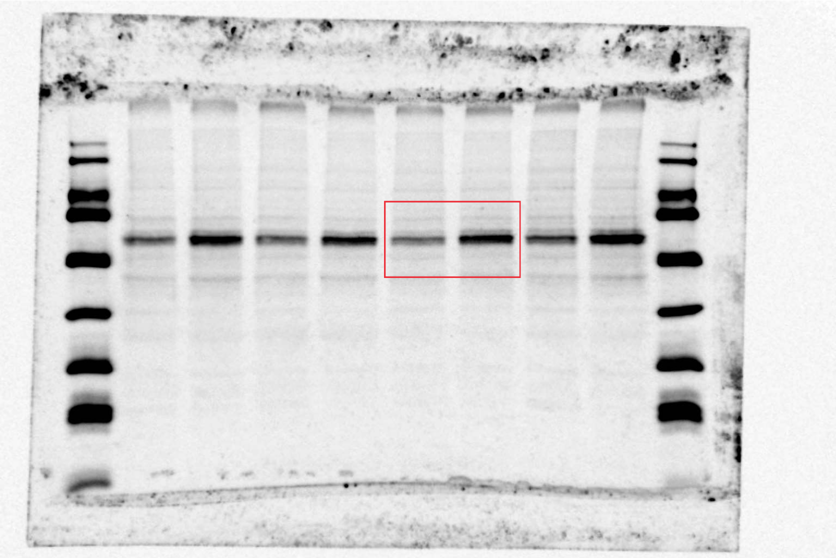

Anti-DYRK1B

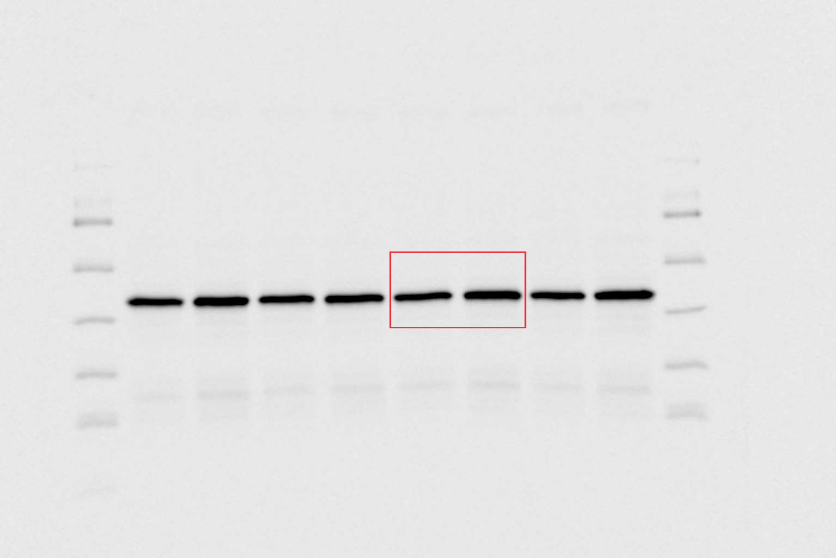

Anti-p53

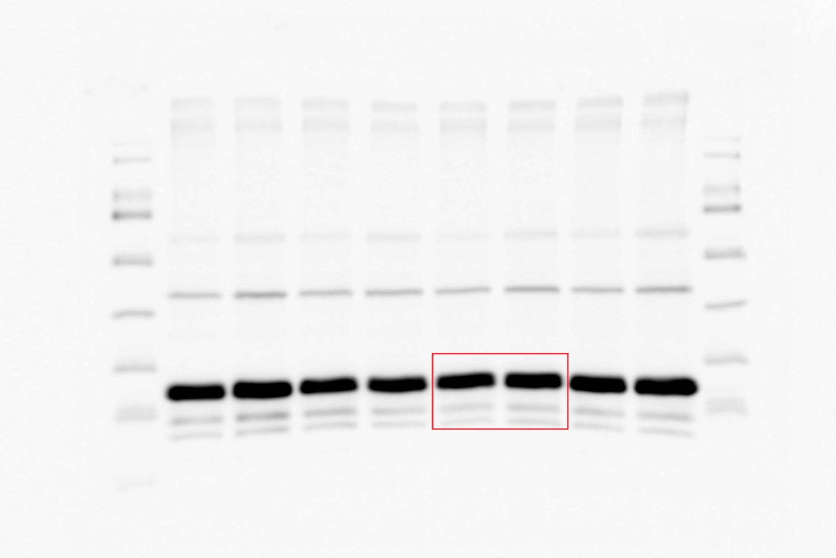

Anti-GAPDH

Figure 1 E ;16%-gel

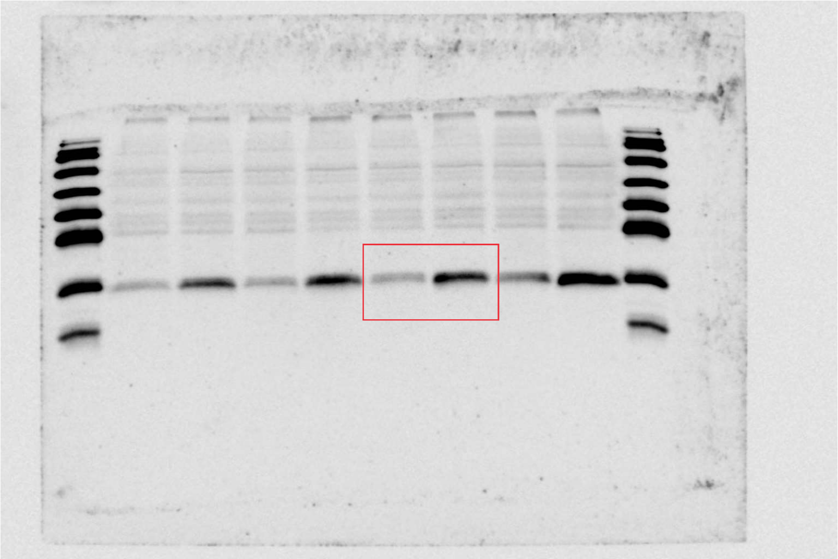

Anti-p21

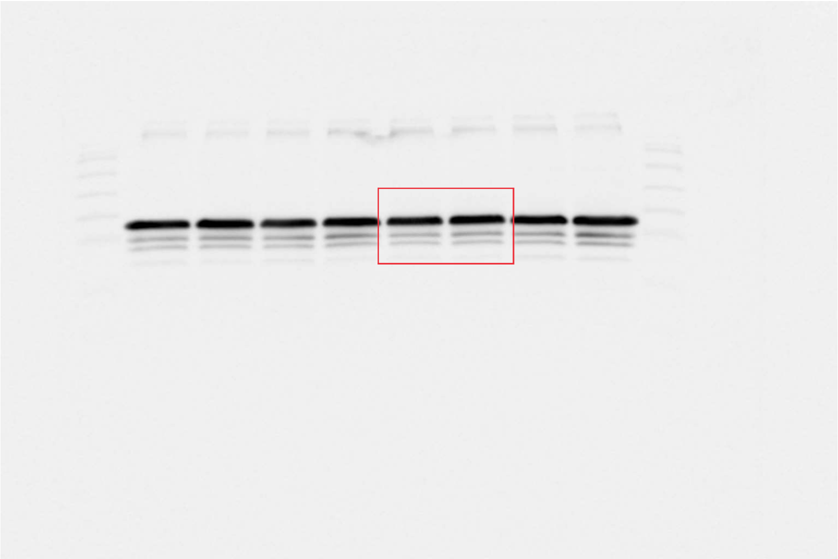

Anti-GAPDH

Figure 1 F ;10%-gel

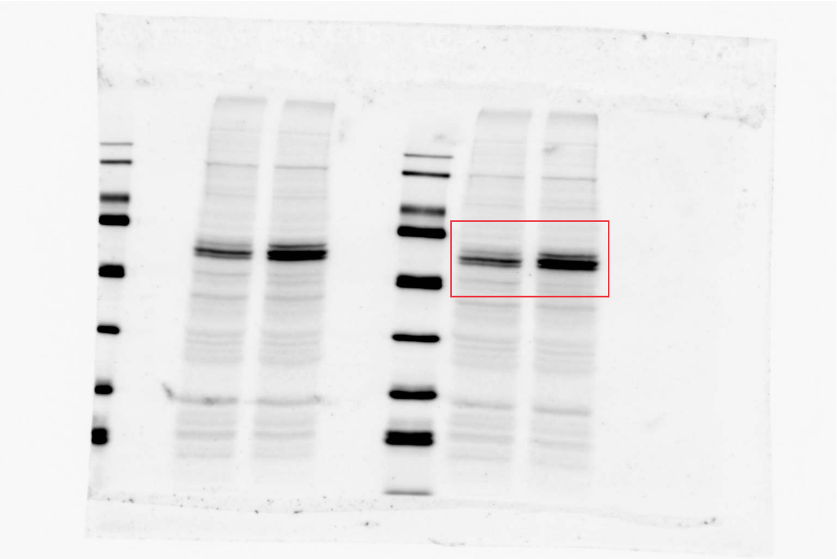

Anti-DYRK1B

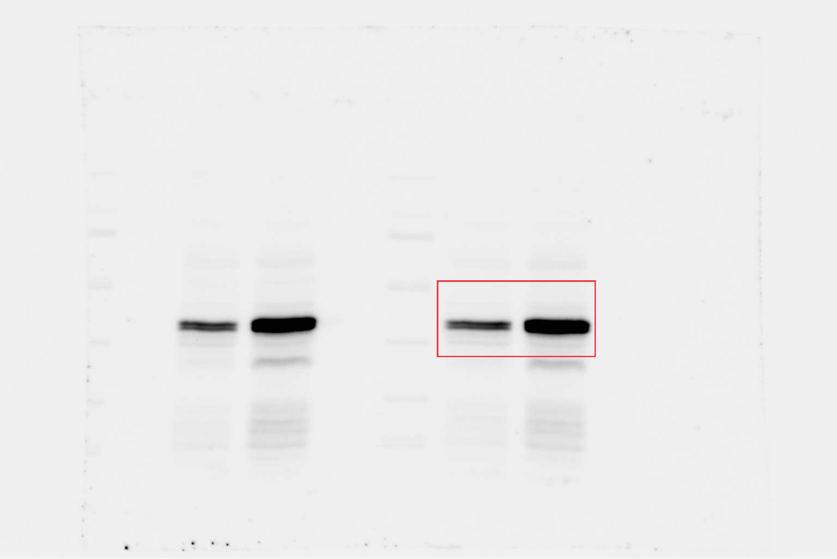

Anti-p53

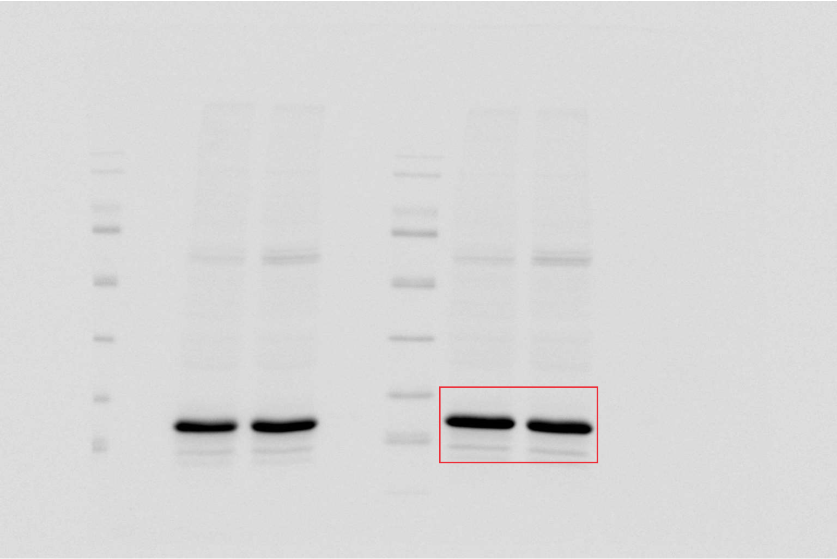

Anti-GAPDH

Figure 1 F ;16%-gel

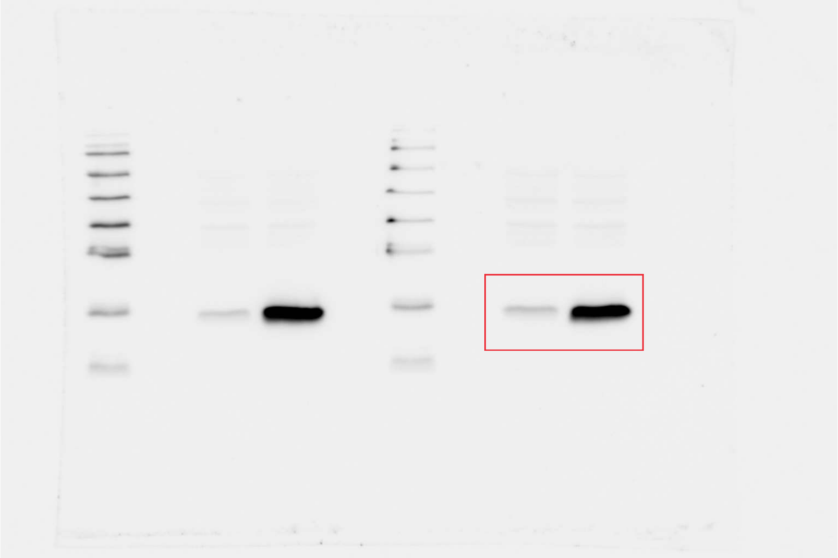

Anti-p21

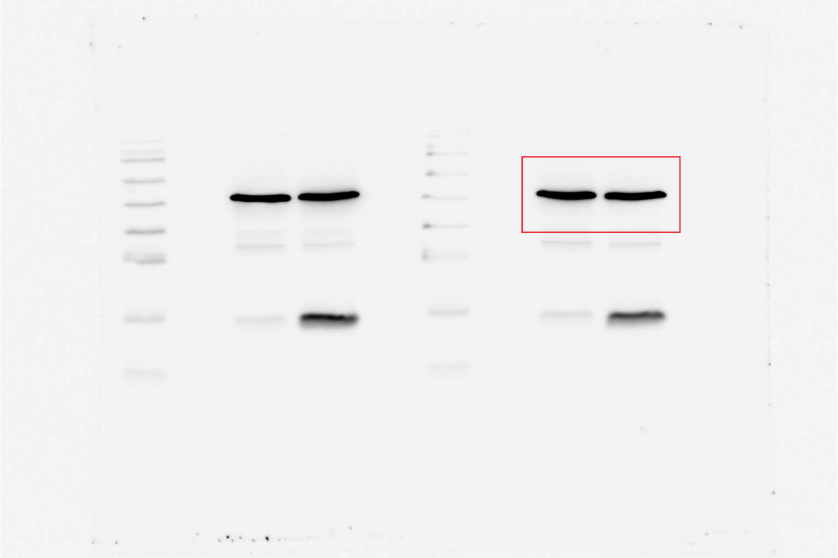

Anti-ß-Tubulin

Figure 1 H; 8%-gel (Input)

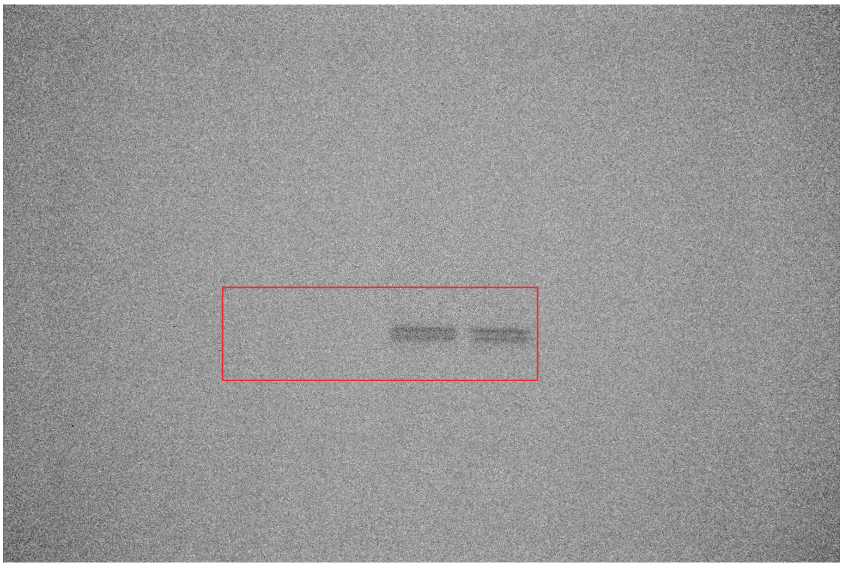

HiBiT-Blotting

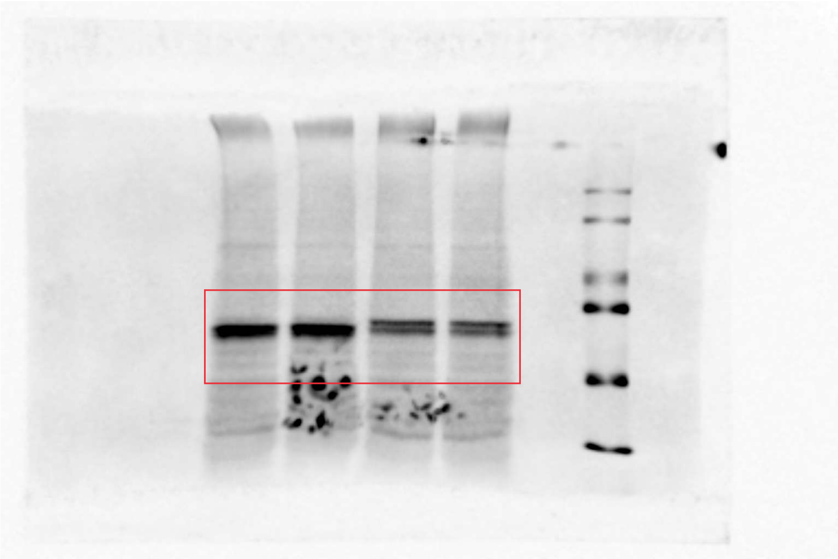

Anti-DYRK1B

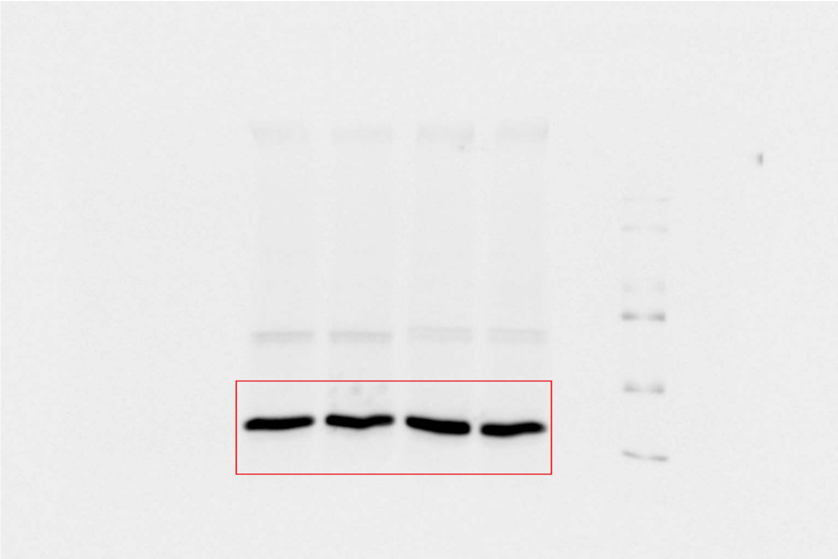

Anti-ß-Tubulin

Figure 1 H; 8%-gel (IP)

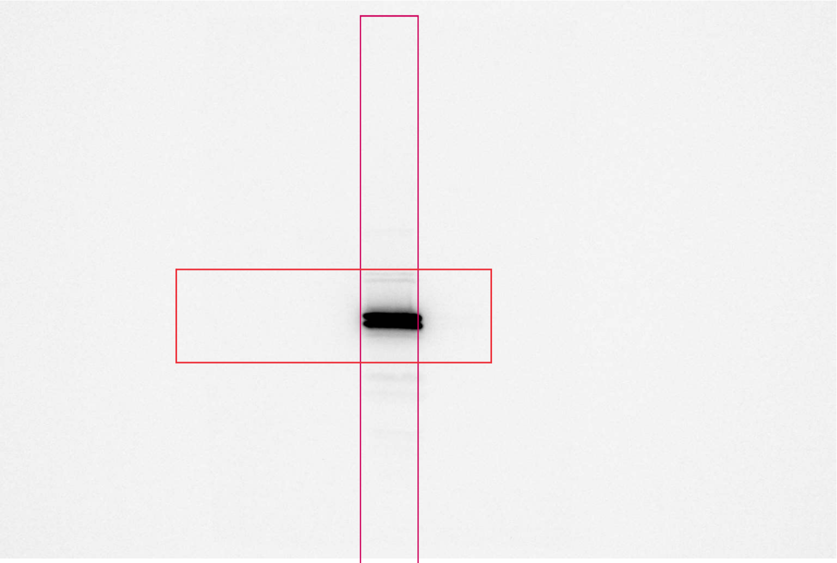

HiBiT-Blotting

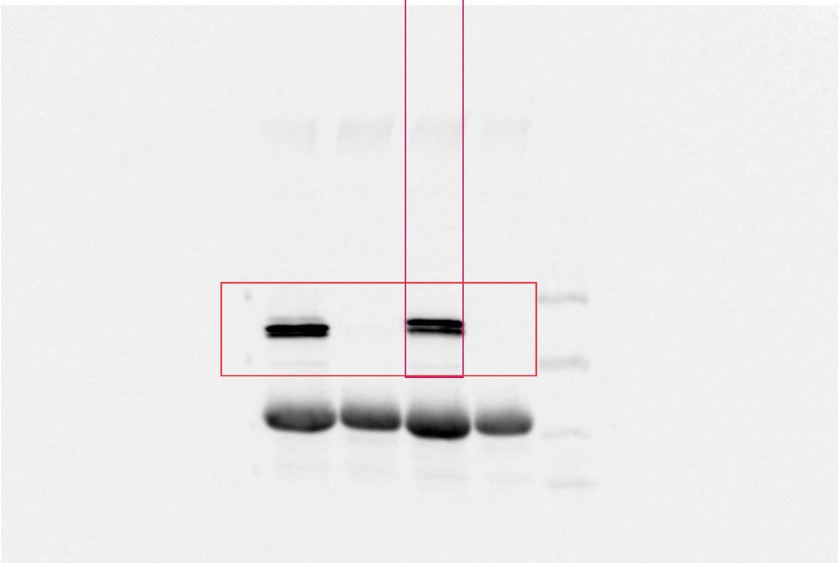

Anti-DYRK1B

Figure 2 E, F;10%-gel

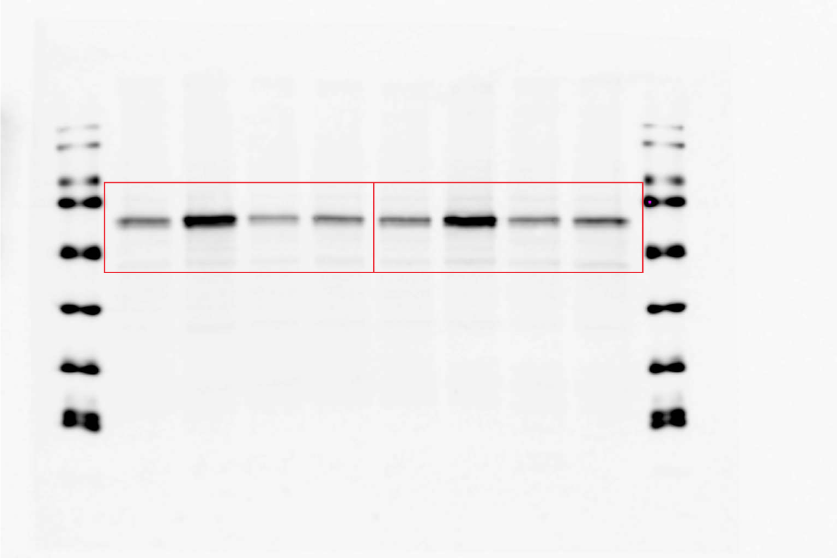

Anti-DYRK1B

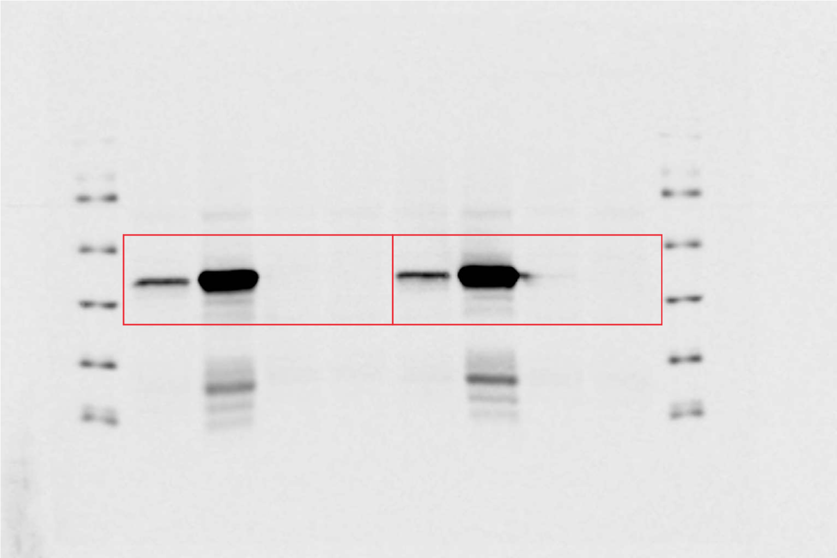

Anti-p53

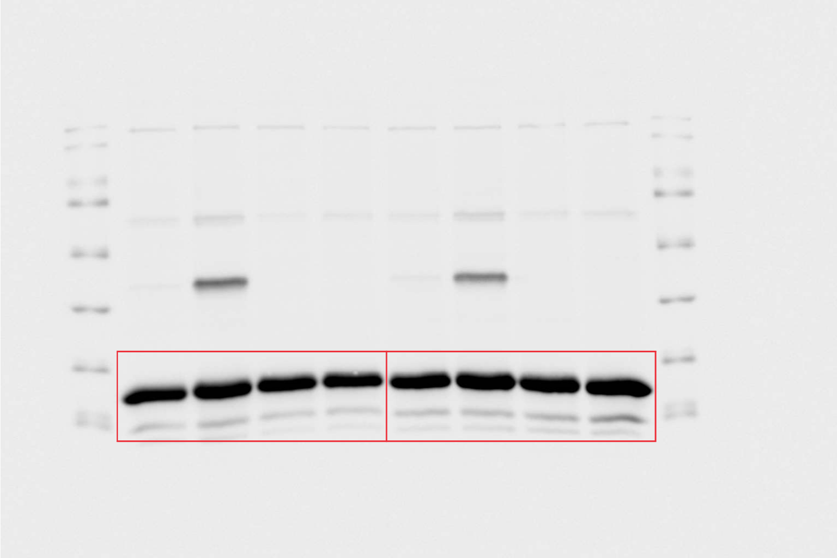

Anti-GAPDH

Figure 2 E, F;16%-gel

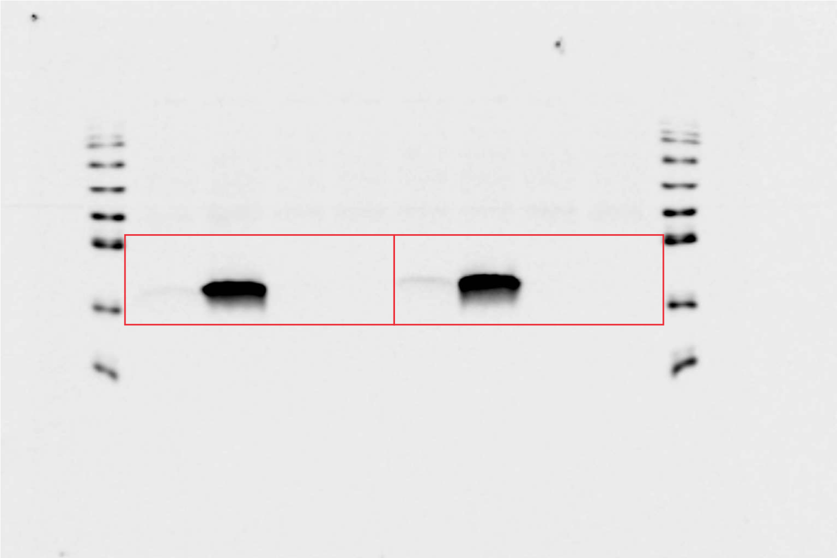

Anti-p21

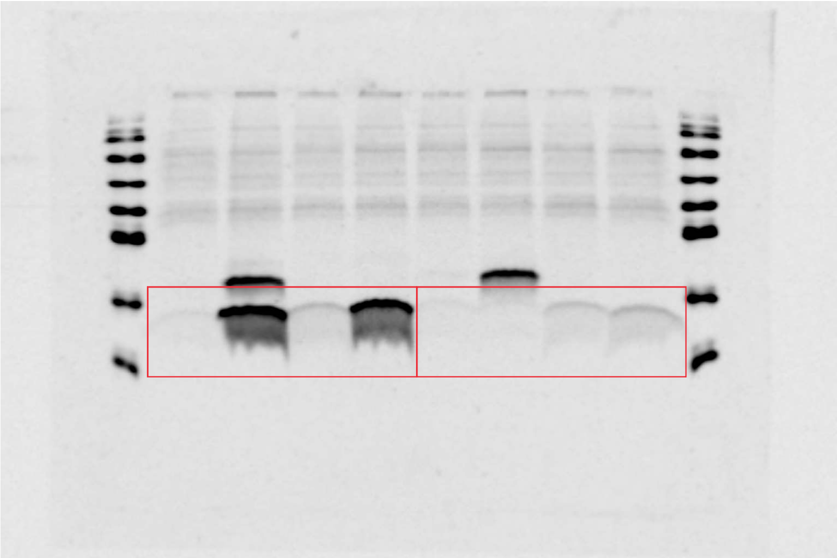

Anti-H2A.X-P(Ser139)

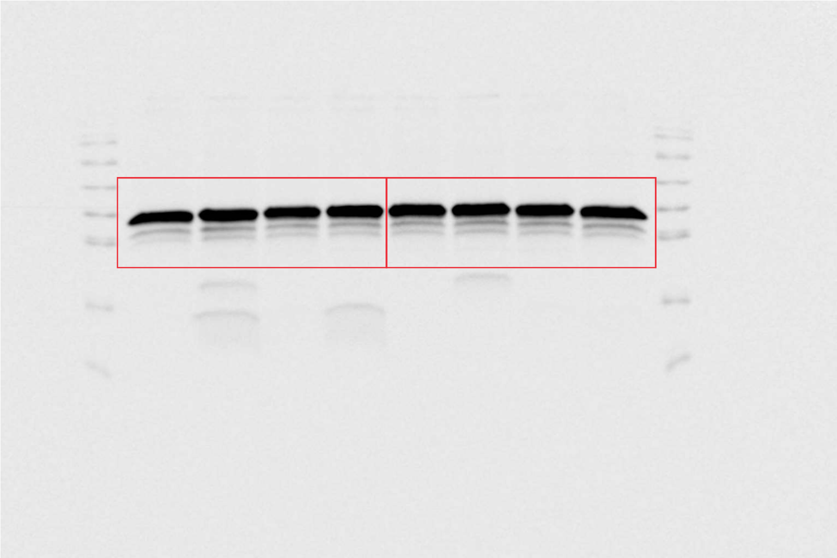

Anti-GAPDH

Figure 2 G;10%-gel

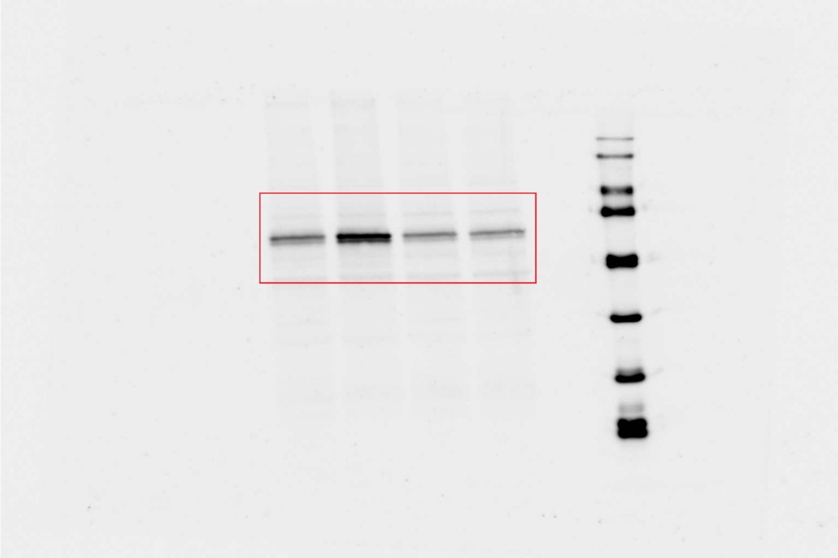

Anti-DYRK1B

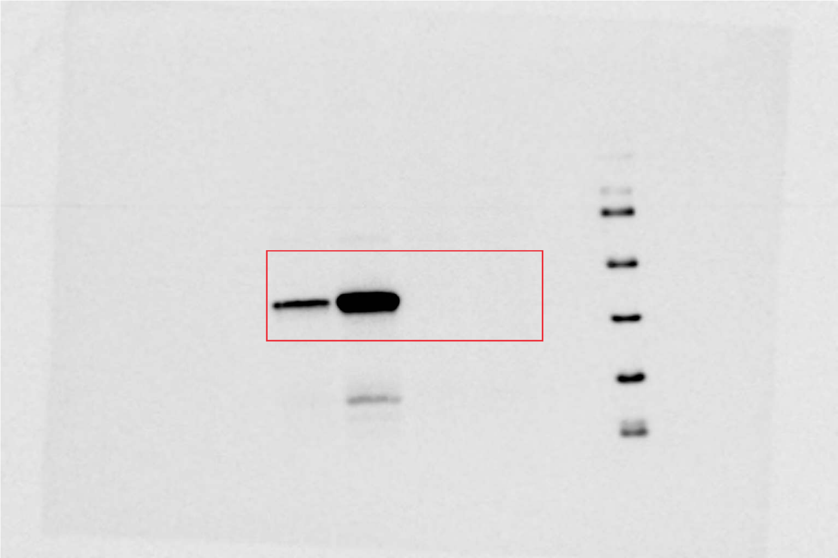

Anti-p53

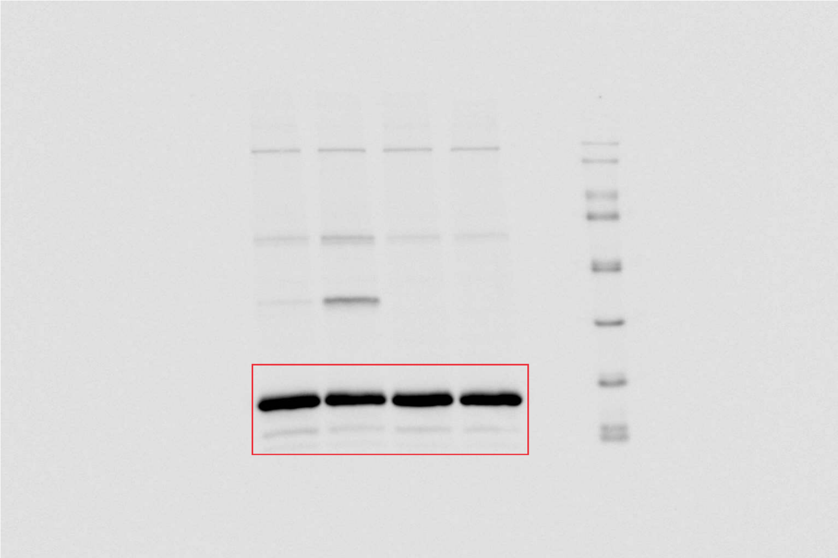

Anti-GAPDH

Figure 2 G;16%-gel

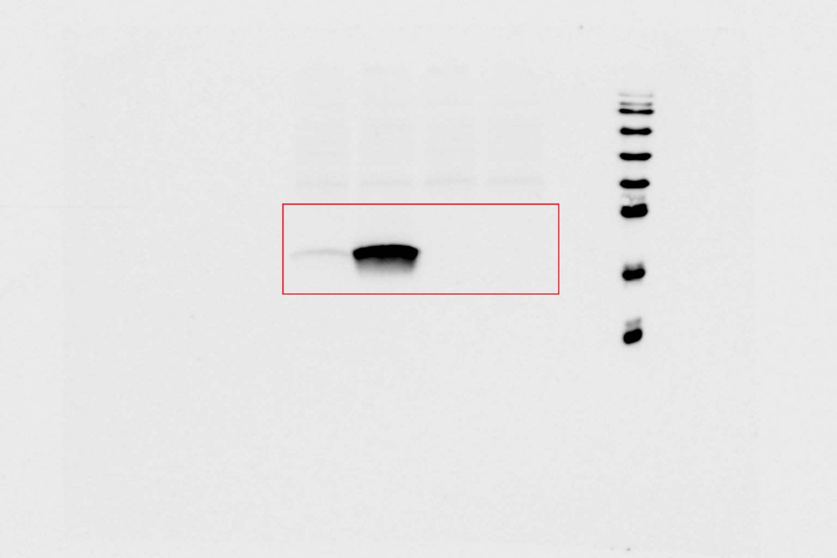

Anti-p21

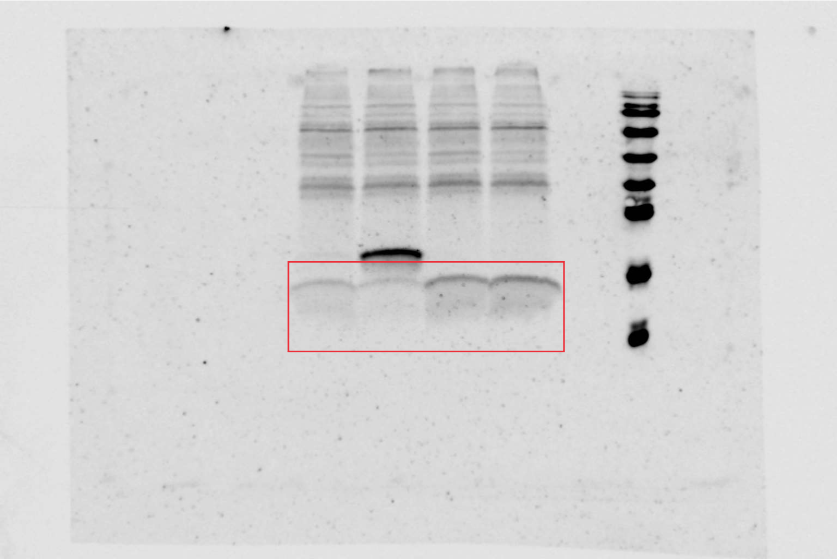

Anti-H2A.X-P(Ser139)

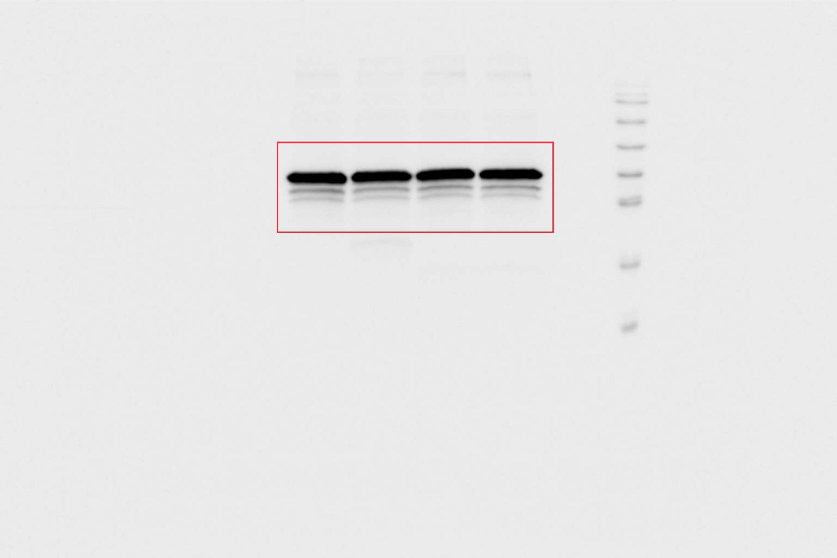

Anti-GAPDH

Figure 2 H; 10%-gel

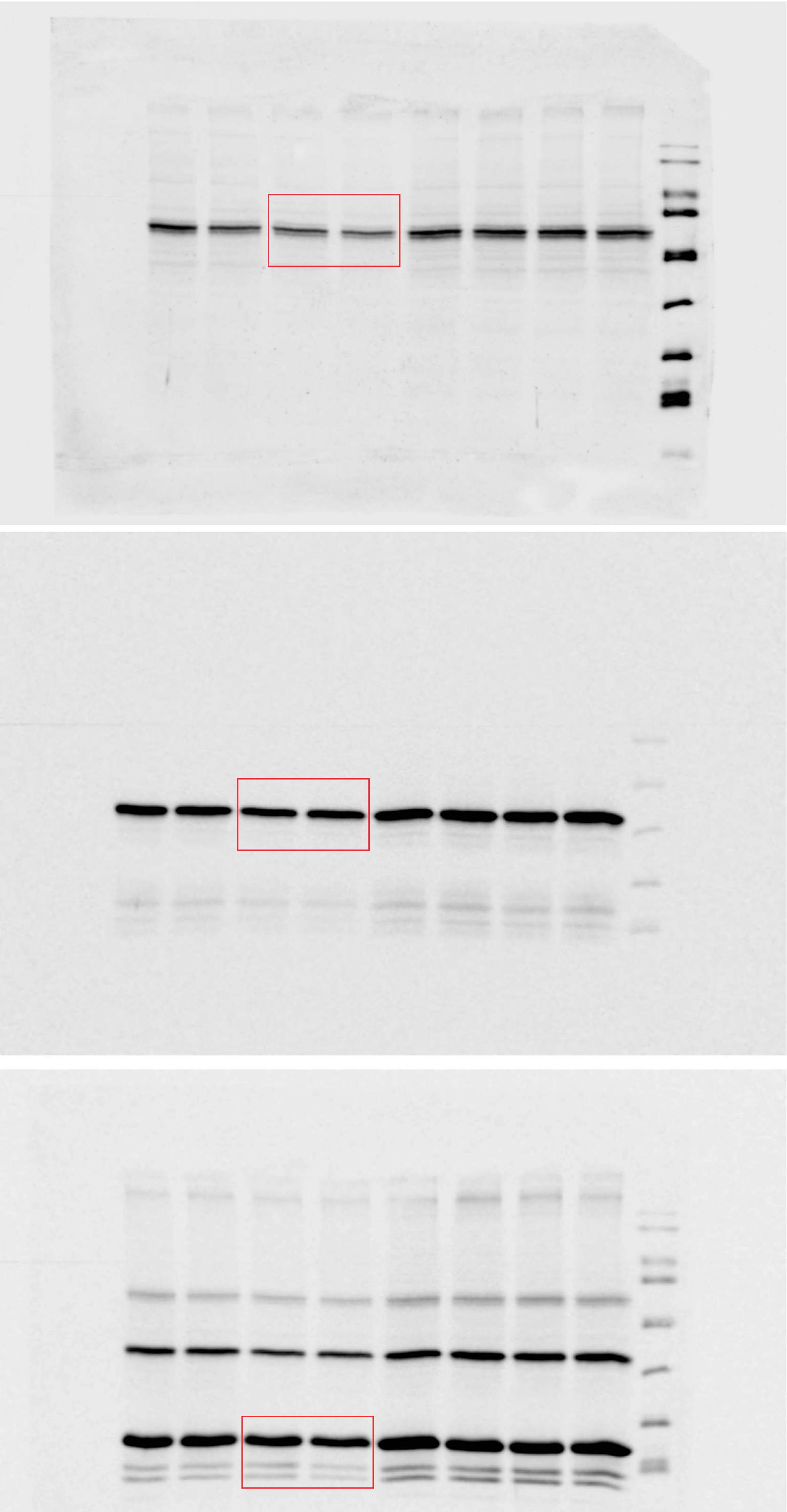

Anti-DYRK1B

Anti-p53

Anti-GAPDH

Figure 2 I; 10%-gel

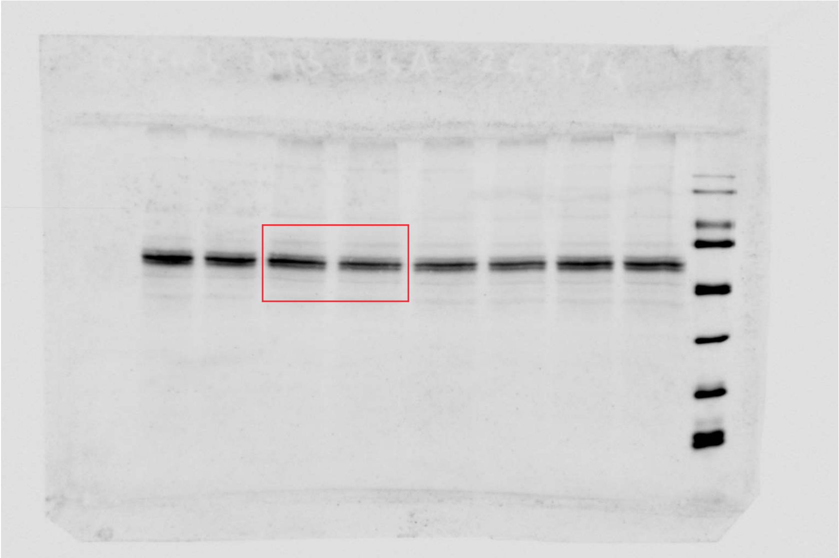

Anti-DYRK1B

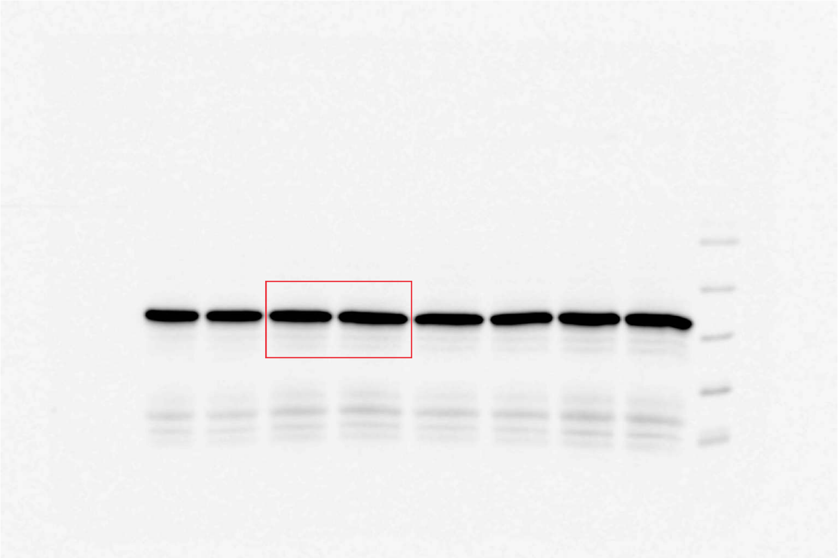

Anti-p53

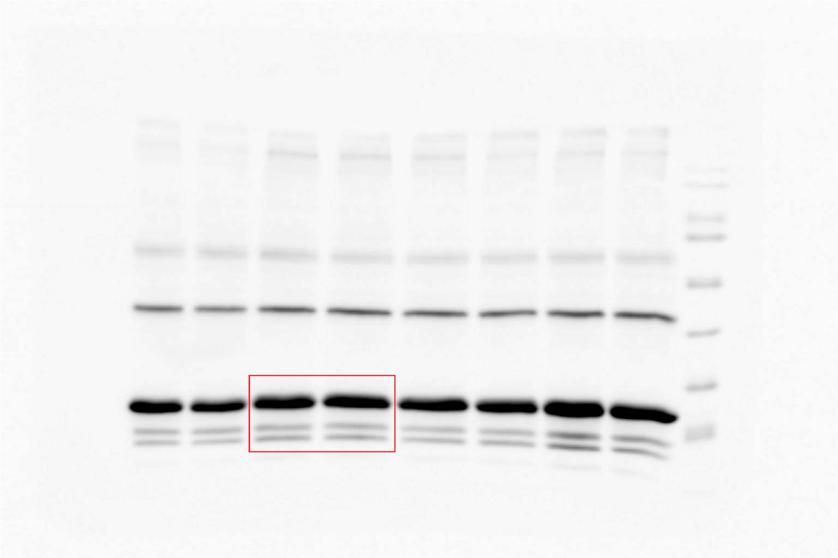

Anti-GAPDH

Figure 3 B, Figure 5 A; 8%-gel

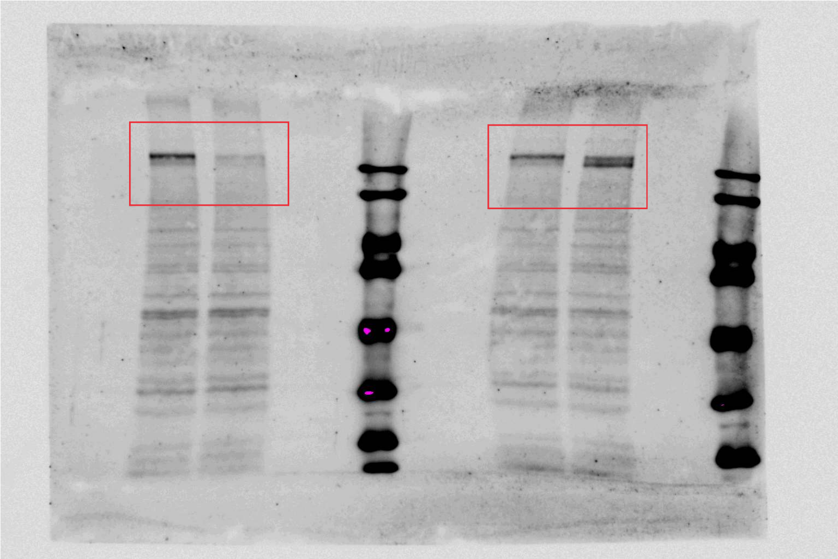

Anti-RFX7

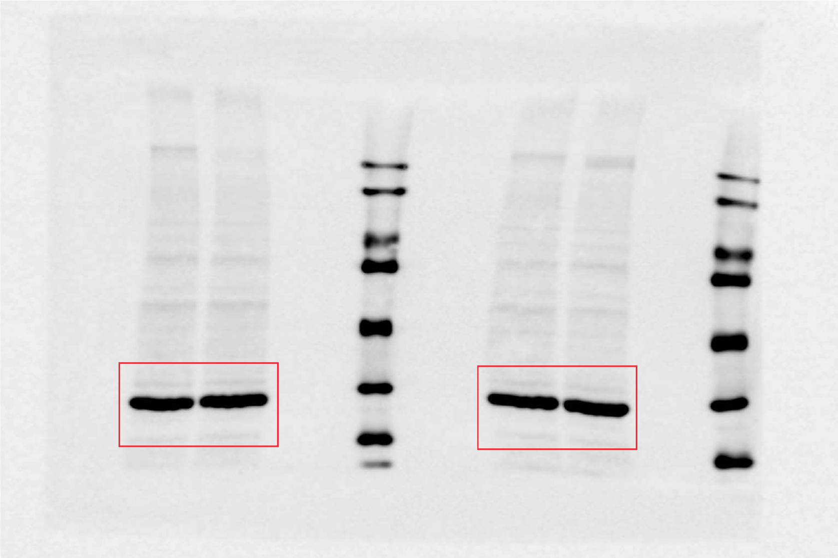

Anti-Actin

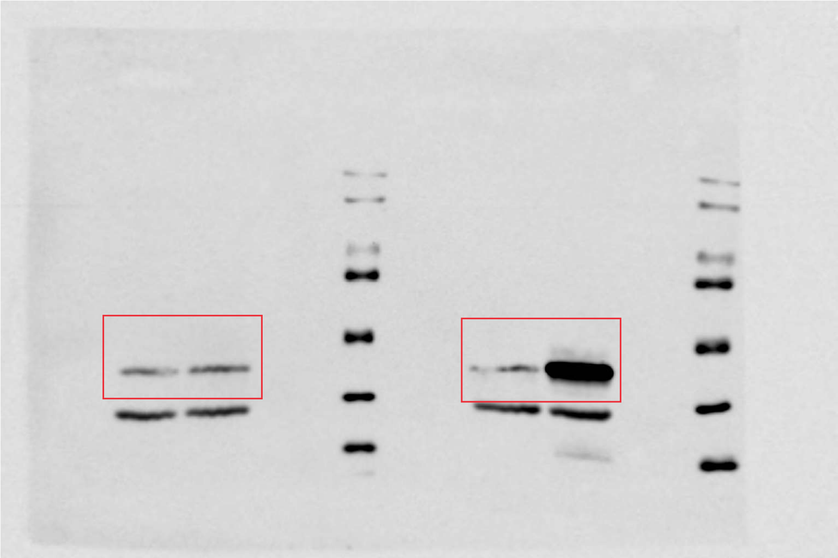

Anti-p53

Figure 3 C, D; 10%-gel

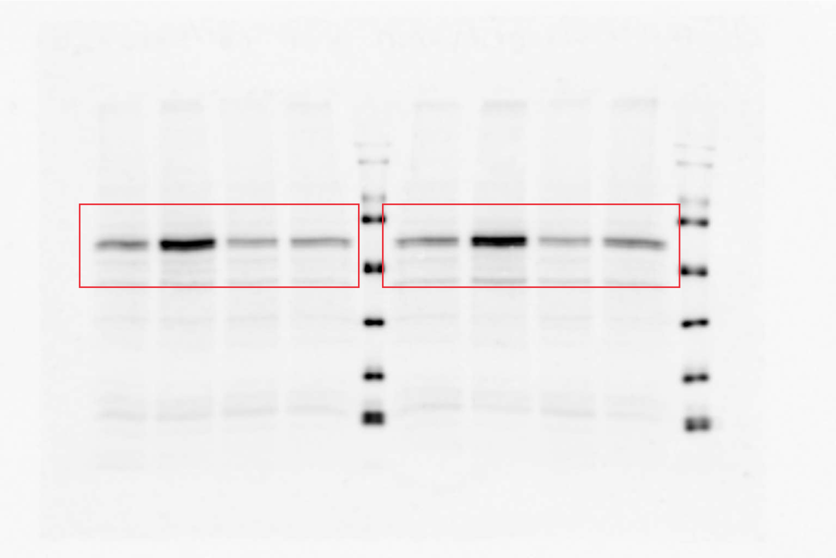

Anti-DYRK1B

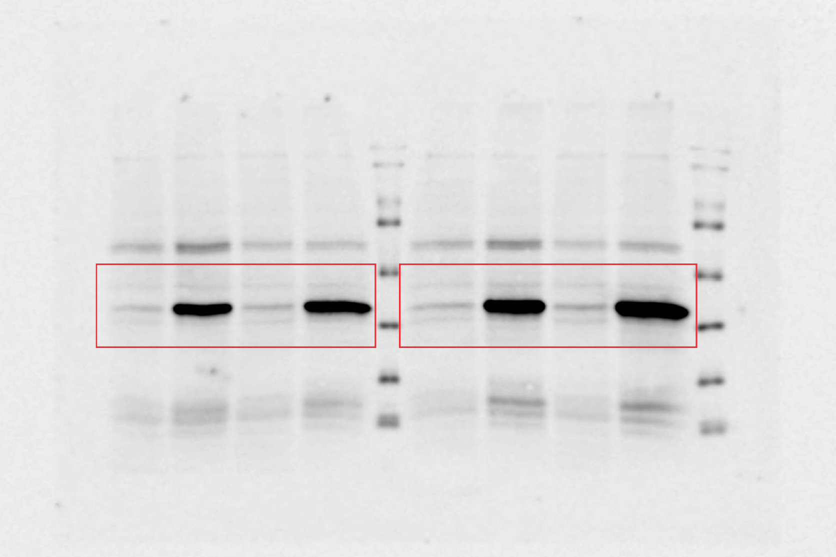

Anti-p53

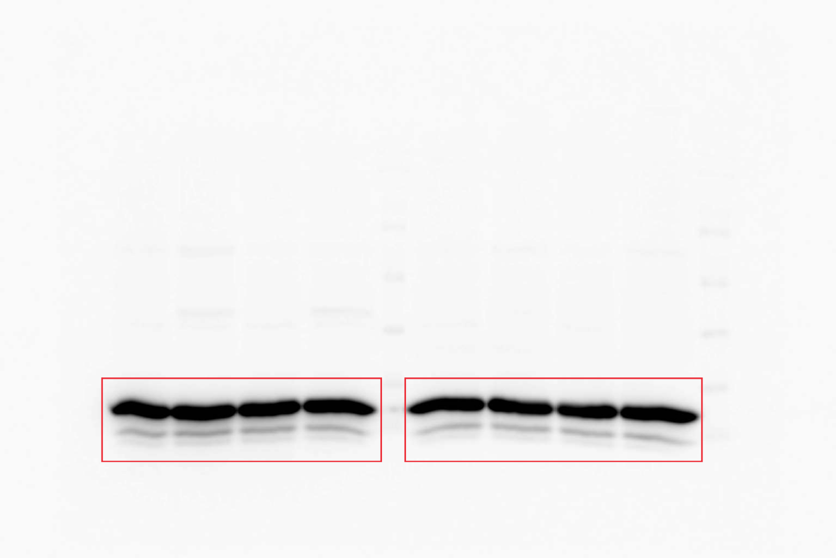

Anti-GAPDH

Figure 3 E; 10%-gel

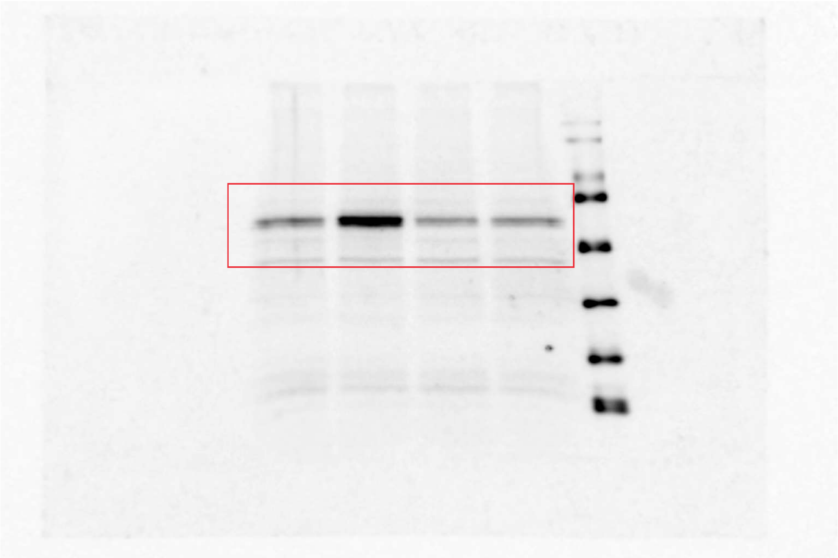

Anti-DYRK1B

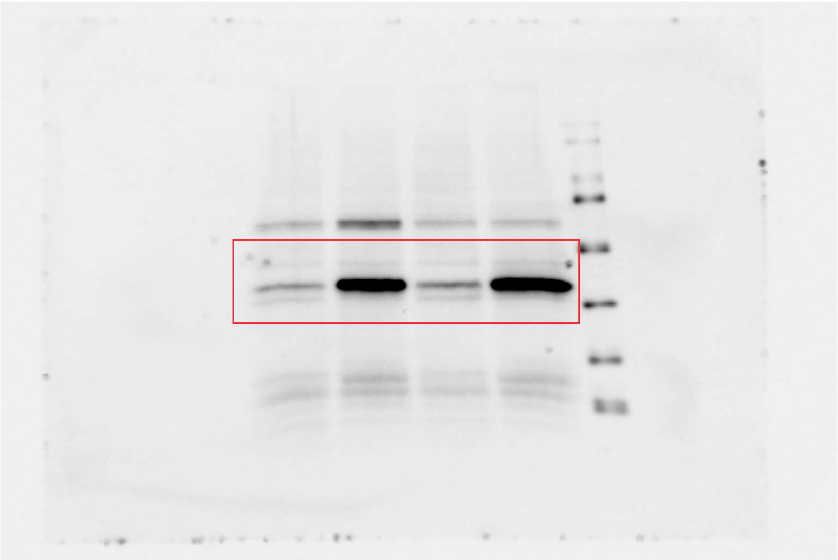

Anti-p53

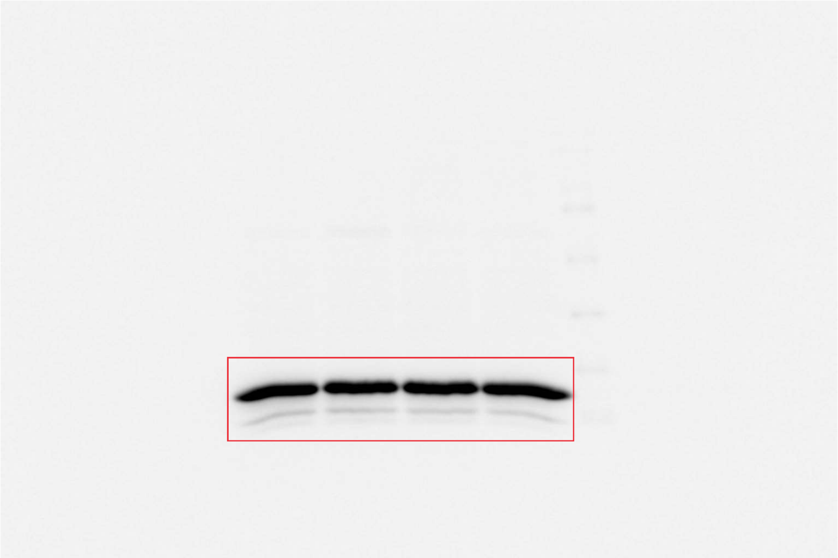

Anti-GAPDH

Figure 3 F; 8%-gel

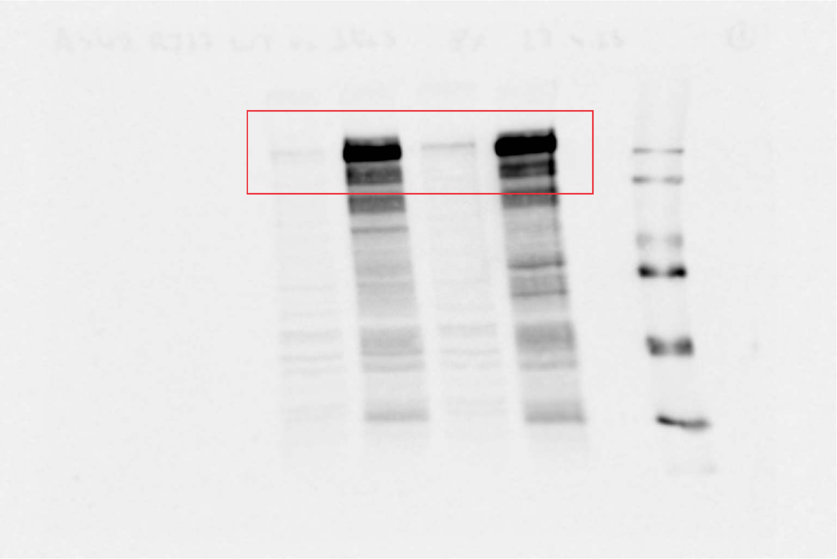

Anti-RFX7

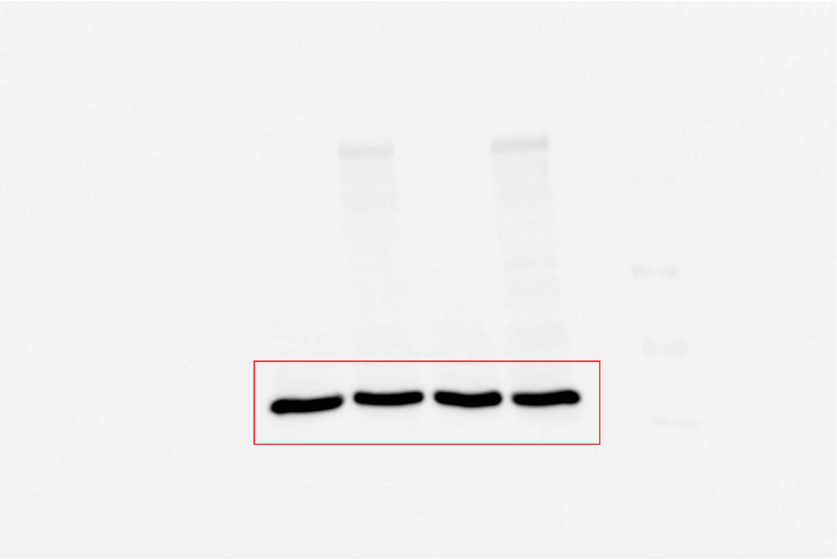

Anti-ß-Tubulin

Figure 3 F; 10%-gel

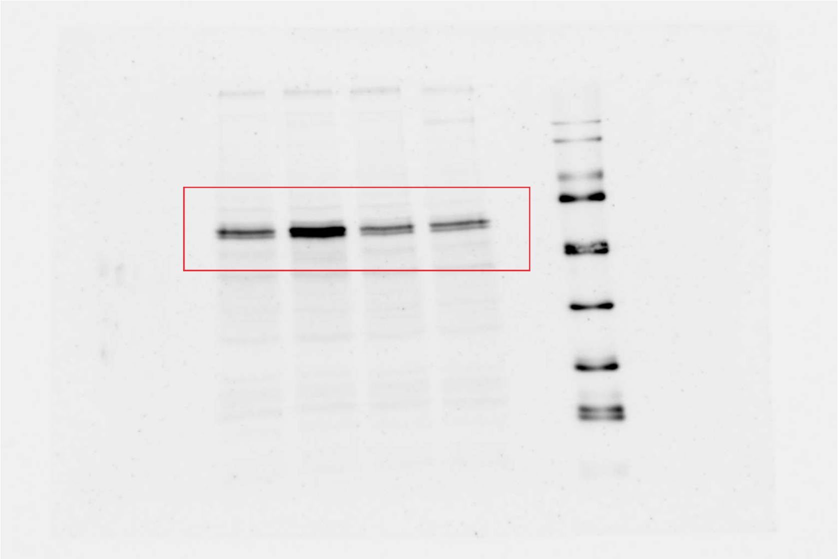

Anti-DYRK1B

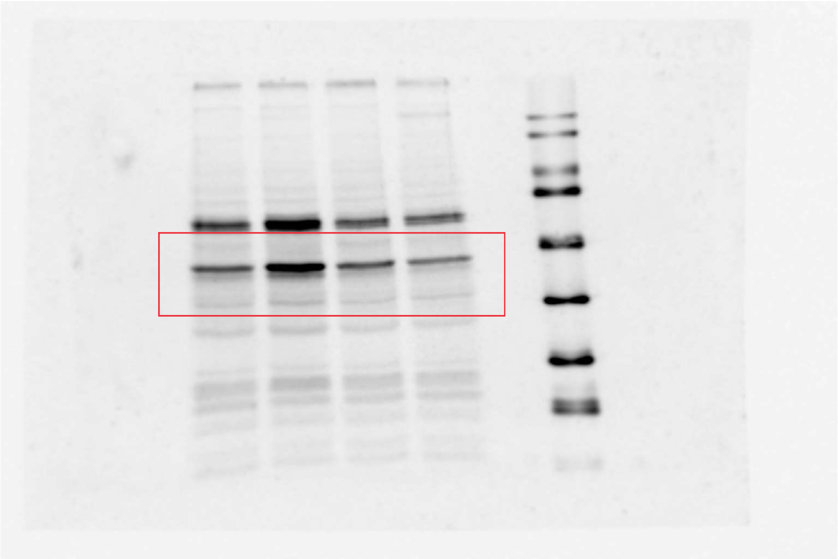

Anti-PDCD4

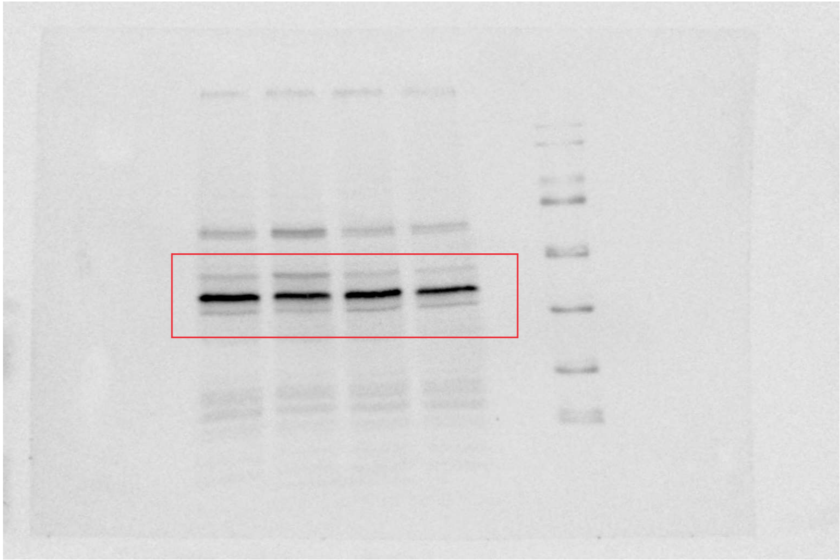

Anti-p53

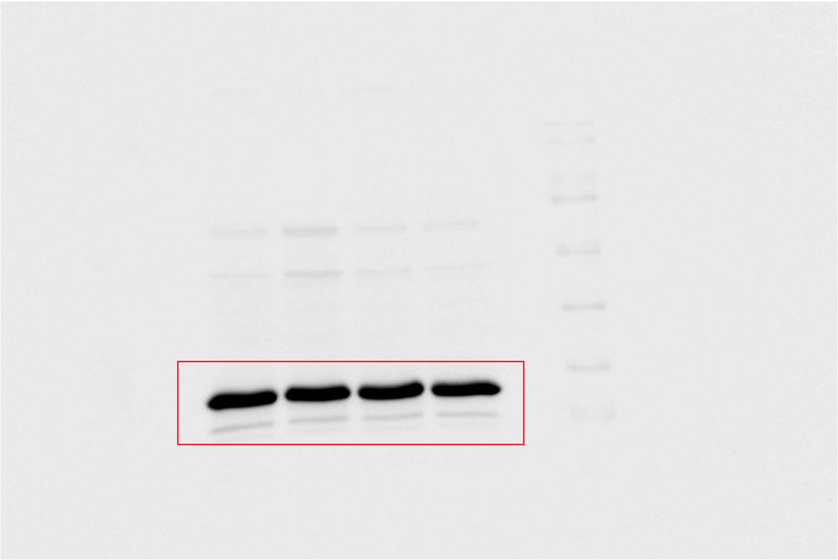

Anti-GAPDH

Figure 5 B; 8%-gel

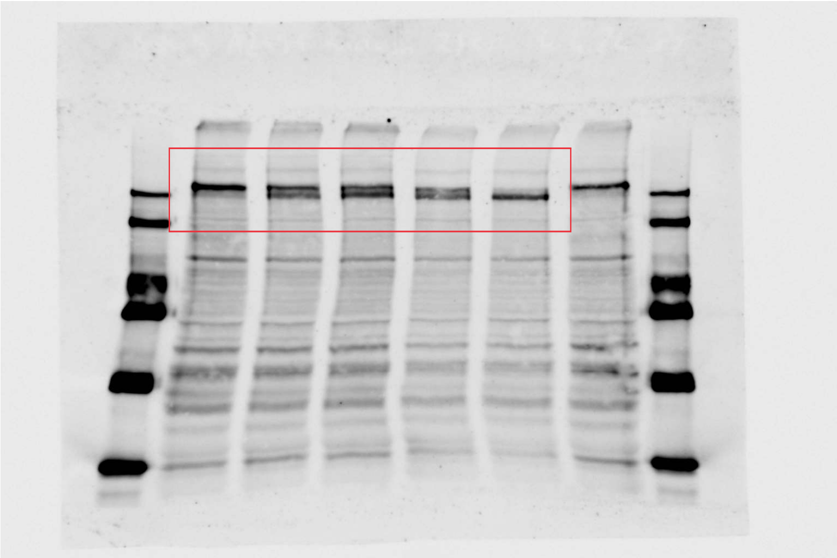

Anti-RFX7

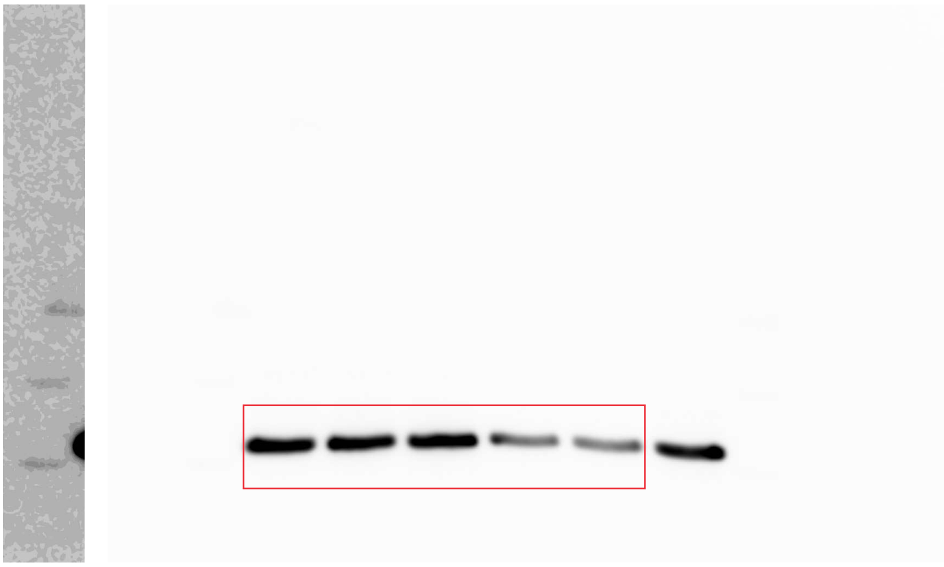

Anti-ß-Tubulin

Figure 5 B; 10%-gel

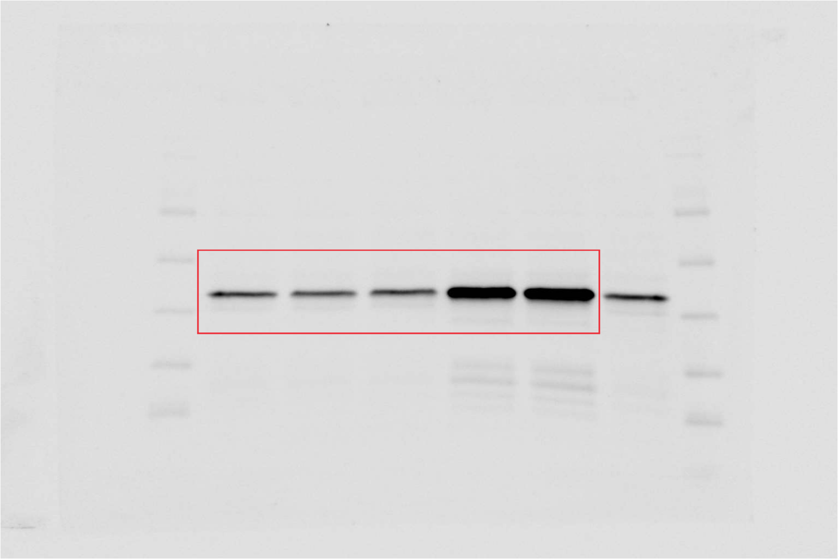

Anti-p53

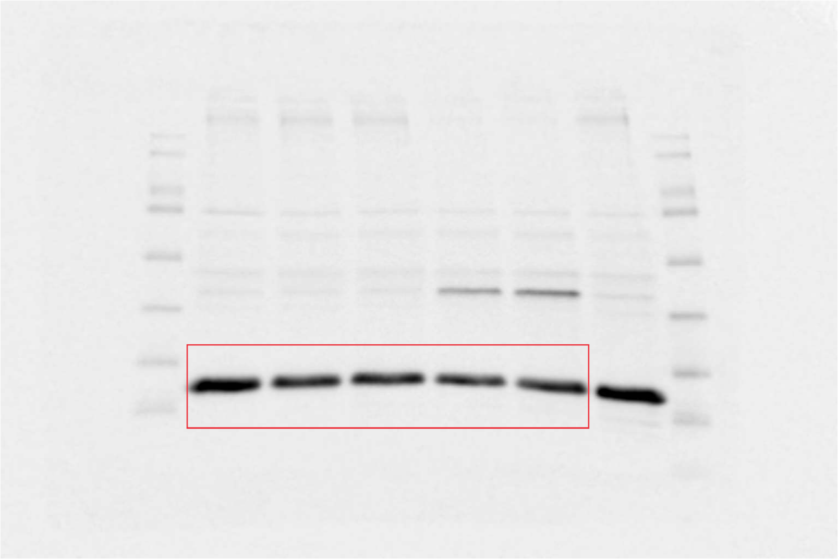

Anti-GAPDH

Figure 5 C;8%-gel

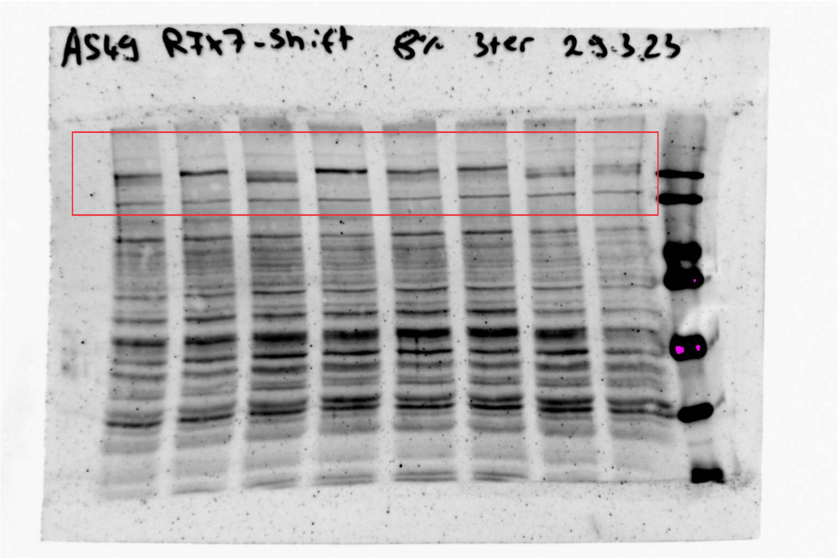

Anti-RFX7

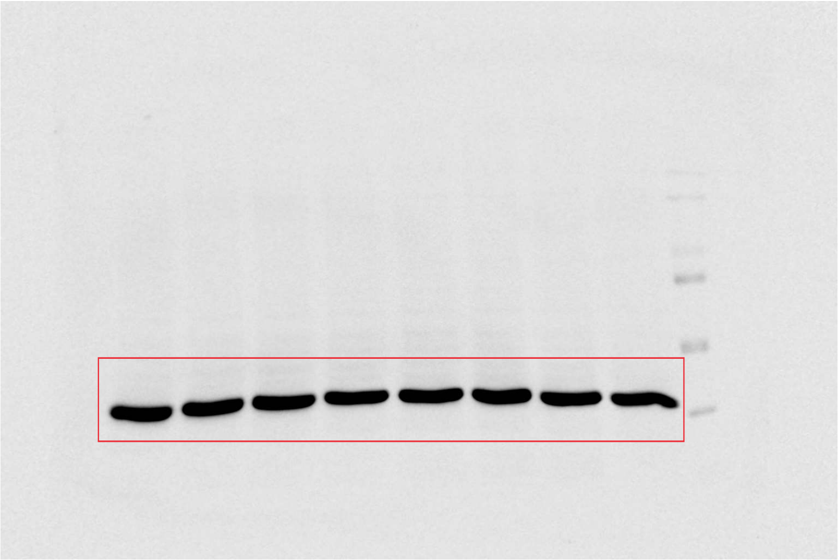

Anti-ß-Tubulin

Figure 5 C;10%-gel

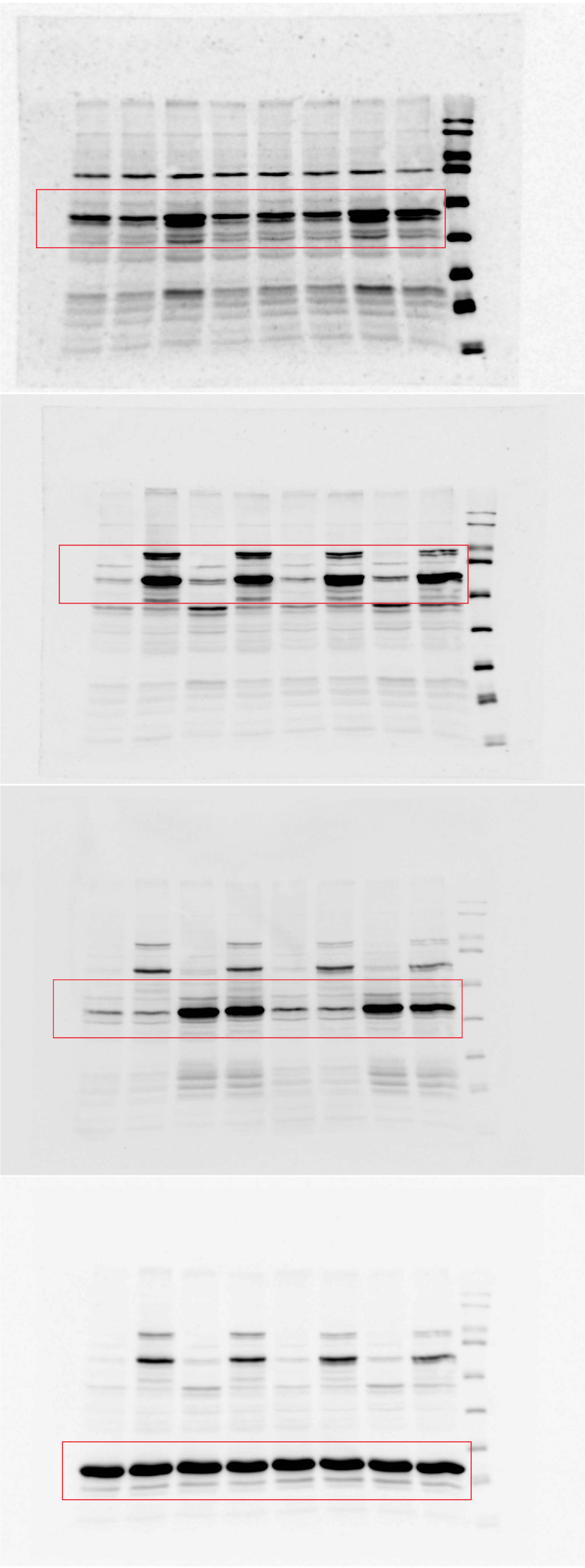

Anti-PDCD4

Anti-DYRK1B

Anti-p53

Anti-GAPDH

Figure 5 D;8%-gel

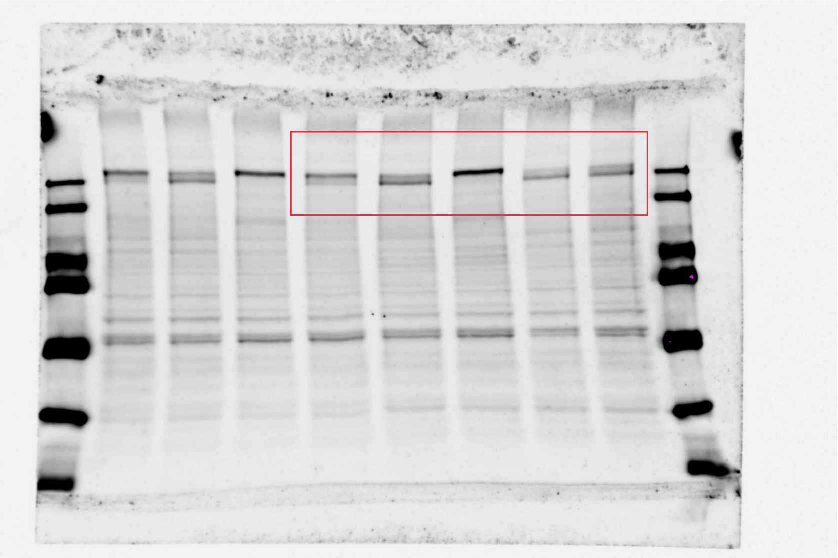

Anti-RFX7

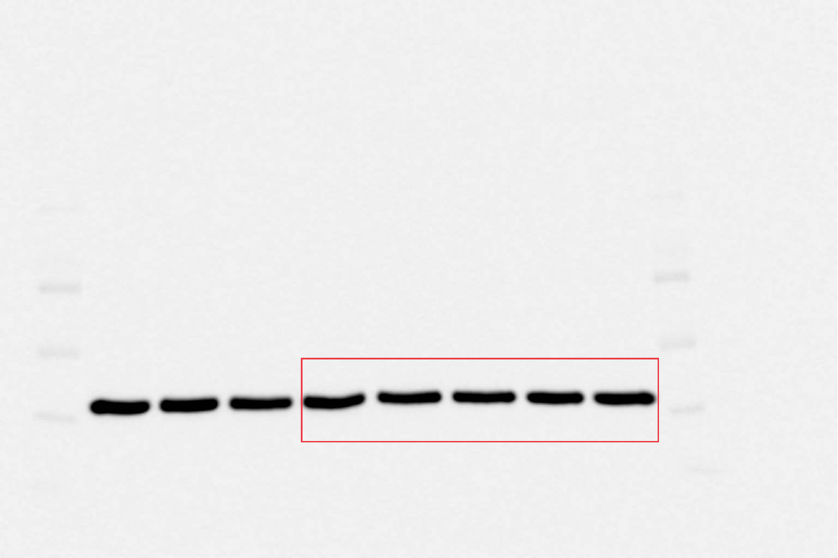

Anti-ß-Tubulin

Figure 5 D;10%-gel

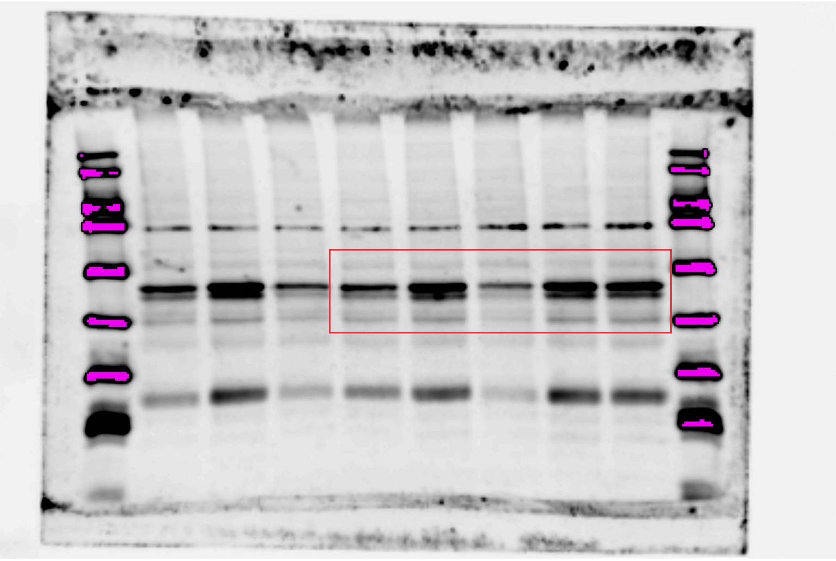

Anti-PDCD4

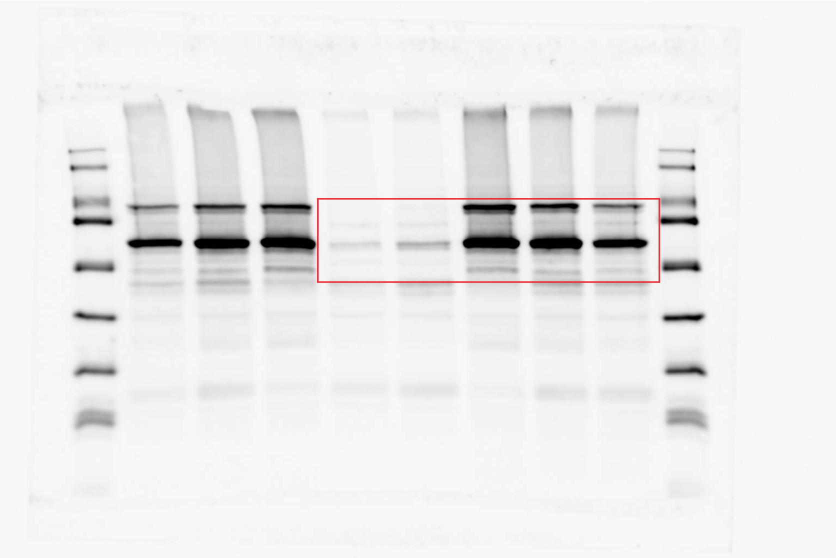

Anti-DYRK1B

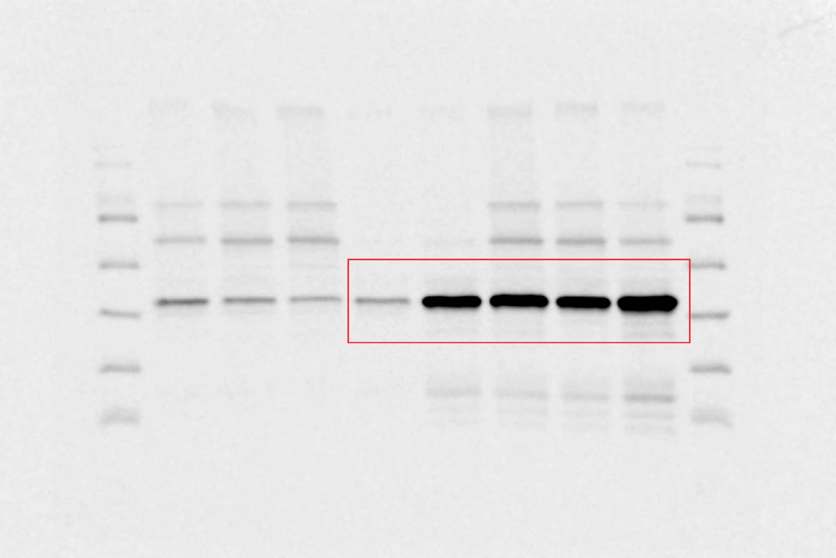

Anti-p53

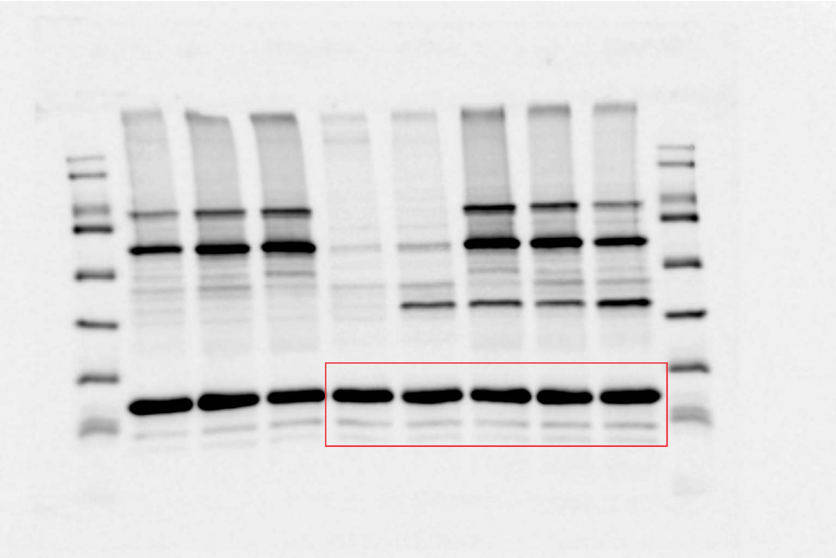

Anti-GAPDH

Figure 7 B; 8%-gel

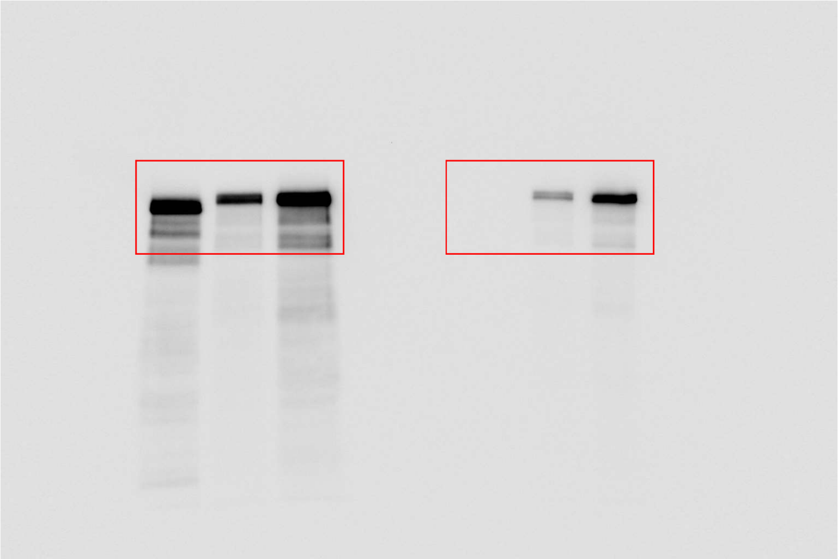

HiBiT-Blotting

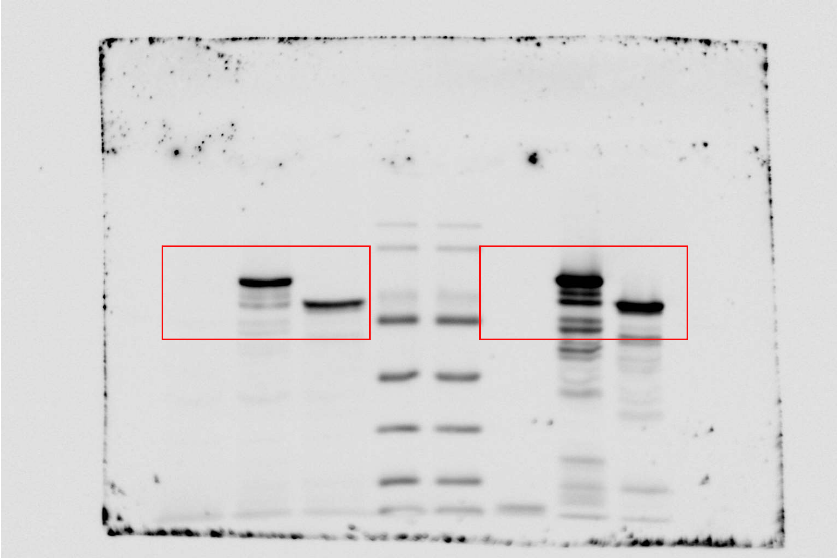

Anti-GFP

Figure 7 C; 8%-gel

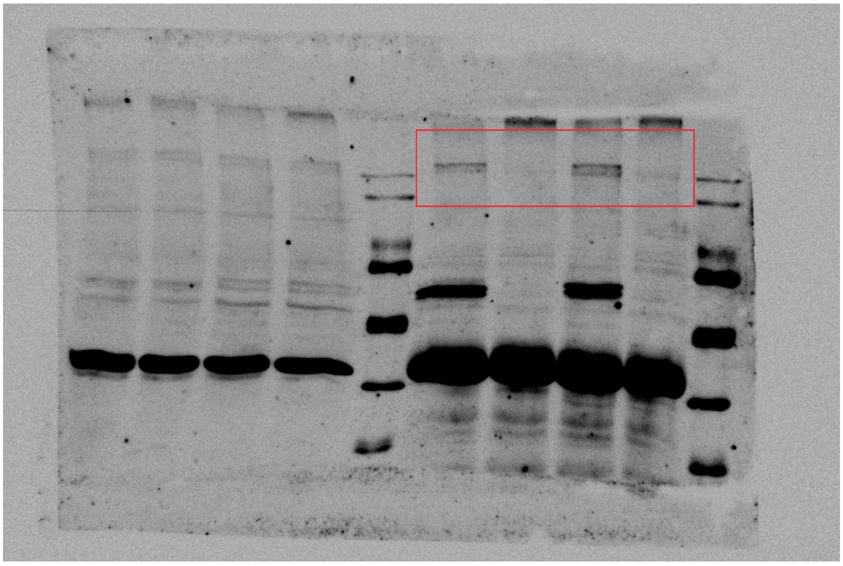

Anti-RFX7

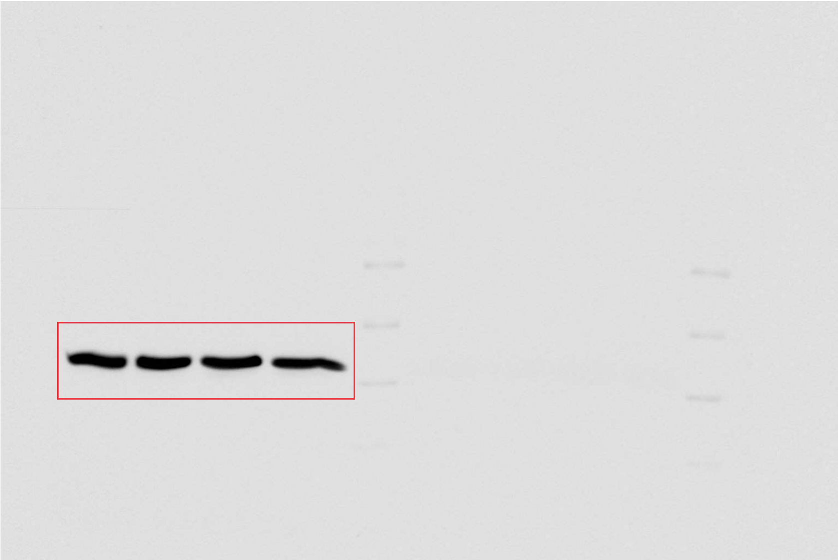

Anti-ß-Tubulin

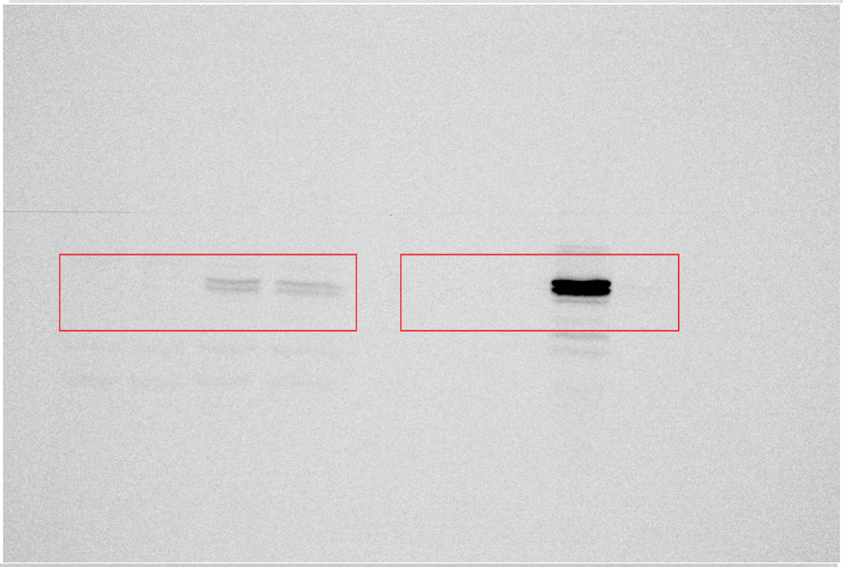

HiBiT-Blotting

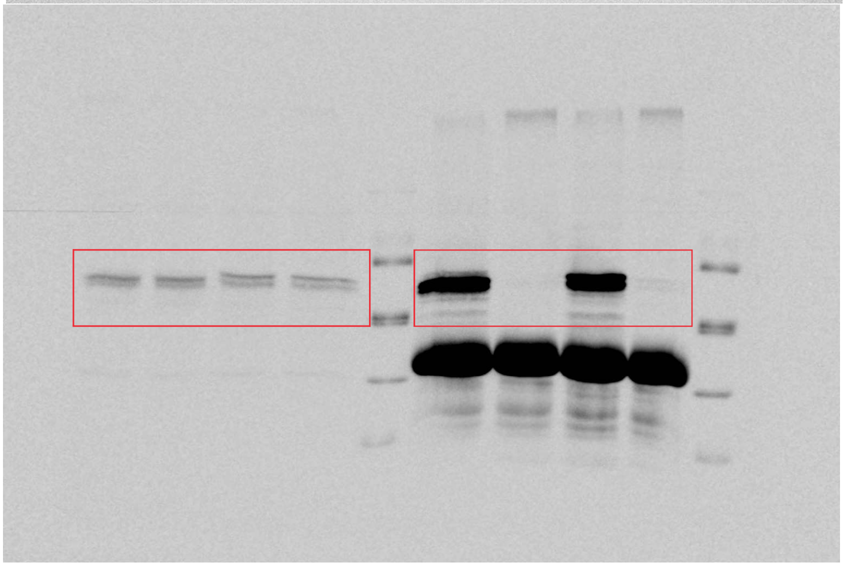

Anti-DYRK1B

Figure 7 D; 8%-gel

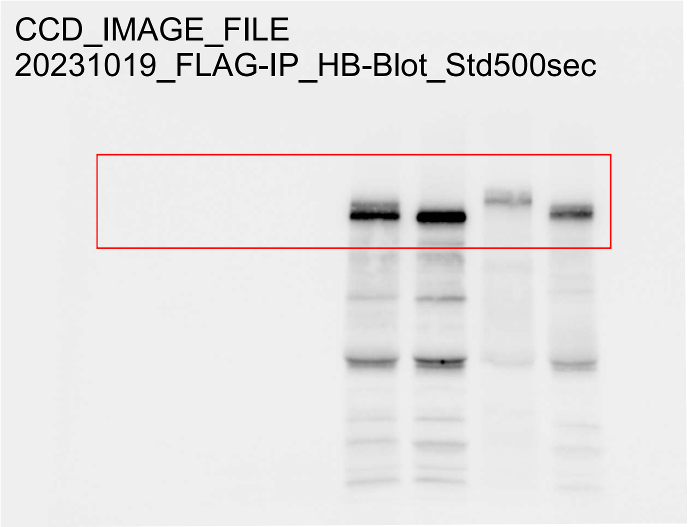

HiBiT-Blotting

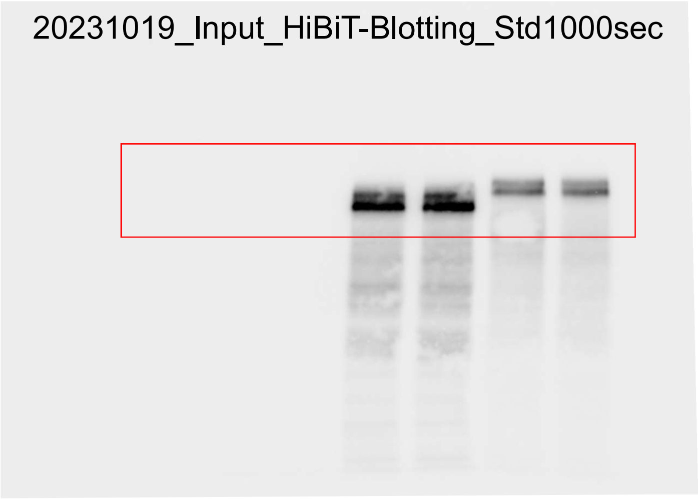

HiBiT-Blotting

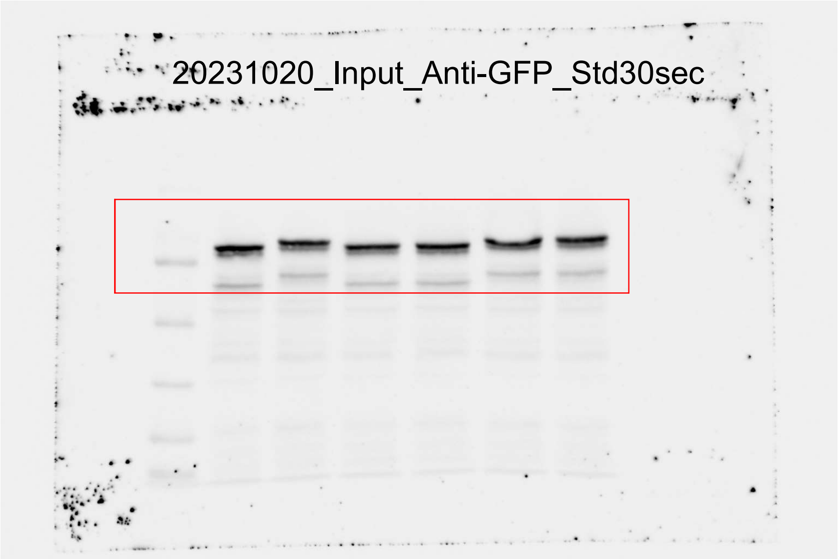

anti GFP

Figure 7 F; 8%-gel

20230615_1549_Anti-GFP_Standard30sec

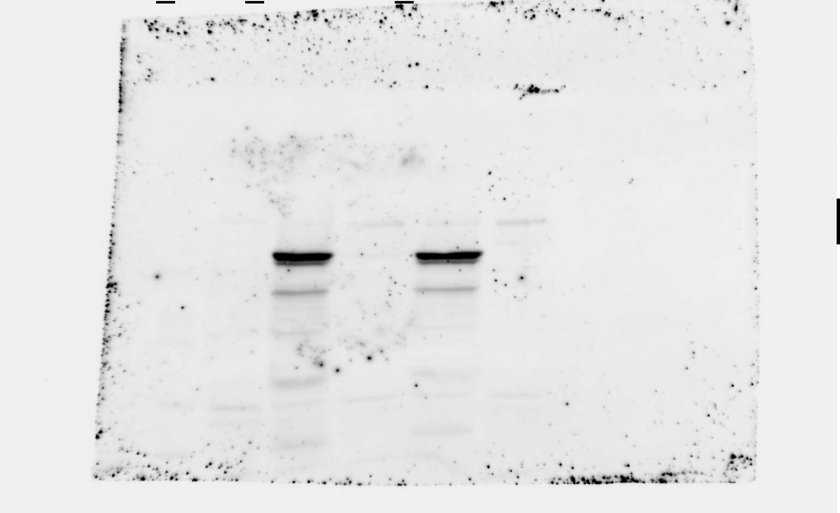

HiBiT-Blotting

HiBiT-Blotting_Standard15sec_20230422_1817.tif

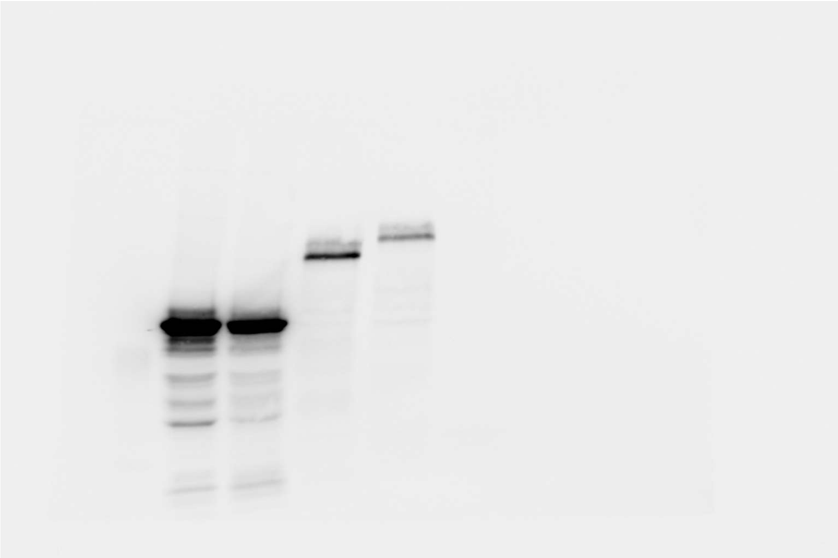

anti
GFP
