# Supplementary material for: The protein kinase DYRK1B is a p53 target gene and functions as a negative feedback regulator of the transcription factor RFX7
Source: Cell Death Dis. 2026 Mar 26;17(1):386. doi: 10.1038/s41419-026-08660-x (PMC13066115; doi:10.1038/s41419-026-08660-x)
Supplement: Supplementary file 3 — RNAseq_Readme [file 41419_2026_8660_MOESM3_ESM.docx]

**The protein kinase DYRK1B is a p53 target gene and functions as a negative feedback regulator of the transcription factor RFX7**

Dataset DOI: [10.5061/dryad.b2rbnzssz](file:///C:\Users\wbecker\Downloads\10.5061\dryad.b2rbnzssz)

**Description of the data and file structure**

RNAseq was done to analyze DEG in A549 cells treated with Nutlin-3a

**Files and variables**

**File: A549_raw_read_counts_merge.tsv.txt**

**Description:** File containing ENSBLE gene IDs in the first column and raw read counts of the different samples in the following columns.

**Variables**

- gene: ESNG ID
- A549_NT_DMSO_I: raw read counts for sample A549_NT_DMSO_I
- A549_NT_DMSO_II: raw read counts for sample A549_NT_DMSO_II
- A549_NT_DMSO_III: raw read counts for sample A549_NT_DMSO_III
- A549_NT_N3A_I: raw read counts for sample A549_NT_N3A_I
- A549_NT_N3A_II: raw read counts for sample A549_NT_N3A_II
- A549_NT_N3A_III: raw read counts for sample A549_NT_N3A_III
- A549_NT_N3A_IIII: raw read counts for sample A549_NT_N3A_IIII

**File: A549_DMSO_vs_N3A_DEG.txt**

**Description:** File containing the DEG analysis of A549 cell treated with DMSO (control) or Nutlin-3a (N3a)

**Variables**

- gene_symbol: HGNC gene symbol
- logFC: log2 fold change in expression DMSO vs N3a
- AveExpr: average gene expression
- t: t-value from t testing
- P.Value: p-value from t testing
- adj.P.Val: FDR
- B: beat value (limma)

**Code/software**

Experimental procedure:

Data were aligned to the GRCh38p14 genome and counted with STAR Aligner (2.7.10b) and further analyzed and visualized with BioJupies (<https://github.com/MaayanLab/biojupies>) using default parameters. For differential expression (DE) analysis three independent biological replicates (n=3) of each cell line were used. To calculate DE, we compared the different cell lines against each other. BioJupies uses *limma* for differential gene expression analysis. In brief, raw read counts are converted to CPM and normalized by the voom function. A gene-wise linear model was used to assess DE. DE is estimated by an empirical Bayes framework, including a robust shrinkage, before utilizing t-statistics for DE assessment.

**Access information**

Other publicly accessible locations of the data:

- n/a

Data was derived from the following sources:

- n/a
